# Association between population distribution and urban GDP scaling

**DOI:** 10.1371/journal.pone.0245771

**Published:** 2021-01-22

**Authors:** Haroldo V. Ribeiro, Milena Oehlers, Ana I. Moreno-Monroy, Jürgen P. Kropp, Diego Rybski

**Affiliations:** 1 Departamento de Física, Universidade Estadual de Maringá, Maringá, PR, Brazil; 2 Potsdam Institute for Climate Impact Research – PIK, Member of Leibniz Association, Potsdam, Germany; 3 OECD, Paris, France; 4 Department of Geography and Planning, University of Liverpool, Chatham St, Liverpool, United Kingdom; 5 Institute for Environmental Science and Geography, University of Potsdam, Potsdam, Germany; 6 Department of Environmental Science Policy and Management, University of California Berkeley, Berkeley, CA, United States of America; China University of Geosciences, CHINA

## Abstract

Urban scaling and Zipf’s law are two fundamental paradigms for the science of cities. These laws have mostly been investigated independently and are often perceived as disassociated matters. Here we present a large scale investigation about the connection between these two laws using population and GDP data from almost five thousand consistently-defined cities in 96 countries. We empirically demonstrate that both laws are tied to each other and derive an expression relating the urban scaling and Zipf exponents. This expression captures the average tendency of the empirical relation between both exponents, and simulations yield very similar results to the real data after accounting for random variations. We find that while the vast majority of countries exhibit increasing returns to scale of urban GDP, this effect is less pronounced in countries with fewer small cities and more metropolises (small Zipf exponent) than in countries with a more uneven number of small and large cities (large Zipf exponent). Our research puts forward the idea that urban scaling does not solely emerge from intra-city processes, as population distribution and scaling of urban GDP are correlated to each other.

## Introduction

Physical measurements such as weight or size of objects are always confined to specific scales. However, the outcomes of several natural phenomena and socio-economic processes can extend across multiple orders of magnitude [1]. Examples of such systems are as diverse as earthquakes [2], stock market fluctuations [3], casualties in human insurgencies [4], and fracture of materials [5]. These systems usually share non-trivial statistical regularities manifested in the form of power-law distributions or power-law relations, the so-called scaling laws. Cities are among these systems as they occur in sizes from thousands to tens of million inhabitants. Urban systems are also well known to follow scaling relations in time and space, as summarized by Batty [6] in the “seven laws of urban scaling”.

Zipf’s law [7, 8] and Bettencourt-West law [9] are two of the best-known scaling laws emerging in urban systems. Initially observed by Auerbach [8] and then popularized by Zipf [7], Zipf’s law for cities states that the distribution of city populations, for a given region or country, is approximated by an inverse power-law function (or the rank-size rule), implying that there are plenty of small cities and very few metropolises. Bettencourt-West law [9], better known as “urban scaling”, establishes a power-law relation between urban indicators and city population. Urban scaling is well illustrated by the concept of agglomeration economies in cities [10, 11], in which the power-law association between urban wealth and population implies that urban wealth increases more than proportionally with city population.

Fostered by a recent deluge of highly-detailed city data, researchers from diverse disciplines such as geography, economics, or physics have devoted ongoing efforts in identifying and understanding fundamental principles and regularities underlying urban systems [1, 6, 12–14]. While most of these works are either concerned with Zipf’s law [15–25] or urban scaling [26–34], very few have tackled the relationship between both. Zipf’s law and urban scaling have mostly been studied independently because it is commonly assumed that both laws are independent descriptions of urban systems across countries. The few investigations on possible connections between Zipf’s law and urban scaling have a more local character and explore this association within countries [35, 36]. The work of Gomez-Lievano *et al*. [35] is seminal in this regard and has shown that (under certain conditions) urban scaling can be related to Zipf’s law when urban indicators are also power-law distributed. This connection is obtained from probability distributions and shows that the urban scaling exponent has an upper limit determined by both the Zipf exponent and the power-law exponent that characterizes the urban indicator distribution. This result however does not imply that these three exponents are directly associated with each other. In fact, random permutations of population and urban indicator values only have the effect of removing a possible correlation between the two variables, but do not have the effect of changing their distribution.

To date, there has been no attempt to empirically investigate the association between urban scaling and Zipf’s law across a large number of countries. The paucity of such studies certainly reflects the lack of consistent and comparable data across countries. A convincing comparison between these two laws demands a unified city definition across the globe as well as measures of population and urban indicators based on this same definition. And it was not until very recently that satisfying this requirement has become possible. Here we use a new harmonized city definition for investigating Zipf’s law and urban scaling over almost five thousand cities in 96 countries. By analyzing population and gross domestic product (GDP) of these cities per country, we estimate the Zipf and the urban scaling exponents to probe for a possible relation between these two scaling laws. Our empirical results show that both exponents are indeed related to each other and that a functional form of this association can be exactly derived from scaling relations emerging at the country level.

We demonstrate that Zipf’s law and urban scaling imply a power-law relation between total urban population and total urban GDP of countries, where the country scaling exponent is dependent on the Zipf and the urban scaling exponents. Because country-aggregated values of urban population and GDP are fixed, there is only one country scaling exponent for total GDP, which in turn associates the urban scaling exponent to the Zipf exponent. We verify the integrity of our model by estimating the country-scaling exponents from the empirical relationship between the Zipf and the urban scaling exponents, and also by showing that numerical simulations yield results very similar to those obtained from real-world data. The connection between both exponents shows that urban scaling does not only emerge from processes occurring within the city boundaries; instead, it suggests that the population distribution of an urban system does affect urban scaling or vice versa. For the particular case of urban GDP, while almost all countries exhibit increasing returns to scale (urban scaling exponent greater than one), our findings indicate this effect is smaller in countries with a more balanced number of small and large cities (small Zipf exponent) than in countries with a more unbalanced number of small and large cities (large Zipf exponent).

## Results

### Empirical connection between both scaling laws

We start by revisiting Zipf’s law and the urban scaling law. Zipf’s law for cities [7] establishes that the rank *r* and the city population *s* of an urban system are related through a power-law function

$$\begin{array}{c} r∼s^{-\alpha }\;, \end{array}$$ (1)

where *α* > 0 is the Zipf exponent. The estimated *α* varies across countries and epochs, but *α* ≈ 1 is typically found in empirical studies [18, 23]. Urban scaling laws [9] commonly refer to power-law associations between an urban indicator *y* and the city population *s* within an urban system, that is,

$$\begin{array}{c} y=c\;s^{\beta }\;, \end{array}$$ (2)

where *c* > 0 is a constant and *β* is the urban scaling exponent. The value of *β* depends on the type of urban indicator, but increasing returns to scale (*β* > 1) are usually reported for socio-economic indicators, decreasing returns to scale (*β* < 1) for infrastructure indicators, and constant returns to scale (*β* = 1) for indicators related to individual needs [9]. The values of *α* and *β* are also susceptible to different city definitions [23, 37], and we thus need a unified definition across different urban systems to test a possible association between their values.

**Fig 1:**
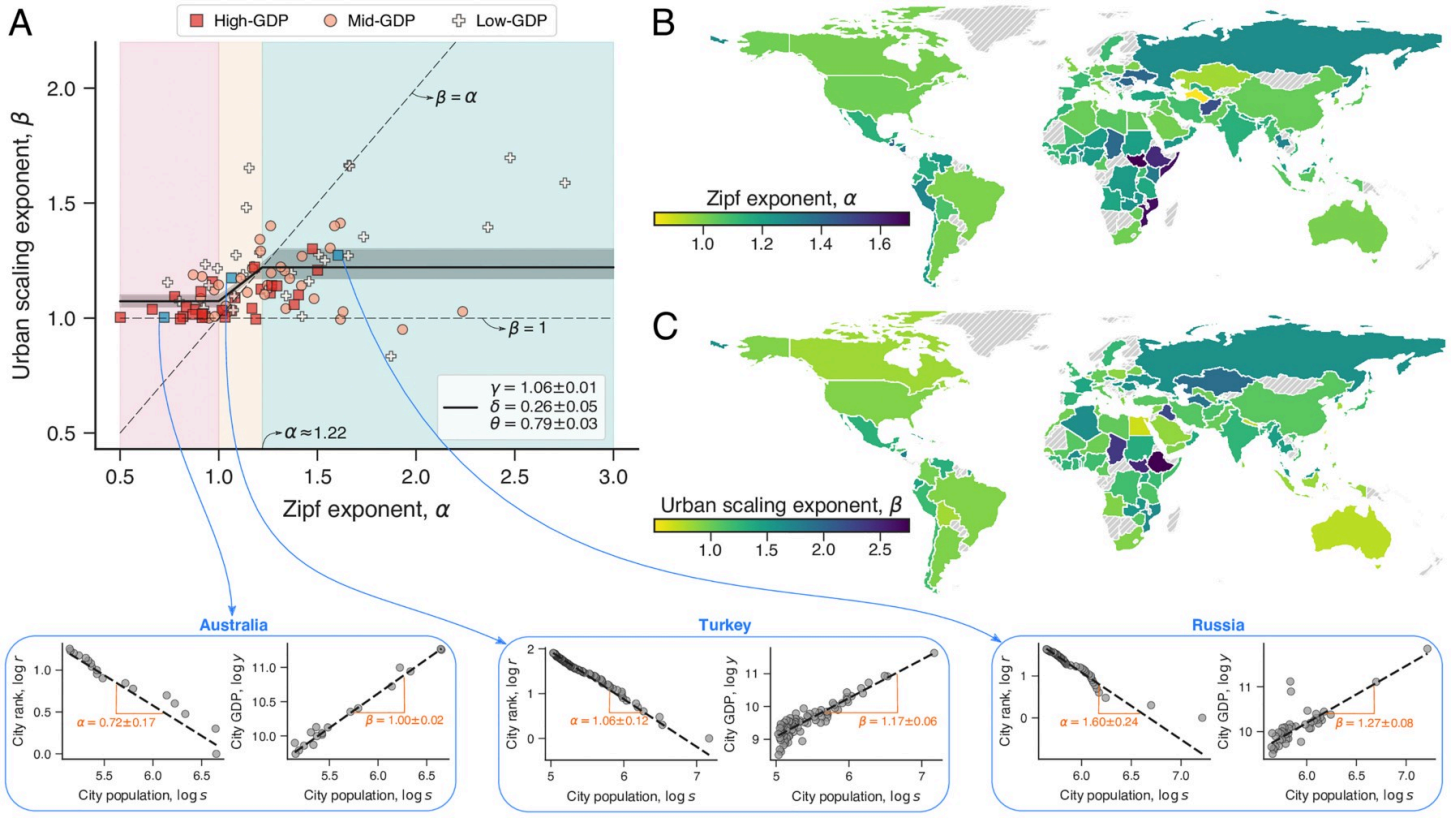
Association between the urban scaling exponent *β* and the Zipf exponent *α*. (A) Values of *β* versus *α* for each country in our data set. The three different markers distinguish countries according to the tercile values of the total urban GDP distribution (for instance, high-GDP countries have highest ≈33% GDP values). The horizontal dashed line shows the *β* = 1 while the inclined dashed line represents the *β* = *α* relationship. As we shall discuss in the next section, the continuous line shows the model of Eq (9) adjusted to data and the gray shaded region stands for the 95% confidence band. The colored background represents the different intervals of *α* defined in Eq (9). The insets (indicated by blue arrows) illustrate Zipf’s law for city population (left) and the urban scaling relationship of city GDP (right) for three countries (see S1 Appendix, Sec. 4 for all considered countries). We have verified that the model of Eq (9) provides a significantly better description of data when compared with a null model where *β* is independent of *α* (intercept-only model: *β* = constant, see Fig 4 in S1 Appendix), and that statistical correlations between *β* and *α* are significantly enhanced in the (orange shaded) region where our model predicts a linear correspondence between the exponents (Fig 5 in S1 Appendix). Maps in panels (B) and (C) show the color-coded values of *α* and *β* for each country (light gray indicates missing values).

To do so, we use a generalized definition of functional urban areas (FUA) recently proposed by Moreno-Monroy *et al*. [38, 39]. The concept of FUA was initially developed for countries of the OECD and Europe as a unified definition of metropolitan areas, consisting of high-density urban cores and their surrounding areas of influence or commuting areas. The generalized definition we use (so-called eFUA or GHSL-FUA) represents an extension of this concept to countries of the entire globe. By considering the areas of influence of urban cores, eFUAs give a less fragmented representation of the city size distribution because dense clusters proximate to urban cores are rightly not considered as independent cities. While eFUAs delineate world-wide comparable city boundaries, the majority of urban indicators are available at the level of local administrative units, that in addition to not being centralized into a global data set, may change from country to country. We avoid this problem by considering a global gridded data set for GDP [40] (see Materials and methods). Thus, by combining these two data sources (see Materials and methods and Fig 1 in S1 Appendix), we create a consistent data set comprising the population (*s*) and the GDP (*y*) of 4,571 cities from 96 countries.

**Fig 2:**
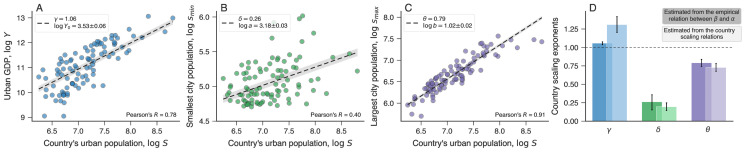
Country scaling relationships. (A) Scaling relation between total urban GDP (*Y*) and total urban population (*S*) for all countries in our data set. (B) Scaling law between the smallest city population of a country (*s*_min_) and the country’s urban population (*S*). (C) Scaling law between the largest city population of a country (*s*_max_) and the country’s urban population (*S*). In these three panels, markers represent the values for each country and the dashed lines are the country scaling relationships of Eqs (5), (6) and (8), where the exponents *γ*, *δ*, and *θ* are obtained from fitting the model of Eq (9) to the empirical relation between *β* and *α*. Only the constants *Y*_0_, *a* and *b* are adjusted to data and their best fitting values are shown in the panels (± standard errors). (D) Comparison between estimates of the country scaling exponent obtained by fitting Eq (9) to the empirical association between *β* and *α* (bars in dark colors) and by directly fitting the values of the country scaling relationships (bars in light colors, see Fig 6 in S1 Appendix for the adjusted scaling laws). Error bars represent 95% bootstrap confidence intervals of the parameters. We notice that both approaches yield similar estimates, which are statistically indistinguishable in the cases of *δ* and *θ*.

Having this harmonized data set, we estimate the Zipf exponent *α* and urban scaling exponent *β* after grouping the values of *s* and *y* by country (see Materials and methods). The scatter plot in Fig 1A depicts the values of *β* versus *α*, where the insets show Zipf’s law and urban scaling of city GDP for three countries. As these three examples illustrate, the data follows Zipf’s law and urban scaling with deviations comparable with other studies about these two laws (see S1 Appendix, Sec. 4 for all countries). The world maps in Fig 1B and 1C indicate the regional distribution of the values of *α* and *β*. In line with the meta-analysis of Refs. [18, 23], we find Zipf exponents *α* roughly distributed around 1 with average value and standard deviation equal to 1.24 and 0.38, respectively (Fig 2A in S1 Appendix). In turn, ≈94% of the countries exhibit urban scaling exponents with GDP (*β*) larger than 1 (Fig 2B in S1 Appendix), and the average value and standard deviation of *β* are 1.16 and 0.17, respectively. This result agrees with the idea that economic indicators display increasing returns to scale [9, 41].

**Fig 3:**
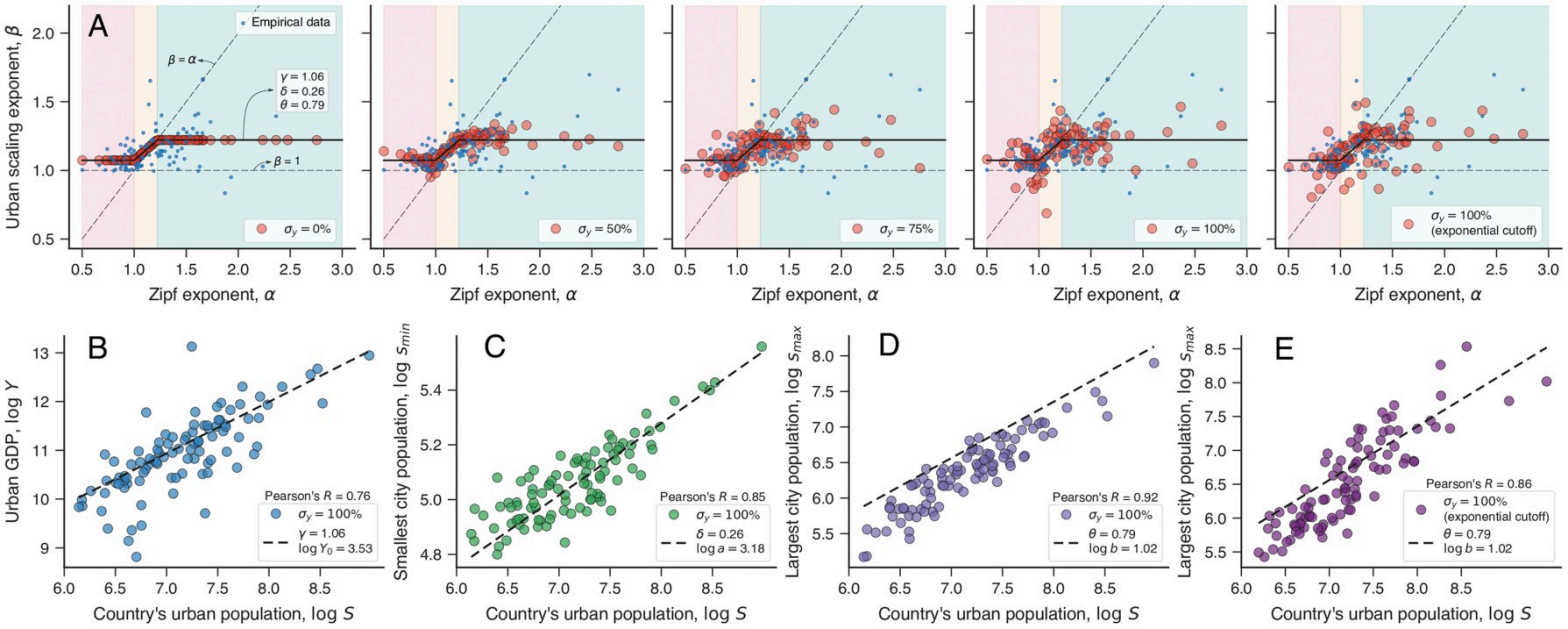
Simulating the connection between *α* and *β*, and the country scaling relationships. (A) Simulated relationships between *α* and *β* under different levels of random variation in the urban scaling law (Eq 2). Here *σ*_*y*_ is the percentage of the standard deviation in the empirical values of *β*. We have a perfect agreement between simulations and the model of Eq (9) when *σ*_*y*_ = 0%, and the results become very similar to the empirical data (small blue dots) as the intensity of the random variation increases. (B)-(D) Simulated country scaling laws for *σ*_*y*_ = 100%. The dashed lines represent the scaling relationships of Eqs (5), (6) and (8), with parameters estimated from empirical data. We notice that the simulated scaling laws of total urban GDP (*Y*) and smallest city population (*s*_min_) follow well the empirically adjusted relationships, while the simulated values for the largest city population (*s*_max_) underestimate the empirical values. This occurs because large populations are rare and do not get close enough to the imposed maximum *s*_max_. (E) Scaling relationship between *s*_max_ and *S* when considering that city population values are drawn from a power-law distribution with exponential cutoff. We notice this change makes the simulated results very similar to the empirical ones. The right-most plot in panel A shows a simulated relation between *β* and *α* when considering the exponential cutoff.

The results shown in Fig 1 suggest a positive association between the values of *β* and *α*, that is, an increase of the Zipf exponent *α* appears to come along with a rise in the urban scaling exponent *β* (or vice versa). This behavior is also perceptible in the world maps, where we note that regional differences in the values of *α* (Fig 1B) are similar to the ones observed for *β* (Fig 1C). However, these visual similarities are mainly induced by the largest countries in land area, and a more careful analysis of these maps reveals important differences, especially in the African continent and Central Asia. These differences are more evident in the scatter plot of Fig 1A, where we observe a considerable spread in the association between both exponents. We have further quantified the association between the values of *β* and *α* by estimating the Spearman rank correlation and the Pearson correlation within a sliding window of size Δ*α*′ centered in *α*′. For different values of Δ*α*′, we find that the correlations peak around *α*′ ≈ 1.1 and start to decrease and become not statistically significant as the sliding window moves away from this value of *α*′ (Fig 3 in S1 Appendix). This analysis thus suggests that the overall association between both exponents is non-linear and that *β* may approach constant values for large and small values of *α*.

### Country scaling and the association between both exponents

To better describe the empirical connection between the urban scaling exponent and the Zipf exponent, we hypothesize that this association can be derived from the relationship between country-aggregated values of urban population (*S*) and urban GDP (*Y*). As we shall show, the combination of Zipf’s and the urban scaling laws implies a power-law relation between total urban population *S* and total urban GDP *Y*. This country scaling law is characterized by an exponent *γ* that is a function of *β* and *α*, which in turn yields a mathematical expression for the relation between *β* and *α*. To do so, we start by noticing that Zipf’s law implies a power-law dependence for the absolute number of cities with population *s*, that is, *p*(*s*) = *ks*^−(*α*+1)^ for *s*_min_ < *s* < *s*_max_, where *s*_min_ and *s*_max_ represent a lower and an upper cutoff associated to the smallest and largest city population of a particular urban system, and *k* is a normalization constant (see S1 Appendix, Sec. 1 for details). By combining this frequency function *p*(*s*) with urban scaling (Eq 2), we can write the total urban GDP of a country as

$$\begin{array}{c} Y=\sum_{s=s_{\text{min}}}^{s_{\text{max}}}cs^{\beta }p\left(s\right)\approx kc\int_{s_{\text{min}}}^{s_{\text{max}}}s^{\beta -\alpha -1}ds\;, \end{array}$$ (3)

where the normalization constant *k* is determined by

$$\begin{array}{c} S=\sum_{s=s_{\text{min}}}^{s_{\text{max}}}sp\left(s\right)\approx k\int_{s_{\text{min}}}^{s_{\text{max}}}s^{-\alpha }ds\;. \end{array}$$ (4)

To solve these equations, we consider that *s*_min_ and *s*_max_ are power-law functions of *S*

$$s_{\text{min}}=aS^{\delta },$$ (5)

$$s_{\text{max}}=bS^{\theta },$$ (6)

where *a* > 0 and *b* > 0 are constants, and *δ* > 0 and *θ* > 0 are country scaling exponents. Thus, by plugging Eqs (5) and (6) into Eq (3), we find

$$\begin{array}{c} \begin{array}{cc} Y & =\frac{c(\alpha -1)S}{\alpha -\beta }(\frac{a^{\beta }b^{\alpha }S^{\delta \beta +\theta \alpha }-a^{\alpha }b^{\beta }S^{\theta \beta +\delta \alpha }}{ab^{\alpha }S^{\delta +\theta \alpha }-a^{\alpha }bS^{\theta +\delta \alpha }})\;, \end{array} \end{array}$$ (7)

that in the limit of *S* ≫ 1 yields

$$\begin{array}{c} Y=Y_{0}S^{\gamma }\;, \end{array}$$ (8)

where *Y*_0_ is a constant and *γ* = *γ*(*α*, *β*, *δ*, *θ*) is the country scaling exponent for total urban GDP. As detailed in S1 Appendix, Sec. 1, the exact form of *γ* depends on conditions imposed on the exponents *α*, *β*, *δ*, and *θ* (cases A.1-A.8 in S1 Appendix) which in turn emerge from determining the dominant term of Eq (7) in the limit of *S* ≫ 1. Therefore, the combination of Zipf’s law (Eq 1) with the urban scaling (Eq 2) and the country scaling relations of *s*_min_ and *s*_max_ (Eqs 5 and 6) leads us to the country scaling of total urban GDP (Eq 8), where the exponent *γ* depends on *α*, *β*, *δ* and *θ*.

The country scaling relations of GDP and *s*_max_ have previously been empirically observed in Refs. [42–44]. We have also verified that Eqs (5), (6) and (8) hold well for the country-aggregated values in our data set with *γ* > 1 and *δ* < *θ* (Fig 6 in S1 Appendix). More importantly, while *α* and *β* are intra-country exponents having different values for each country, *γ*, *δ* and *θ* are inter-country exponents and there is only one value for each across all countries. Thus, we can solve *γ* = *γ*(*α*, *β*, *δ*, *θ*) for *β* (S1 Appendix, Sec. 1 for details) to find

$$\begin{array}{c} \beta =\{\begin{array}{cc} 1+\frac{\gamma -1}{\theta } & 0<\alpha \leq 1\\ \frac{\gamma +\delta -1}{\theta }+(1-\frac{\delta }{\theta })\alpha & 1<\alpha <1+\frac{\gamma -1}{\delta }\\ 1+\frac{\gamma -1}{\delta } & \alpha \geq 1+\frac{\gamma -1}{\delta } \end{array}\;, \end{array}$$ (9)

for *γ* > 1 and *δ* < *θ*. This piecewise relationship implies that *β* is a constant up to *α* = 1, where it starts to increase linearly until reaching the diagonal line given by *β* = *α*, and then continues as another constant. It is worth mentioning that by writing *β* as a function of *α*, we are not assuming any causal direction for the association between both exponents. Indeed, we could also solve *γ* = *γ*(*α*, *β*, *δ*, *θ*) for *α* but this yields a non-functional dependency, that is, the horizontal plateaus defined by Eq (9) become vertical lines when writing *α* as function of *β*.

We have adjusted Eq (9) to the empirical relation between *α* and *β* (see Materials and methods) and the best fitting parameters are *γ* = 1.06±0.01, *δ* = 0.26±0.05, and *θ* = 0.79±0.03. The solid line in Fig 1A represents the best fit of Eq (9) and the colored background indicates the different ranges of *α* defined in Eq (9). Despite the large variations in the data, our model captures the average tendency of the empirical relation between *β* and *α*. We have verified that the statistical correlations between the values of *β* and *α* are only statistically significant in the mid-range of *α* values, while there are no significant correlations within the lower and higher ranges of *α* values (Fig 5 in S1 Appendix). Furthermore, the Akaike and Bayesian information criteria indicate that it is at least 100 times more likely that the empirical data come from our model (Eq 9) than from a null-model assuming no relationship between both exponents (intercept-only model: *β* = constant, see Fig 4 in S1 Appendix). We also find that the estimated parameters from Eq (9) are quite robust against thresholds for the total GDP; the adjusted values of *γ*, *δ*, and *δ* barely change, even if we only consider the countries with top 50% GDP values (Fig 7 in S1 Appendix).

In addition to the previous model validation, the adjustment of Eq (9) allows us to verify the consistency of our modeling approach through the country scaling relationships. Specifically, the country scaling exponents estimated from Eq (9) should describe well the empirical country scaling relationships. To do so, we have adjusted the country scaling relationships of Eqs (5), (6) and (8) by considering only the prefactors (*a*, *b*, and *Y*_0_) as fitting parameters and fixing country scaling exponents (*δ*, *θ* and *γ*) to the values estimated from Eq (9). Fig 2A–2C show that the three country scaling relations describe quite well the empirical data. Furthermore, the country scaling exponents obtained by fitting Eq (9) to the empirical relationship between *β* and *α* agree well with the estimates directly obtained from the country scaling relations, that is, by fitting Eqs (5), (6) and (8) to the country-aggregated data, as shown in Fig 2D. The values of *δ* and *θ* estimated from both approaches are not significantly different, while the values of *γ* are both above one but fitting the country scaling directly from data yields a slightly larger value. We believe this result provides support for our model, especially when considering the level of observed variation in the relationship between *β* and *α* as well as that in the country scaling relations.

### Simulating the association between both exponents

The level of variation in the relation between the exponents *β* and *α* (as well as in the country scaling relationships) is significantly high and hampers a more visual comparison with our model. In addition to indicating that our description of the association between both exponents is far from perfect, these variations also reflect possible estimation errors in the population and GDP values, errors related to the definition of city boundaries, and mainly errors associated with estimating the Zipf and urban scaling exponents (as data does not perfectly follow the Zipf’s and urban scaling laws). As these errors emerge from different sources and do not seem to affect the association between *β* and *α* systematically, we have treated them as random variations and investigated their role via an *in silico* experiment. As summarized in the Materials and Methods, we have generated artificial data at the city level (city population *s* and city GDP *y* values) by considering Zipf’s law (Eq 1), urban scaling (Eq 2), country scaling relations (Eqs 5 and 6), and our model (Eq 9). These simulations take as inputs the real values of total urban population *S* and the exponent *α* of each country to generate replicas of these urban systems (set of values of *s* and *y*), from which we can investigate the relationship between *β* and *α* under different levels of variation in the urban and country scaling relations. To do so, we have fixed the variation intensity (that is, the standard deviation of Gaussian random variation around the scaling laws) in the simulated country scaling relations to a level comparable with the empirical data, and varied the variation intensity in the simulated urban scaling *σ*_*y*_ in percentages of the value observed in the real data (*σ*_*y*_ = 100% means the standard deviation of the simulation is equal to the standard deviation in the empirical values of *β*).

The first four panels of Fig 3A show examples of simulated relationships (red circles) in comparison with empirical data (blue dots) for different values of *σ*_*y*_. As expected, the simulated relationship perfectly agrees with our model (Eq 9) when there is no random variation in the urban scaling. More importantly, we note that the scattering of simulated data becomes visually very similar to the empirical data as the intensity of the random variation increases up to *σ*_*y*_ = 100% (Fig 8 in S1 Appendix). We can also use the simulated data to corroborate our numerical experiment by verifying the country scaling relations. Fig 3B–3D show the simulated country scaling relations in comparison with the behavior of Eqs (5), (6) and (8) with parameters estimated from real data. We note that simulated scaling relations for total urban GDP (*Y*) and smallest city population (*s*_min_) follow very closely the adjusted behavior of the empirical data.

However, the simulated results for the largest city population (*s*_max_) systematically underestimate the trend observed in the empirical data. This happens because, in our simulations, we have used a random number generator associated with a truncated power-law distribution (between *s*_min_ and *s*_max_) for mimicking population values according to Zipf’s law. Since large populations are rare and the number of simulated cities is finite, the simulated values for the largest city population do not get close enough to the upper bound imposed by the truncated power-law distribution. Consequently, the simulated values of *s*_max_ underestimate the empirical ones. We solve this issue by replacing the truncated power-law behavior by a power-law distribution with exponential cutoff (Materials and Methods). This modification does not alter the country scaling relations of total urban GDP and smallest city population, that is, the results of Fig 3B and 3C are not affected by the introduction of the exponential cutoff. Similarly, this modification does not affect the relationship between *β* and *α* and the simulated associations with exponential cutoff are very similar to those obtained with the truncated power-law (as indicated by the right-most plot in Fig 3A). Indeed, the inclusion of this exponential cutoff only modifies the country scaling relation of the largest city population by increasing the simulated values of *s*_max_. Fig 3E shows an example of simulated results after after replacing the truncated cut-off with a exponential one for the country scaling relation between *s*_max_ and *S*. We observe that the simulated values of *s*_max_ obtained with the exponential cutoff are closer to the empirical data behavior than those obtained with the truncated power-law distribution (Fig 3D).

## Discussion

We have shown that the combination of Zipf’s law and urban scaling implies a country scaling relationship, where the exponent is a function of the Zipf and the urban scaling exponents. While the Zipf and the urban scaling exponents vary from country to country, there is only one country scaling exponent for a given indicator, which in turn implies a direct association between the urban scaling and the Zipf exponents. In qualitative terms, our results agree with the more holistic idea that urban scaling exponents do not solely emerge from processes occurring within the city boundaries; instead, cities do not represent a closed and non-interacting system and what happens in the entire system (such as flow of people and goods) may affect urban scaling. Similarly to what happens in transportation theory where the product of two cities’ populations is usually assumed to be proportional to the commuting flow between them (such as in gravity models), the population of cities is likely to represent an important factor for the interactions among cities. Under this assumption, the distribution of city population (summarized by the Zipf exponent) may thus represent an indirect proxy for interactions between cities, and the association observed between *β* and *α* summarizes how the population distribution affects urban scaling and vice versa.

Theoretically, the connection between the exponents *β* and *α* would imply that instead of unrelated, Zipf’s law and urban scaling are indeed the two sides of the same coin. However, the non-negligible variability observed in the empirical relationship do not corroborate with such a simple conclusion but suggest that other factors (such as level of socio-economic development and the particular history of an urban system) beyond population distribution may also have a significant effect on the urban scaling of GDP. Understanding the relative importance of population distribution on urban scaling (and vice versa) for different indicators is an important future contribution that is currently limited by the availability of other world-wide comparable city indicators. Even when considering the unexplained variation in the data, the connection between the two scaling laws uncovered by our work indicates the existence of universal processes governing both laws; however, finding out this commonality for arbitrary urban indicators still represents a challenging task.

In the context of urban GDP, our results show that urban systems with small values of the Zipf exponent also tend to present lower increasing returns to scale of GDP (low values of *β*). An urban system described by a small Zipf exponent has a more balanced population distribution, and consequently, fewer small cities and more large cities when compared with urban systems described by larger Zipf exponents. Thus, countries with proportionally more metropolises tend to have less pronounced increasing returns to scale than those having a small number of large cities. We hypothesize that urban systems with a large number of metropolises may also have a more integrated market whereby these large cities cooperate and develop specialized economic activities. As a result, urban systems with more metropolises would have a smaller degree of agglomeration of economic activities in large cities and so weaker increasing returns to scale for city GDP. In contrast, countries with a small number of large cities have to concentrate almost all complex economic activities in relatively fewer metropolises, which in turn intensifies the increasing returns to scale of urban GDP (high values of *β*).

It is also interesting to note that high-GDP countries present relatively smaller values of Zipf and urban scaling exponents than mid-GDP and low-GDP countries (Fig 1A and Fig 9 in S1 Appendix), that is, developed countries tend to have more metropolises and less pronounced increasing returns to scale of urban GDP. This latter point agrees with the more local observation that “rich cities” of the European Union (West cities) also exhibit smaller scaling exponents for GDP than their “poor” counterparts (East cities) [27]. Large values of *β* may express an urban system with high economic performance, but because *β* alone does not define the total urban GDP, large values of *β* also indicate a significant imbalance between the economic productivity of small and large cities. The association between *β* and *α* also suggests that part of this economic inequality may reflect the unbalanced distribution of population. It is also worth mentioning the possibility of the existence of different urban planning regimes [45] that may prevent sharp population agglomerations in developed countries and thus also partially explain the negative association between *β* and total urban GDP.

Our data do not allow a dynamic analysis nor the identification of the causal direction of the association between the exponents *β* and *α*. Still, a possible explanation for the observed differences among countries with different levels of development is that the economic policies in less developed countries have focused on large cities, fostering this unbalanced situation and creating cities larger than an hypothetical economically optimal. From a labor-market perspective, these large cities may attract inhabitants from smaller cities, changing both the urban scaling and the Zipf exponents. However, these new inhabitants may mostly find low-paying jobs or even become unemployed, which in turn might partially explain the poorer overall economic performance of less efficient urban systems. The association between both exponents is even more crucial because the world urban population may increase up to 90% by 2100 [46]. This urbanization process is likely to be even more intense in developing countries and has the potential to further undermine their economic performance. Thus, it is not only important to discuss which scaling is desirable but also the population distribution within urban systems.

## Materials and methods

### Data

The data used in this work is a product of two data sets. First, we use the recently released GHSL-FUA or eFUA [38, 39] definition of functional urban areas (FUAs). The eFUAs uses gridded population data from Global Human Settlement Layer (GHSL) [47, 48] and an automated classification approach for producing 9,031 urban boundaries (and population counts) over the entire globe (188 countries) for the year 2015. The eFUAs comprise high-density urban centers and their surrounding commuting zones and aim to capture the functional extent of cities. Second, we use the gridded GDP data provided by Kummu *et al*. [40]. This data set combines sub-national and national GDP data from different sources with population gridded data (from the GHSL and the HYDE 3.2 [49]) to define three gridded global datasets: Gross Domestic Production per capita (5 arc-min resolution), Human Development Index (5 arc-min resolution), and Gross Domestic Production (30 arc-sec-min resolution). We have used only this latter file representing gridded values of total GDP with a resolution of 30 arc-sec (1 km at equator) for the year 2015 (the same information is also available for the years 1990 and 2000). To define GDP consistently at the grid level across countries, Kummu *et al*. first calculate the GDP across gridcells within each subnational unit of a given country as the corresponding subnational GDP per capita value multiplied by the gridcell population. Next, the authors sum over these gridcell GDP values and divide by the sum gridcell populations in a country to define the population-weighted national GDP per capita. They then calculate the ratio between this population-weighted national GDP per capita and the subnational GDP per capita. Finally, they multiply this ratio by the national GDP per capita to obtain the final subnational GDP per capita values (that vary across subnational units in each country), which they then multiply by the population in each gridcell within each subnational unit to obtain the final GDP for every gridcell in a given country. This method ensures that the sum over GDP per capita values at the gridcell level always coincides with officially reported GDP per capita values for each country, and that there is global consistency because the method relies on secondary sources of reported subnational GDP per capita and internationally consistent population grids. The gridded GDP values are reported in 2011 international US dollars using purchasing power parity rates (total GDP-PPP). We overlay the gridded GDP data with eFUA boundary polygons and aggregate the GDP cell values within each polygon for associating a GDP value to each eFUA. We illustrate this procedure in Fig 1 in S1 Appendix. Next, we group the resulting data by country and select the countries having at least 10 eFUAs. We also removed from our analysis 46 eFUA with null GDP (16 from India; 9 from Ethiopia; 5 from Pakistan; 3 from Sudan; 2 from Niger, Congo, and Chad; and 1 from Argentina, Benin, Egypt, Indonesia, Myanmar, Senegal, and Uganda). These criteria lead us to a data set comprising population *s* and GDP *y* of 8,650 functional urban areas from 96 countries.

### Estimating the Zipf exponent

Zipf’s law (Eq 1) implies that the complementary cumulative distribution function (CDF) of city population is a power law, *F*(*s*)∼*s*^−*ζ*^, with *ζ* ≈ *α*. We use this connection to estimate the values of *α* from the data. Specifically, we applied the approach of Clauset-Shalizi-Newman [50] to obtain the exponent *α* via its maximum likelihood estimate $\alpha =n/(\sum_{i=1}^{n}\ln s_{i}/{\tilde{s}}_{\text{min}})\;,$ where ${\tilde{s}}_{\text{min}}$ is the lower bound of the power-law regime, *s*_*i*_ is the population of the *i*-th city for a given country such that $s_{i}\geq {\tilde{s}}_{\text{min}}$, and *n* is the number of city populations in the power-law regime. The value of ${\tilde{s}}_{\text{min}}$ is also estimated from data by minimizing the Kolmogorov-Smirnov “distance” between the empirical CDF of city populations and *F*(*s*). The standard error in the Zipf exponent ${\text{SE}}_{\alpha }=\alpha /\sqrt{n}$ can be obtained from the width of the likelihood maximum [50]. We have used the Clauset-Shalizi-Newman method as implemented in the Python module powerlaw [51]. In addition to being a quite popular approach for fitting power-law distributions, Bhattacharya *et al*. [52] have recently proven that the Clauset-Shalizi-Newman approach yields an unbiased and consistent estimator, that is, as data increases indefinitely the estimated parameters converge (in distribution) to the true values. We show the CDF and Zipf’s law adjusted to each country in S1 Appendix, Sec. 4, where a good agreement is observed in the vast majority of cases. After estimating ${\tilde{s}}_{\text{min}}$, we filter out all cities with population smaller than this threshold in all other analyses, leading us to 4,571 functional urban areas from 96 countries. Thus, the urban scaling laws involve only cities belonging to the power-laws regime, and the countries’ urban GDP (*Y*) and countries’ urban population (*S*) are the aggregated values of urban GDP (*y*_*i*_) and urban population (*s*_*i*_) of cities belonging to the power-law regime for each country.

### Estimating the urban scaling and the country scaling exponents

Urban scaling and country scaling laws are generically represented by a power-law relation between a dependent variable *z* and an independent variable *x*

$$\begin{array}{c} z=g\;x^{\nu }\;, \end{array}$$ (10)

where *g* is a prefactor and *ν* is the power-law exponent. Eq (10) is linearized via a logarithmic transformation

$$\begin{array}{c} \log z=\log g+\nu \log x\;, \end{array}$$ (11)

where log*z* and log*x* now represent the dependent and independent variables, log*g* is the intercept and *ν* the slope, both being regression coefficients of a corresponding linear model. We have estimated the values of log*g* and *ν* by adjusting Eq (11) to the log-transformed data via robust linear regression with the Huber loss function [53], as implemented in the Python module statsmodels [54]. We further estimate standard errors and confidence intervals for the parameters log*g* and *ν* via bootstrapping [55]. We show the urban scaling law adjusted to each country in S1 Appendix, Sec. 4, where a good agreement is observed in the vast majority of cases. The adjusted country scaling laws are shown in Fig 6 in S1 Appendix. In the case of Fig 2A–2C, we have fixed the power-law exponents (regression coefficients) *γ*, *δ* and *θ* to values obtained from fitting Eq (9) to the relation *β* versus *α*, and only the prefactors (intercepts of the linear model) log*Y*_0_, log*a*, and log*b* have been considered as free parameters in the regression model.

### Fitting our model to the relationship between *β* and *α*

Our model for the relationship between *β* and *α* is completely defined in S1 Appendix, Sec. 2. Depending on whether *γ* > 1 or *γ* < 1 and also on whether *δ* < *θ* or *δ* > *θ*, we have an expression of *β* as function of *α* (Eqs. S56-S59, in S1 Appendix) and Eq (9) is a particular case when *γ* > 1 and *δ* < *θ*. We have adjusted the complete model (that is, without assuming anything about the parameters *γ*, *δ*, and *θ*) to the empirical relation between *α* and *β* via the L-BFGS-B nonlinear optimization algorithm [56], as implemented in the Python module lmfit [57] and without any constraint. The standard errors and the confidence intervals on the parameters *γ*, *δ* and *θ* are estimated via bootstrapping [55]. The best fitting parameters (± standard errors) are *γ* = 1.06±0.01, *δ* = 0.26±0.05, and *θ* = 0.79±0.03. This leads to Eq (9) because the best fitting parameters yield *γ* > 1 and *δ* < *θ*. Figs 10 and 11 in S1 Appendix depict different versions of Fig 1A where we label all countries and show the standard errors in *β* and *α*.

### Simulating relations between *β* and *α*

We simulate the relationship between *β* and *α* by generating data at the city level. For a given country population *P* with Zipf exponent *α*, we start by generating a list of *m* city populations $S=\{s_{1},\ldots ,s_{i},\ldots ,s_{m}\}$ until satisfying the constraint $\sum_{i=1}^{m}s_{i}\approx S\;10^{N(0,\sigma_{\gamma })}$, where $N(0,\sigma_{\gamma })$ is a Gaussian random variable with zero mean and standard deviation *σ*_*γ*_. Each *s*_*i*_ is drawn from a power-law distribution *p*(*s*)∼*s*^−(*α*+1)^ (compatible with Zipf’s law) within the interval *s*_min_ < *s* < *s*_max_, where *s*_min_ and *s*_max_ are obtained from the country scaling relations (Eqs 5 and 6) with multiplicative random variations, that is, $s_{\text{min}}∼S^{\delta }\;10^{N(0,\sigma_{\delta })}$ and $s_{\text{min}}∼S^{\theta }\;10^{N(0,\sigma_{\theta })}$, where $N(0,\sigma_{\delta })$ and $N(0,\sigma_{\theta })$ are Gaussian random variables with zero mean and standard deviations *σ*_*δ*_ and *σ*_*θ*_, respectively. We next generate a list of urban indicators $Y=\{y_{1},\ldots ,y_{i},\ldots ,y_{m}\}$, where $y_{i}=c\;s_{i}^{\beta }\;10^{N(0,\sigma_{y})}$ and $N(0,\sigma_{y})$ is a Gaussian random variable with zero mean and standard deviation *σ*_*y*_. In the expression for *y*_*i*_, the value of *β* is obtained from our model (Eq 9) while the value of *c* is chosen to satisfy the condition $\sum_{i=1}^{m}y_{i}\approx Y$, where *Y* is the total urban GDP (S1 Appendix, Sec. 3). The random variation controlled by the parameters *σ*_*γ*_, *σ*_*δ*_, and *σ*_*θ*_ mimics the variability observed in the empirical country scaling relationships, and we set their values equal to the standard deviation of the bootstrap estimates of the country scaling exponents (*γ*, *δ*, and *θ*). On the other hand, the random variation controlled by *σ*_*y*_ mimics the variability in the urban scaling relationships, and we set its value as a fraction of the standard deviation of the empirical values of *β*. We have thus applied this procedure by using all empirical values of *α* and *Y* to obtain the simulated values of *β*, *Y*, *s*_min_, *s*_max_ from the lists S and Y under different values of *σ*_*y*_ (Fig 3A).

We have also considered a modification of this procedure where the population values were drawn from a power-law distribution with exponential cutoff [50], that is, *p*(*s*)∼*s*^−(*α*+1)^exp(−*s*/*s*_0_) (*s* > *s*_min_), where *s*_0_ is an additional parameter. This modification is necessary for reproducing the empirical behavior of the country scaling between *s*_max_ and *S*. Because large city populations are very rare, the simulated values of *s*_*i*_ obtained from the upper-truncated power-law distribution do not get close enough to the imposed maximum value (*s*_max_). This results in the underestimation of *s*_max_, as shown in Fig 3D. After replacing the upper-truncated behavior by the exponential cutoff, we note that the simulated country scaling of *s*_max_ becomes very similar to the empirical relation (Fig 3E). In this simulation, we have chosen the value *s*_0_ = 3 × 10^7^ to make the simulated values of *s*_max_ closer to the scaling law adjusted from the empirical data. It is worth mentioning that this change has no effect on the relationship between *β* and *α* nor on the other country scaling relations (see the two right-most plots of Fig 3A).

S1 Appendix, Sec. 3 shows more details on how we have implemented this simulation.

## Supporting information

S1 Appendix (PDF)
