# Supplementary material for: Association between population distribution and urban GDP scaling
Source: PLoS One. 2021 Jan 22;16(1):e0245771. doi: 10.1371/journal.pone.0245771 (PMC7822261; doi:10.1371/journal.pone.0245771)
Supplement: S1 Appendix — (PDF) [file pone.0245771.s001.pdf]

S1 Appendix for

Association between population distribution and urban GDP scaling

H. V. Ribeiro*, M. Oehlers, A. I. Moreno-Monroy, J. P. Kropp, D. Rybski*

*Corresponding authors. E-mail: hvr@dfi.uem.br or ca-dr@rybski.de

Supplementary Figures

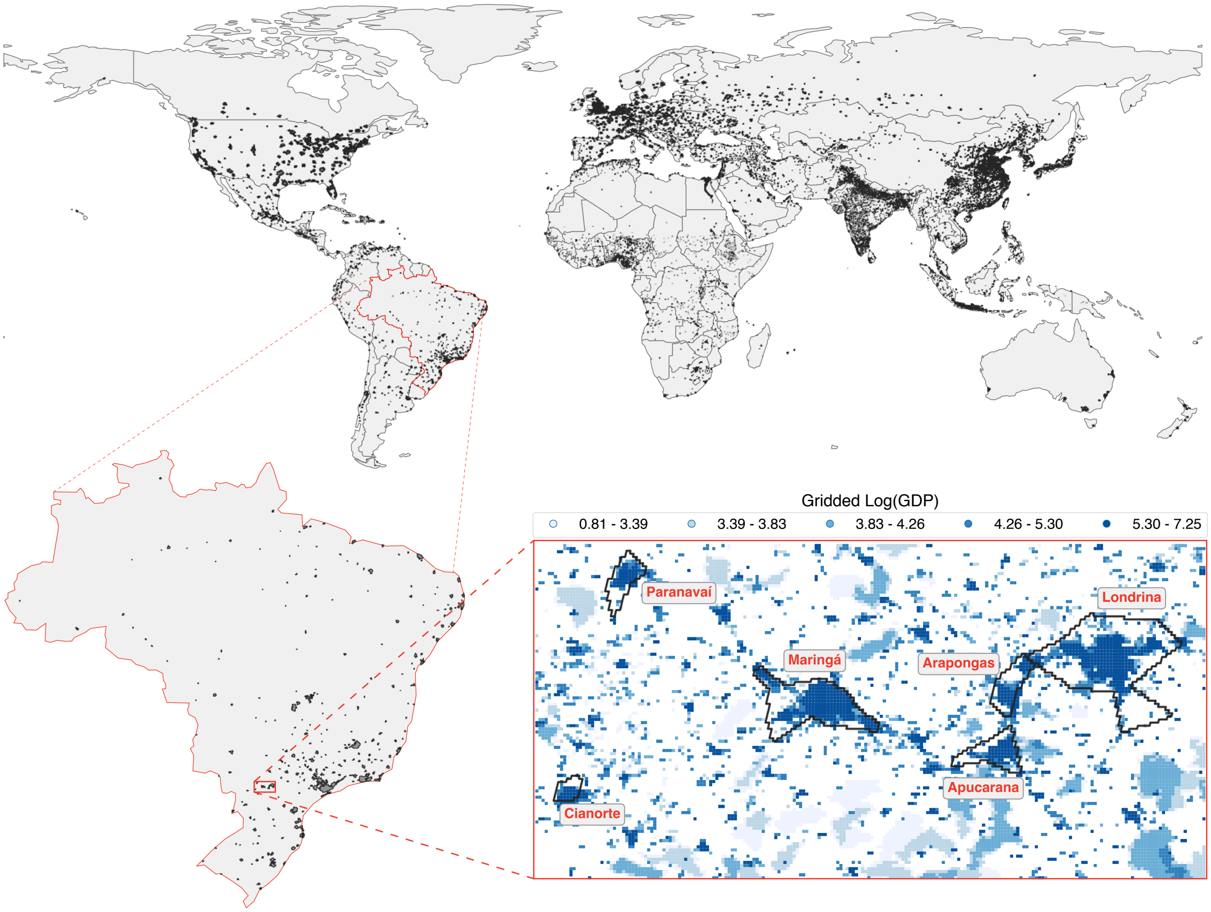

FIG. 1. **Illustration of the procedure used for obtaining population s and GDP y of cities over the entire globe.** The world map shows the boundaries of GHSL-OECD Functional Urban Areas (GHS-FUA or eFUA). This definition of urban areas has been recently released by the European Commission's science and knowledge service, with the aim of providing an internationally-comparable and harmonized definition of urban areas. The eFUA definition does not rely on local administrative boundary units and commuting flows data; instead, it uses gridded data from the Global Human Settlement Layer (GHSL) and an automated classification approach for producing urban boundaries [JRC Tech. Rep. (2019) and J. Urban. Econ., 103242 (2020)]. The shape file is provided for the year of 2015 together with population values for each eFUA. We combine eFUA boundary data with gridded GDP data provided by Kummu et al. [Sci. Data 5, 180004 (2018)]. In more detail, we use the total GDP-PPP (purchasing power parity, in constant 2011 international USD) data with 30 arc-sec resolution (≈1 km at the equator, file name: `GDP_PPP_30arcsec_v2.nc`) for the year 2015 to overlay with eFUA boundaries. Next, we aggregate all grid cell values within each eFUA boundary to associate a GDP value to each urban area of the world. We illustrate this idea by highlighting a small area in the southern part of Brazil, where we also plot the GDP grid cells.

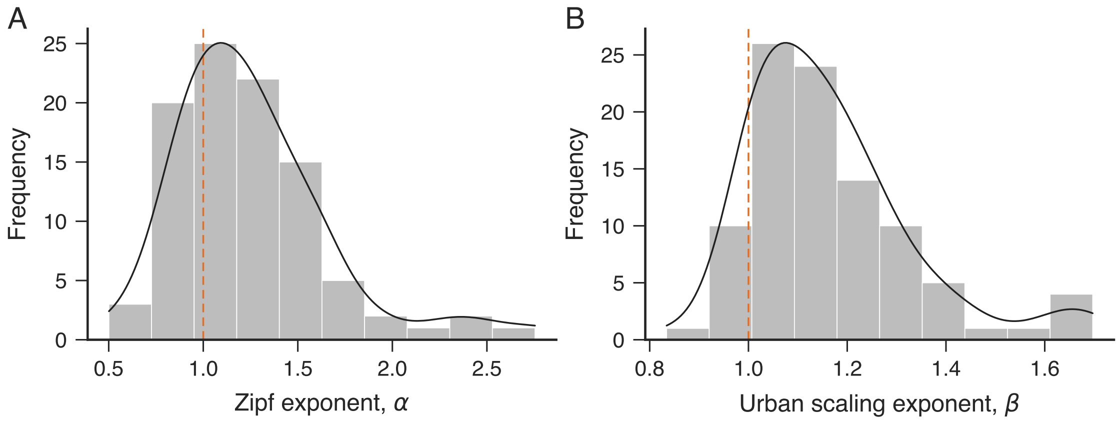

FIG. 2. **Probability distributions of the Zipf exponent α and the urban scaling exponent β.** (A) The bars represent a histogram for the values of α and the continuous line is a kernel density estimation (not normalized) for the same data. The vertical dashed line indicates the value $\alpha = 1$. The average value and the standard deviation of α are 1.24 and 0.38, respectively. (B) The bars represent a normalized histogram for the values of β and the continuous line is a kernel density estimation (not normalized) for the same data. The vertical dashed line indicates the value $\beta = 1$. The average value and the standard deviation of β are 1.16 and 0.17, respectively.

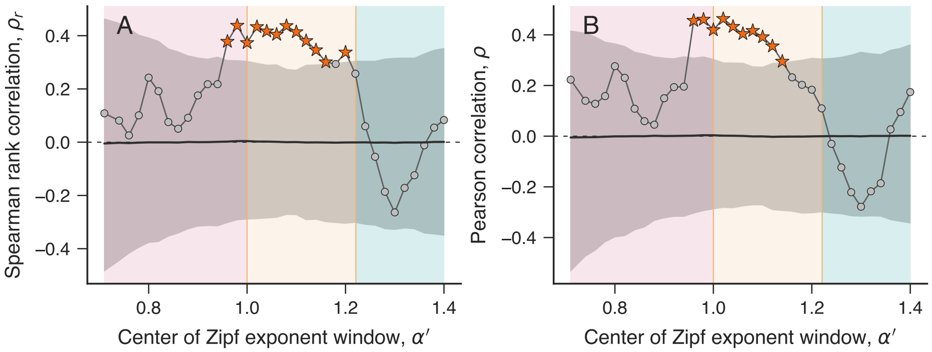

FIG. 3. **Correlation between the urban scaling exponent β and the Zipf exponent α over sliding windows of values of α.** (A) Spearman rank correlation (ρ_r) of the relationship between β and α estimated within a sliding window centered in α'. (B) Pearson correlation (ρ) of the relationship between β and α estimated within a sliding window centered in α'. For both correlation measures, the size of sliding windows is $\Delta\alpha' = 0.4$ and the step is 0.02. In addition, the red star markers indicate the correlation values that statistically significant at the 95% confidence level (p-values smaller than 0.05), while the gray circles represent non-significant correlation values (p-values larger than 0.05). Similar results are obtained for $\Delta\alpha'$ between 0.15 and 0.55. In both panels, the continuous black lines around zero represent average values of the correlation measures estimated after shuffling the values of β (1000 realizations), and the gray shaded region stands for the 95% bootstrap confidence intervals. We notice that the correlation measures are larger and statistically significant for values α' around 1.1 and that the significant values cannot be explained by the distribution of β values. This range containing the largest and significant values of ρ and ρ_r mostly correspond with mid-range of α values in Eq. (9) [Eq. S56 in Supplementary Text], where our model predicts a linear association between β and α (see main text for details).

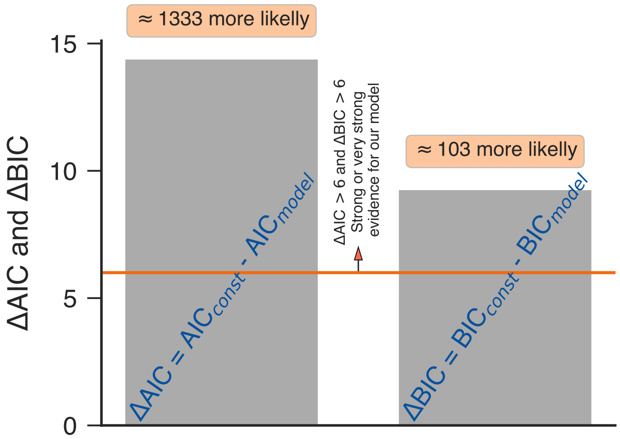

FIG. 4. **Comparison between the model of Eq. (9) [Eq. S56 in Supplementary Text] and a constant (intercept-only) model (β = const).** The left bar shows the difference between the AIC (Akaike information criterion) values (ΔAIC) estimated for an intercept-only model ($\text{AIC}_{\text{const}}$) and for the model of Eq. (9) [Eq. S56 in Supplementary Text] ($\text{AIC}_{\text{model}}$). The model with the lowest AIC value gives a better description of the data. Thus, $\Delta\text{AIC} > 0$ indicates that the model of Eq. (9) [Eq. S56 in Supplementary Text] is a better description for data. As a rule of thumb, it is common to consider $6 < \Delta\text{AIC} < 10$ as a strong evidence and $\Delta\text{AIC} \geq 10$ as a very strong evidence (our case, since $\Delta\text{AIC} \approx 14.4$) in favor of the model of Eq. (9) [Eq. S56 in Supplementary Text]. In addition, because the AIC value is proportional to the negative of log-maximum-likelihood of the model, the quantity $e^{\Delta\text{AIC}}$ approximates how more likely the model of Eq. (9) [Eq. S56 in Supplementary Text] is to describe the data when compared with an intercept-only model. Thus, we notice that Eq. (S56) is ≈ 1333 times more likely to describe the relationship between β and α than the intercept-only model. The right bar shows the difference between the BIC (Bayesian information criterion) values (ΔBIC) estimated for an intercept-only model ($\text{BIC}_{\text{const}}$) and for the model of Eq. (9) [Eq. S56 in Supplementary Text] ($\text{BIC}_{\text{model}}$). The interpretation of BIC and AIC values are analogous. The results indicate a strong evidence ($\Delta\text{BIC} \approx 9.3$) in favor of Eq. (9) [Eq. S56 in Supplementary Text] and our model is ≈ 103 times more likely to describe the empirical data than the intercept-only model. It is worth remembering that both AIC and BIC values account for the fact that Eq. (9) [Eq. S56 in Supplementary Text] has more free parameters than the intercept-only model (3 versus 1 parameter); moreover, BIC values penalize the number of parameters more heavily.

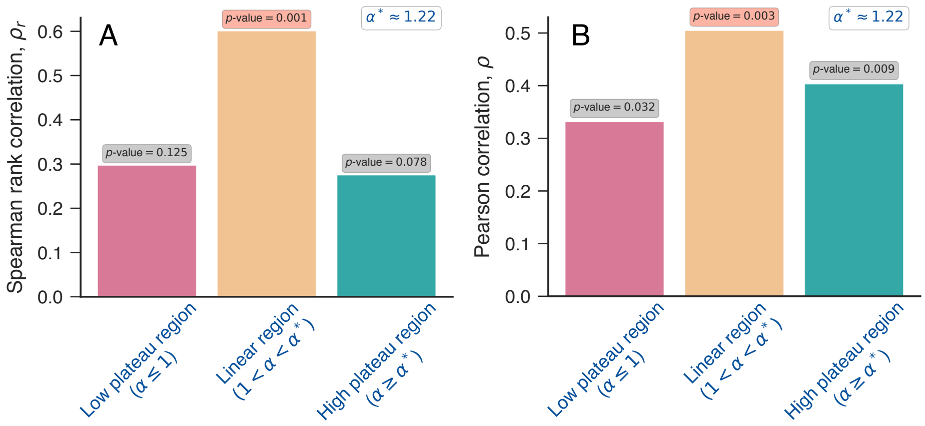

FIG. 5. **Correlations in each range of values of α defined in Eq. (9) [Eq. S56 in Supplementary Text]**. (A) Spearman rank correlation ρ_r between β and α in the low plateau ($\alpha \le 1$), linear regime ($1 < \alpha < \alpha^*$), and high plateau ($\alpha \ge \alpha^*$) regions of Fig. 1 in the main text. Panel (B) shows the same for the Pearson correlation coefficient ρ. In both panels, we indicate above the bars the p-values testing the significance of the correlation coefficient (permutation test). We notice that ρ_r and ρ are significantly higher in the linear region. We further observe that the correlations are statistically significant at 99% confidence level only in the linear region. Here $\alpha^* = 1 + \frac{\gamma-1}{\delta} \approx 1.22$ [Eq. (9) of the main text or Eq. S56 in Supplementary Text, and Fig. 15].

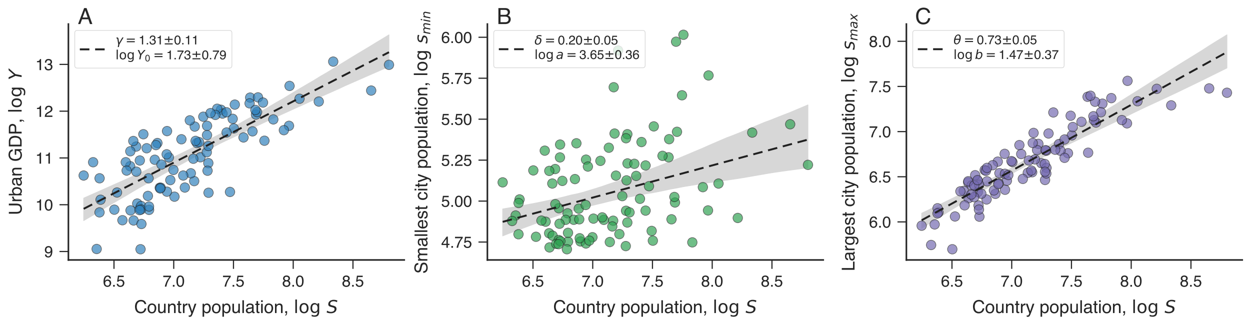

FIG. 6. **Fitting country scaling relationships directly to data.** (A) Scaling relation between total urban GDP of countries (Y) and country's urban population values (S). (B) Scaling law between the smallest city population of a country ($s_{\min}$) and country's urban population (S). (C) Scaling law between the largest city population of a country ($s_{\max}$) and country's urban population (S). In the three panels markers represent the values for each country and the dashed lines are the country scaling relationships of Eqs. (8), (5), and (6) [Eqs. (S6), (S7), and (S8) in Supplementary Text], where the exponents γ, δ, and θ (and the prefactors $\log Y_0$, $\log a$, and $\log b$) are obtained via robust linear regression of the log-transformed data. The best fitting parameters are shown in the panels ($\pm$ standard errors) and gray shaded regions stand for the 95% confidence band of the models. As discussed in the main text, the exponents estimated directly from the scaling laws are very similar to the values obtained by fitting Eq. (9) [Eq. S56 in Supplementary Text] to the relationship between β and α (see Fig. 2D of main text for a comparison).

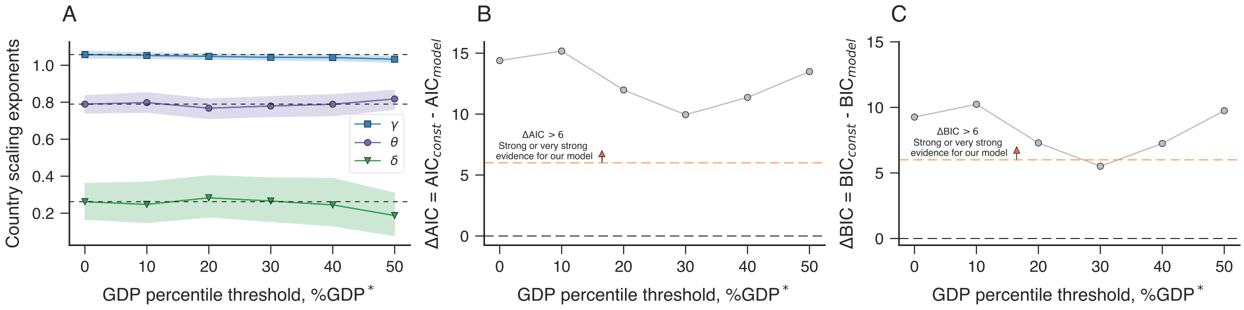

FIG. 7. **Robustness of the model of Eq. (9) [Eq. S56 in Supplementary Text] against different GDP threshold.** (A) The colored markers show the values of γ, δ, and θ estimated by fitting (via the L-BFGS algorithm) Eq. (9) [Eq. S56 in Supplementary Text] to the relationship between β and α after selecting countries with total urban GDP higher than the percentile GDP threshold (%GDP*). The shaded regions are 95% bootstratp confidence intervals and the dashed lines represent the values when considering all data. We notice the exponent estimates are quite stable. (B) Difference between the AIC (Akaike information criterion) values (ΔAIC) estimated for an intercept-only model ($\mathrm{AIC_{const}}$) and for the model of Eq. (9) [Eq. S56 in Supplementary Text] ($\mathrm{AIC_{model}}$) as a function of the percentile GDP threshold (%GDP*). (C) Difference between the BIC (Bayesian information criterion) values (ΔBIC) estimated for an intercept-only model ($\mathrm{BIC_{const}}$) and for the model of Eq. (9) [Eq. S56 in Supplementary Text] ($\mathrm{BIC_{model}}$) as a function of the percentile GDP threshold (%GDP*). Results show strong evidence (ΔAIC > 6 and ΔBIC > 6) for the model of Eq. (9) [Eq. S56 in Supplementary Text] at practically all threshold values.

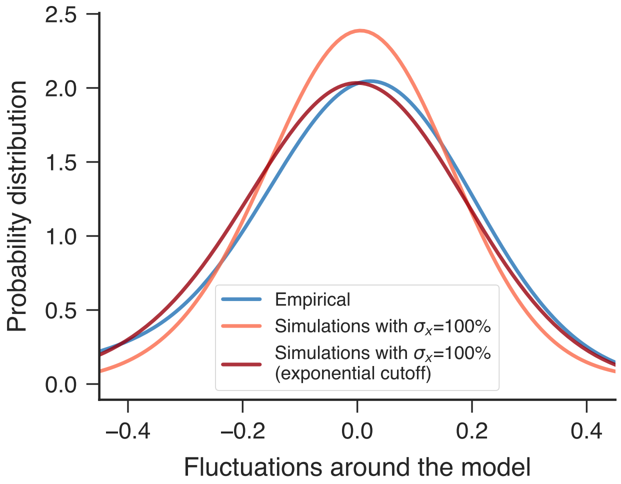

FIG. 8. **Probability distributions of the random variations around the model of Eq. (9) [Eq. S56 in Supplementary Text].** The different curves show kernel density estimations of the random variations around the model of Eq. (9) when considering the empirical data and the simulations obtained for $\sigma_y = 100\%$. We observe that the distributions obtained from the simulations are very similar to the one emerging from the empirical data, especially when including an exponential cutoff in the power-law distribution associated with the simulated city sizes.

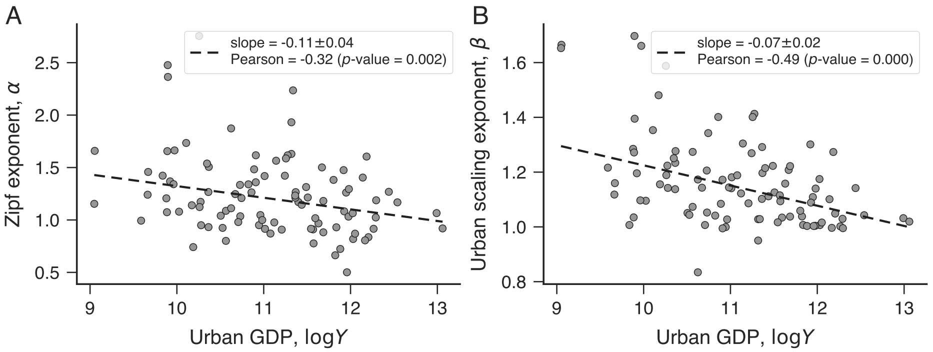

FIG. 9. **Association between the exponents α and β and total urban GDP Y.** (A) Scatter plot of Zipf exponent α versus the total urban GDP Y. (B) Scatter plot of urban scaling exponent β versus the total urban GDP Y. The dashed line in each panel represents a linear regression with slope indicated within the plots (where we also shoe the Pearson correlation coefficient and the corresponding p-values). We observe that both exponents also significantly associated with urban GDP Y, such that an increase in Y tends to be followed by a decrease in α and β.

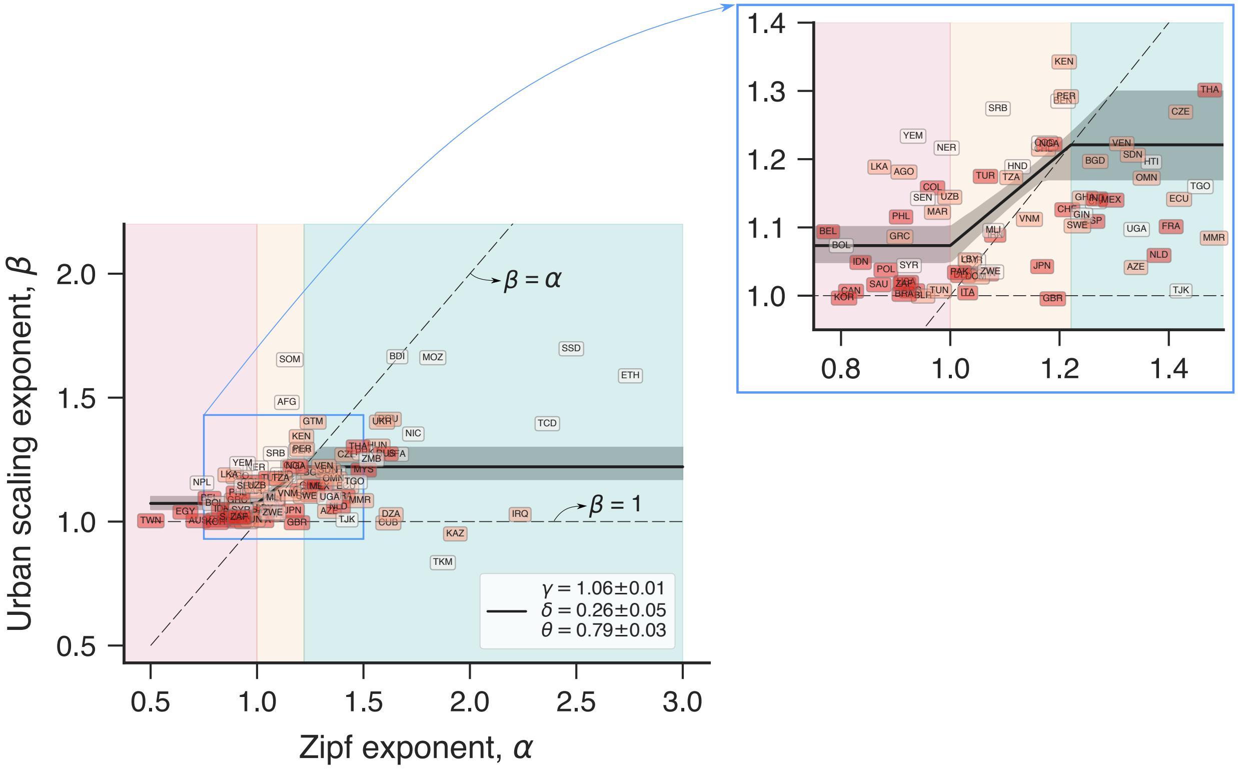

FIG. 10. **Association between the urban scaling exponent β and the Zipf exponent α.** The markers indicate the values of β versus α for each country in our data set. The three marker colors group countries according to the tercile values of the total urban GDP distribution (for instance, High-GDP countries have highest $\approx 33\%$ GDP values, as the main text). The horizontal dashed line shows the $\beta = 1$, while the inclined dashed line represents the $\beta = \alpha$ relationship. The continuous line shows the model of Eq. (9) [Eq. S56 in Supplementary Text] adjusted to the data and the gray shaded region stands for the 95% bootstrap confidence band. The colored shaded regions represent the different intervals of α defined in Eq. (9) [Eq. S56 in Supplementary Text]. All countries are labeled according to their ISO codes (the three letters over each marker). The blue frame indicates a region that has been magnified on the right.

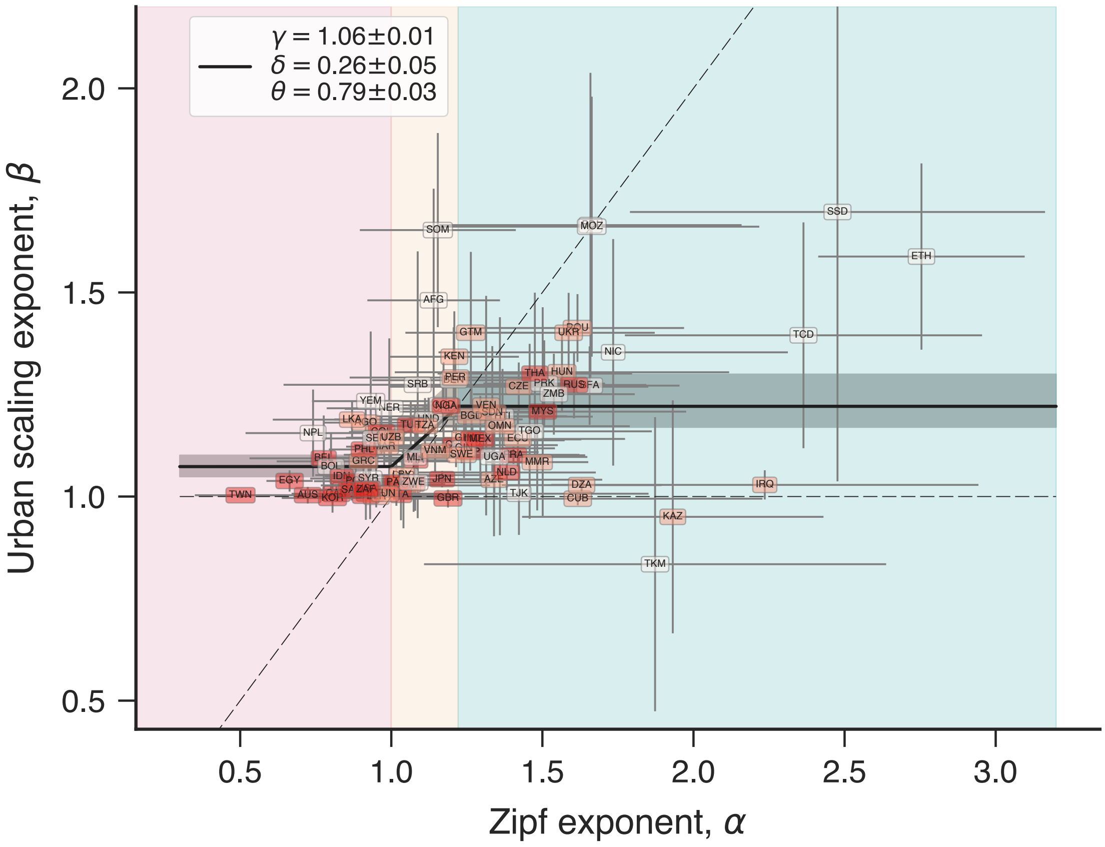

FIG. 11. **Association between the urban scaling exponent β and the Zipf exponent α.** The same as Fig. (10) but with error bars indicating standard errors. The three colors group countries according to the tercile values of the total urban GDP distribution (for instance, High-GDP countries have highest ≈ 33% GDP values). The horizontal dashed line shows the $\beta = 1$, while the inclined dashed line represents the $\beta = \alpha$ relationship. The continuous line shows the model of Eq. (9) [Eq. S56 in Supplementary Text] adjusted to data and the gray shaded region stands for the 95% bootstrap confidence band. The colored shaded regions represent the different intervals of α defined in Eq. (9) [Eq. S56 in Supplementary Text]. All countries are labeled according to their ISO codes (the three letters over each marker).

Supplementary Text

1. CONNECTION BETWEEN URBAN SCALING AND ZIPF EXPONENTS

Let us start by considering an urban system (a country) composed of m cities with populations $\{s_1, s_2, \ldots, s_m\}$. Suppose now that we sort these population values s in descending order $\{s_{\max}, \ldots, s, \ldots, s_{\min}\}$ (where $s_{\max}$ and $s_{\min}$ are respectively the largest and smallest city population in the system) and associate a rank variable r defined by $\{1, 2, \ldots, r, \ldots, m\}$ to each city population. Zipf's law for cities states that rank r and city population s are related via

$$r \sim s^{-\alpha}\,, \tag{S1}$$

where $\alpha > 0$ is a power-law exponent or the Zipf exponent. Zipf's law implies that the complementary cumulative distribution of city population $F(s)$ can be written as $F(s) \sim s^{-\zeta}$, with $\zeta \approx \alpha$, and, consequently, the probability density distribution $P(S)$ of city population is

$$P(s) \sim s^{-(\alpha+1)} \qquad \text{for } s_{\min} < s < s_{\max}\,. \tag{S2}$$

From Eq. (S2) we can also write the frequency function or the absolute number of cities with population s as

$$p(s) = ks^{-(\alpha+1)} \qquad \text{for } s_{\min} < s < s_{\max}\,, \tag{S3}$$

where k is a normalization constant determined by

$$S = \sum_{s=s_{\min}}^{s_{\max}} sp(s) \approx k \int_{s_{\min}}^{s_{\max}} s^{-\alpha} ds \tag{S4}$$

and S is the total population of the urban system.

On the other hand, the urban scaling hypothesis establishes a power-law relationship between an urban indicator y (*e.g.* the urban GDP) and the population s of a city, that is,

$$y = cs^{\beta}\,, \tag{S5}$$

where c is a positive constant, and $\beta > 0$ is the urban scaling exponent. Our work has shown that the values of β and α are related to each other, and

we propose that the relationship between these two exponents emerge from country scaling relationships.

To derive the relationship between β and α, we consider S as the total population of an urban system and Y as the total quantity of the city indicator y for the whole urban system (*e.g.* total urban GDP). It is worth noticing that the values of S and Y represent the empirical values of total population and total GDP of all cities of a country within the power-law regime (that is, all cities with population larger than the lower cutoff obtained after fitting the Zipf's law to the data, see Material and Methods in the main text for details). In addition to the urban scaling that considers relations between population and indicators of cities within one country, we further assume three different country scaling relations between population and indicators across countries. The first one is a generalization of the urban scaling hypothesis at the country level

$$Y = Y_0 S^{\gamma}\,, \tag{S6}$$

where Y_0 is a constant, and γ is the country scaling exponent associated with the indicator Y. The other two relationships relate the population of the smallest ($s_{\min}$) and largest ($s_{\max}$) cities in an urban system to the total population S, that is,

$$s_{\min} = aS^{\delta} \tag{S7}$$

$$s_{\max} = bS^{\theta}\,, \tag{S8}$$

where a and b are positive constants, and $\delta > 0$ and $\theta > 0$ are two additional country scaling exponents. We have empirically verified these three country scaling relationships in our data (Fig. 6 and Fig. 2 of the main text); however and as we shall prove, the relationship of Eq. (S6) can also be derived from Zipf's law (Eq. S3), urban scaling (Eq. S5) and the two country scalings for $s_{\min}$ and $s_{\max}$ (Eqs. S7 and S8). Qualitatively speaking, we consider that the existence of country scaling relationships constrains the population and urban indicator values in a country which in turn ties the values of β and α.

To find this connection, we notice that the total indicator Y can be ex-

pressed by using Zipf's law and urban scaling, that is,

$$Y = \sum_{s=s_{\rm min}}^{s_{\rm max}} y p(s) = \sum_{s=s_{\rm min}}^{s_{\rm max}} c s^{\beta} p(s) \approx kc \int_{s_{\rm min}}^{s_{\rm max}} s^{\beta-\alpha-1} ds\,. \tag{S9}$$

By determining the constant k from Eq. (S4) and solving the integral in Eq. (S9), we should obtain the country scaling of Eq. (S6) in terms of the exponents α and β. Thus, if we assume γ, δ, and θ to be fixed, the changes in α and β must be constrained in order to yield the particular value of γ imposed by country scaling in Eq. (S6).

Case A: $\alpha \neq \beta \neq 1$

To start our calculations, we first consider the general case where $\alpha \neq \beta \neq 1$. Under this assumption, Eq. (S4) yields

$$k = \frac{(\alpha-1) S s_{\rm max}^{\alpha} s_{\rm min}^{\alpha}}{s_{\rm max}^{\alpha} s_{\rm min} - s_{\rm max} s_{\rm min}^{\alpha}}\,, \tag{S10}$$

and then by replacing $s_{\rm min}$ with Eq. (S7) and $s_{\rm max}$ with Eq. (S8), we find

$$k = \frac{(\alpha-1)(ab)^{\alpha} S S^{(\delta+\theta)\alpha}}{a b^{\alpha} S^{\delta+\theta\alpha} - a^{\alpha} b S^{\theta+\delta\alpha}}\,. \tag{S11}$$

By solving the integral in Eq. (S9), we find

$$Y = \frac{kc}{\beta-\alpha}\left(s_{\rm max}^{\beta-\alpha} - s_{\rm min}^{\beta-\alpha}\right)\,, \tag{S12}$$

and after replacing k by Eq. (S11), $s_{\rm min}$ by Eq. (S7), and $s_{\rm max}$ by Eq. (S8), we have

$$\begin{aligned} Y &= \frac{c(\alpha-1)S}{\alpha-\beta}\left(\frac{a^{\beta} b^{\alpha} S^{\delta\beta+\theta\alpha} - a^{\alpha} b^{\beta} S^{\theta\beta+\delta\alpha}}{a b^{\alpha} S^{\delta+\theta\alpha} - a^{\alpha} b S^{\theta+\delta\alpha}}\right) \\ &= \frac{c(\alpha-1)S}{\alpha-\beta}\left(\frac{a^{\beta} b^{\alpha} S^{\lambda_1} - a^{\alpha} b^{\beta} S^{\lambda_2}}{a b^{\alpha} S^{\lambda_3} - a^{\alpha} b S^{\lambda_4}}\right) \\ &= \frac{c(\alpha-1)S}{\alpha-\beta}\left(\frac{\Lambda_1 - \Lambda_2}{\Lambda_3 - \Lambda_4}\right)\,, \end{aligned} \tag{S13}$$

where

$$\begin{aligned} \lambda_1 &= \delta\beta + \theta\alpha \\ \lambda_2 &= \theta\beta + \delta\alpha \\ \lambda_3 &= \delta + \theta\alpha \\ \lambda_4 &= \theta + \delta\alpha\,. \end{aligned} \tag{S14}$$

and

$$
\begin{aligned}
\Lambda_1 &= a^{\beta} b^{\alpha} S^{\lambda_1} \\
\Lambda_2 &= a^{\alpha} b^{\beta} S^{\lambda_2} \\
\Lambda_3 &= a b^{\alpha} S^{\lambda_3} \\
\Lambda_4 &= a^{\alpha} b S^{\lambda_4} \,.
\end{aligned}
\tag{S15}
$$

Because S represents the total population of an urban system or a country, we take the limit of large S in Eq. (S13). To do so, we need to know the conditions under which Λ_1 dominates over Λ_2 in the numerator of Eq. (S13), that is, when

$$
\begin{aligned}
\lambda_1 &> \lambda_2 \\
\delta\beta + \theta\alpha &> \theta\beta + \delta\alpha \\
(\delta - \theta)(\beta - \alpha) &> 0 \,,
\end{aligned}
\tag{S16}
$$

and also the conditions under which Λ_3 dominates over Λ_4 in the denominator of Eq. (S13), that is, when

$$
\begin{aligned}
\lambda_3 &> \lambda_4 \\
\delta + \theta\alpha &> \theta + \delta\alpha \\
(\delta - \theta)(1 - \alpha) &> 0 \,.
\end{aligned}
\tag{S17}
$$

By investigating the inequality in Eq. (S16), we end up with the decision tree of Figure 12. Similarly, we find the decision tree of Figure 13 for the inequality in Eq. (S17).

Having the results of Figures 12 and 13, we can find the behavior of Y for large S in Eq. (S13) for each of the following conditions.

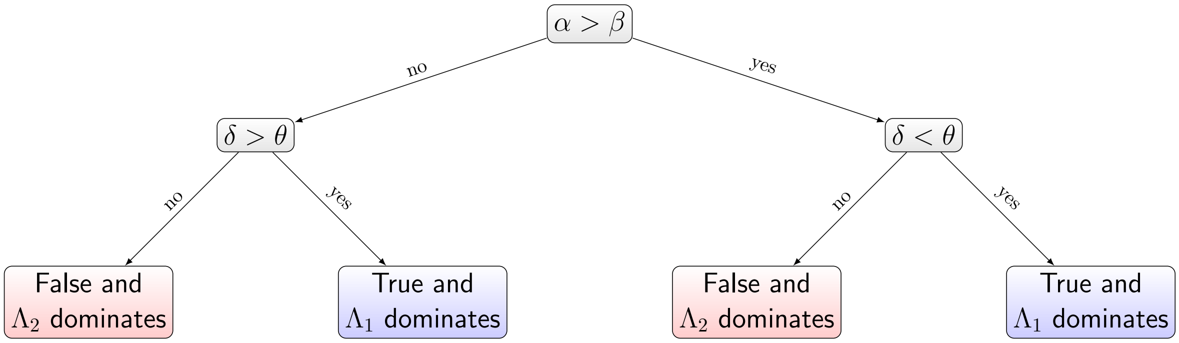

FIG. 12. Decision tree associated with the inequality $\lambda_1 > \lambda_2$ (Eq. S16) that defines whether Λ_1 dominates over Λ_2 (or vice-versa) in Eq. (S13).

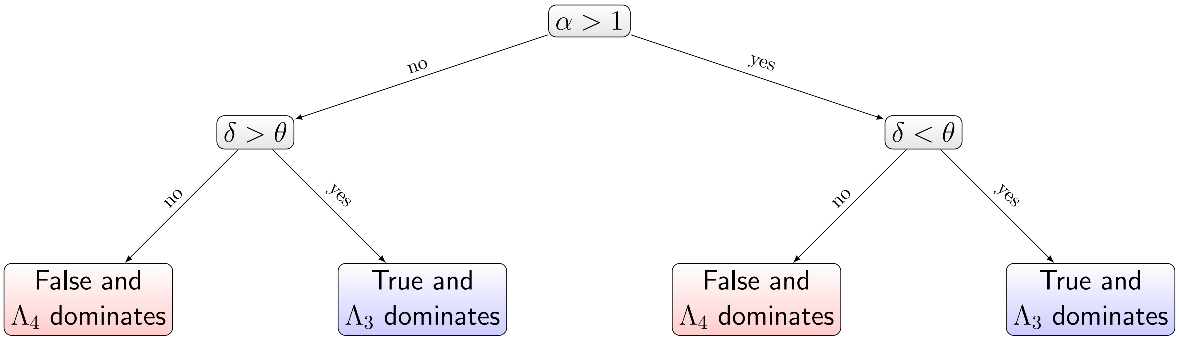

FIG. 13. Decision tree associated with the inequality $\lambda_3 > \lambda_4$ (Eq. S17) that defines whether Λ_3 dominates over Λ_4 (or vice-versa) in Eq. (S13).

Case A.1: $\alpha > \beta$, $\alpha > 1$, and $\delta < \theta$

Under these conditions, we find that Λ_1 and Λ_3 dominate in Eq. (S13), yielding

$$\begin{aligned} Y &\approx \frac{c(\alpha-1)a^{\beta-1}}{\alpha-\beta} S^{1+\lambda_1-\lambda_3} \\ &\approx \frac{c(\alpha-1)a^{\beta-1}}{\alpha-\beta} S^{1-\delta+\beta\delta} . \end{aligned} \tag{S18}$$

By comparing the result of Eq. (S18) with the country scaling in Eq. (S6), we find

$$\beta = 1 + \frac{\gamma - 1}{\delta} \qquad \text{for } \{\alpha > \beta \ \& \ \alpha > 1 \ \& \ \delta < \theta\} . \tag{S19}$$

It is worth noticing that the previous conditions depend on β, but by com-

bining $\alpha > \beta$ & $\alpha > 1$ and eliminating β, we can rewrite Eq. (S19) as

$$\beta = 1 + \frac{\gamma - 1}{\delta} \qquad \text{for } \left\{\alpha > \max\left(1, 1 + \frac{\gamma - 1}{\delta}\right) \ \& \ \delta < \theta\right\}. \tag{S20}$$

Equation (S20) thus predicts that β is a constant, that is, not dependent on α when $\{\alpha > \max\left(1, 1 + \frac{\gamma-1}{\delta}\right) \ \& \ \delta < \theta\}$.

Case A.2: $\alpha > \beta$, $\alpha < 1$, and $\delta < \theta$

For this case, we find that Λ_1 and Λ_4 dominate the behavior of Eq. (S13) for large S, yielding

$$\begin{aligned} Y &\approx \frac{c(1-\alpha)a^{\beta-\alpha}b^{\alpha-1}}{\alpha - \beta} S^{1+\lambda_1-\lambda_4} \\ &\approx \frac{c(1-\alpha)a^{\beta-\alpha}b^{\alpha-1}}{\alpha - \beta} S^{1-\theta+\beta\delta+\alpha(\theta-\delta)}. \end{aligned} \tag{S21}$$

By comparing the previous result with Eq. (S6), we find

$$\beta = \frac{\gamma + \theta - 1}{\delta} + \left(1 - \frac{\theta}{\delta}\right)\alpha \qquad \text{for } \{\alpha > \beta \ \& \ \alpha < 1 \ \& \ \delta < \theta\}, \tag{S22}$$

and after eliminating β from the condition, we have

$$\beta = \frac{\gamma + \theta - 1}{\delta} + \left(1 - \frac{\theta}{\delta}\right)\alpha \qquad \text{for } \left\{\gamma < 1 \ \& \ 1 + \frac{\gamma - 1}{\theta} < \alpha < 1 \ \& \ \delta < \theta\right\}. \tag{S23}$$

Equation (S23) thus predicts a linear decreasing correspondence between β and α when $\{\gamma < 1 \ \& \ 1 + \frac{\gamma-1}{\theta} < \alpha < 1 \ \& \ \delta < \theta\}$.

Case A.3: $\alpha < \beta$, $\alpha > 1$, and $\delta < \theta$

Under these assumptions, Λ_2 and Λ_3 dominate the behavior of Eq. (S13) for large S, leading to

$$\begin{aligned} Y &\approx \frac{c(\alpha-1)a^{\alpha-1}b^{\beta-\alpha}}{\beta - \alpha} S^{1+\lambda_2-\lambda_3} \\ &\approx \frac{c(\alpha-1)a^{\alpha-1}b^{\beta-\alpha}}{\beta - \alpha} S^{1-\delta+\beta\theta+\alpha(\delta-\theta)}. \end{aligned} \tag{S24}$$

By comparing the previous result with Eq. (S6), we find

$$\beta = \frac{\gamma + \delta - 1}{\theta} + \left(1 - \frac{\delta}{\theta}\right)\alpha \qquad \text{for } \{\alpha < \beta \ \& \ \alpha > 1 \ \& \ \delta < \theta\}, \tag{S25}$$

that after eliminating β from the condition leads to

$$\beta = \frac{\gamma + \delta - 1}{\theta} + \left(1 - \frac{\delta}{\theta}\right)\alpha \qquad \text{for } \left\{\gamma > 1 \ \& \ 1 < \alpha < 1 + \frac{\gamma - 1}{\delta} \ \& \ \delta < \theta\right\} . \tag{S26}$$

Similarly to Eq. (S23), the result of Eq. (S26) predicts a linear increasing correspondence between β and α when $\{\gamma > 1 \ \& \ 1 < \alpha < 1 + \frac{\gamma-1}{\delta} \ \& \ \delta < \theta\}$. It is worth noticing that the conditions underlying Eqs. (S23) and (S26) are mutually exclusive, that is, only one of the linear correspondences exists depending on whether $\gamma > 1$ (super-linear country scaling, Eq. S26) or $\gamma < 1$ (sub-linear country scaling, Eq. S23).

Case A.4: $\alpha < \beta$, $\alpha < 1$, and $\delta < \theta$

For this case, we find that Λ_2 and Λ_4 dominate the behavior of Eq. (S13) for large S, resulting in

$$\begin{aligned} Y &\approx \frac{c(1-\alpha)b^{\beta-1}}{\beta-\alpha} S^{1+\lambda_2-\lambda_4} \\ &\approx \frac{c(1-\alpha)b^{\beta-1}}{\beta-\alpha} S^{1-\theta+\beta\theta} . \end{aligned} \tag{S27}$$

By comparing the previous result with Eq. (S6), we find

$$\beta = 1 + \frac{\gamma - 1}{\theta} \qquad \text{for } \{\alpha < \beta \ \& \ \alpha < 1 \ \& \ \delta < \theta\} , \tag{S28}$$

and after eliminating β from the condition, we have

$$\beta = 1 + \frac{\gamma - 1}{\theta} \qquad \text{for } \left\{\alpha < \min\left(1, 1 + \frac{\gamma - 1}{\theta}\right) \ \& \ \delta < \theta\right\} . \tag{S29}$$

Equation (S29) indicates that β is a constant when $\{\alpha < \min\left(1, 1 + \frac{\gamma-1}{\theta}\right) \ \& \ \delta < \theta\}$.

If we assume that γ, δ and θ (with $\delta < \theta$) are fixed, the combined behavior of Eqs. (S20), (S23), (S26), and (S29) produces a functional dependence of β on α composed by three continuous line segments: an initial horizontal plateau (Eq. S29) followed by an increasing (Eq. S26, when $\gamma > 1$) or decreasing (Eq. S23, when $\gamma < 1$) linear function followed by another horizontal plateau (Eq. S20).

Case A.5: $\alpha > \beta$, $\alpha > 1$, and $\delta > \theta$

Under these conditions, Λ_2 and Λ_4 dominate the behavior of Eq. (S13) for large S, yielding

$$\begin{aligned} Y &\approx \frac{c(\alpha-1)b^{\beta-1}}{\alpha-\beta} S^{1+\lambda_2-\lambda_4} \\ &\approx \frac{c(\alpha-1)b^{\beta-1}}{\alpha-\beta} S^{1-\theta+\beta\theta} \,. \end{aligned} \tag{S30}$$

By comparing the previous result with Eq. (S6), we find

$$\beta = 1 + \frac{\gamma-1}{\theta} \qquad \text{for } \{\alpha > \beta \text{ \& } \alpha > 1 \text{ \& } \delta > \theta\}\,, \tag{S31}$$

and after eliminating β from the condition, we have

$$\beta = 1 + \frac{\gamma-1}{\theta} \qquad \text{for } \left\{\alpha > \max\left(1, 1+\frac{\gamma-1}{\theta}\right) \text{ \& } \delta > \theta\right\}\,. \tag{S32}$$

Equation (S32) thus predicts that β is a constant when $\{\alpha > \max\left(1, 1+\frac{\gamma-1}{\theta}\right) \text{ \& } \delta > \theta\}$.

Case A.6: $\alpha > \beta$, $\alpha < 1$, and $\delta > \theta$

For this case, Λ_2 and Λ_3 dominate the behavior of Eq. (S13) for large S and produce

$$\begin{aligned} Y &\approx \frac{c(1-\alpha)a^{\alpha-1}b^{\beta-\alpha}}{\alpha-\beta} S^{1+\lambda_2-\lambda_3} \\ &\approx \frac{c(1-\alpha)a^{\alpha-1}b^{\beta-\alpha}}{\alpha-\beta} S^{1-\delta+\beta\theta+\alpha(\delta-\theta)} \,. \end{aligned} \tag{S33}$$

By comparing the previous result with Eq. (S6), we find

$$\beta = \frac{\gamma+\delta-1}{\theta} + \left(1-\frac{\delta}{\theta}\right)\alpha \qquad \text{for } \{\alpha > \beta \text{ \& } \alpha < 1 \text{ \& } \delta > \theta\}\,, \tag{S34}$$

and after eliminating β from the condition, we have

$$\beta = \frac{\gamma+\delta-1}{\theta} + \left(1-\frac{\delta}{\theta}\right)\alpha \qquad \text{for } \left\{\gamma < 1 \text{ \& } 1+\frac{\gamma-1}{\delta} < \alpha < 1 \text{ \& } \delta > \theta\right\}\,. \tag{S35}$$

Equation (S35) predicts a linear decreasing correspondence between β and α when $\{\gamma < 1 \text{ \& } 1+\frac{\gamma-1}{\delta} < \alpha < 1 \text{ \& } \delta > \theta\}$.

Case A.7: $\alpha < \beta$, $\alpha > 1$, and $\delta > \theta$

For this case, Λ_1 and Λ_4 dominate the behavior of Eq. (S13), yielding

$$\begin{aligned} Y &\approx \frac{c(\alpha-1)a^{\beta-\alpha}b^{\alpha-1}}{\beta-\alpha} S^{1+\lambda_1-\lambda_4} \\ &\approx \frac{c(\alpha-1)a^{\beta-\alpha}b^{\alpha-1}}{\beta-\alpha} S^{1-\theta+\beta\delta+\alpha(\theta-\delta)} \,. \end{aligned} \tag{S36}$$

By comparing the previous result with Eq. (S6), we find

$$\beta = \frac{\gamma+\theta-1}{\delta} + \left(1-\frac{\theta}{\delta}\right)\alpha \qquad \text{for } \{\alpha < \beta \;\&\; \alpha > 1 \;\&\; \delta > \theta\}\,, \tag{S37}$$

and after eliminating β from the condition, we have

$$\beta = \frac{\gamma+\theta-1}{\delta} + \left(1-\frac{\theta}{\delta}\right)\alpha \qquad \text{for } \left\{\gamma > 1 \;\&\; 1 < \alpha < 1 + \frac{\gamma-1}{\theta} \;\&\; \delta > \theta\right\}\,. \tag{S38}$$

Equation (S38) predicts a linear increasing correspondence between β and α when $\{\gamma > 1 \;\&\; 1 < \alpha < 1 + \frac{\gamma-1}{\theta} \;\&\; \delta > \theta\}$. Similarly to cases A.2 and A.3, the conditions underlying Eqs. (S35) and (S38) are mutually exclusive, that is, only one of the linear correspondences exists depending on whether $\gamma > 1$ (super-linear country scaling, Eq. S38) or $\gamma < 1$ (sub-linear country scaling, Eq. S35).

Case A.8: $\alpha < \beta$, $\alpha < 1$, and $\delta > \theta$

Under these conditions, Λ_1 and Λ_3 dominate the behavior of Eq. (S13), yielding

$$\begin{aligned} Y &\approx \frac{c(1-\alpha)a^{\beta-1}}{\beta-\alpha} S^{1+\lambda_1-\lambda_3} \\ &\approx \frac{c(1-\alpha)a^{\beta-1}}{\beta-\alpha} S^{1-\delta+\beta\delta} \,. \end{aligned} \tag{S39}$$

By comparing the previous result with Eq. (S6), we find

$$\beta = 1 + \frac{\gamma-1}{\delta} \qquad \text{for } \{\alpha < \beta \;\&\; \alpha < 1 \;\&\; \delta > \theta\}\,, \tag{S40}$$

and after eliminating β from the condition, we have

$$\beta = 1 + \frac{\gamma-1}{\delta} \qquad \text{for } \left\{\alpha < \min\left(1, 1 + \frac{\gamma-1}{\delta}\right) \;\&\; \delta > \theta\right\}\,. \tag{S41}$$

Equation (S41) predicts that β is a constant when $\{\alpha < \min\left(1, 1 + \frac{\gamma-1}{\delta}\right)$ & $\delta > \theta\}$.

Similarly to cases A.1-A.4, the combined behavior of Eqs. (S32), (S35), (S38) and (S41) produces a functional dependence of β on α composed by three continuous line segments: an initial horizontal plateau (Eq. S41) followed by an increasing (Eq. S38, when $\gamma > 1$) or decreasing (Eq. S35, when $\gamma < 1$) linear function followed by another horizontal plateau (Eq. S32).

Case B: $\alpha = 1$ and $\beta \neq 1$

We now consider the particular case $\alpha = 1$ and $\beta \neq 1$ in order to ensure the continuous behavior of β as function of α at $\alpha = 1$. Under these assumptions, the normalization constant in Eq. (S4) is

$$k = \frac{S}{\ln\left(\frac{s_{\max}}{s_{\min}}\right)}, \tag{S42}$$

and by replacing $s_{\min}$ and $s_{\max}$ with Eqs. (S7) and (S8), we find

$$k = \frac{S}{\ln\left(\frac{b}{a}S^{\theta-\delta}\right)}. \tag{S43}$$

The solution of the integral in Eq. (S9) for $\alpha = 1$ is

$$Y = \frac{kc}{\beta - 1}\left(s_{\max}^{\beta-1} - s_{\min}^{\beta-1}\right), \tag{S44}$$

and after replacing k by Eq. (S43), $s_{\min}$ by Eq. (S7), and $s_{\max}$ by Eq. (S8), we find

$$\begin{aligned} Y &= \frac{cS}{\beta - 1}\left(\frac{b^{\beta-1}S^{-\theta+\beta\theta} - a^{\beta-1}S^{-\delta+\beta\delta}}{\ln\left(\frac{b}{a}S^{\theta-\delta}\right)}\right) \\ &= \frac{cS}{\beta - 1}\left(\frac{\Omega_1 - \Omega_2}{\ln\left(\frac{b}{a}S^{\theta-\delta}\right)}\right), \end{aligned} \tag{S45}$$

where

$$\begin{aligned} \Omega_1 &= b^{\beta-1}S^{-\theta+\beta\theta} \\ \Omega_2 &= a^{\beta-1}S^{-\delta+\beta\delta} \end{aligned}. \tag{S46}$$

It is worth noticing that Eq. (S45) does not produce a “pure” power-law behavior for large S due to the logarithmic function in its denominator. However, the logarithmic function changes much slower than the power-law functions in the numerator, allowing us to approximate the behavior of Eq. (S45) by a power-law function for large S.

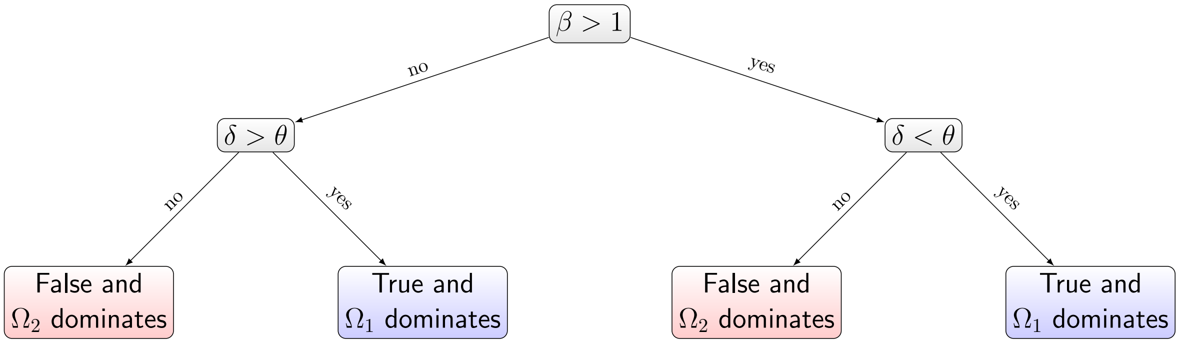

FIG. 14. Decision tree associated with the inequality in Eq. (S47) that defines whether Ω_1 dominates over Ω_2 (or vice-versa) in Eq. (S45).

To estimate the behavior of Eq. (S45) for large values of S, we need to determine the conditions under which Ω_1 dominates over Ω_2, that is, when

$$\begin{aligned} -\theta + \beta\theta &> -\delta + \beta\delta \\ (\delta - \theta)(1 - \beta) &> 0\,. \end{aligned} \tag{S47}$$

The inequality in Eq. (S47) leads us to the decision tree of Figure 14, and from these results, we can find the behavior of Y for large S in Eq. (S45) for each of the following conditions.

Case B.1: $\beta > 1$ and $\delta < \theta$

Under these conditions, Ω_1 dominates the behavior of Eq. (S45) for large S, leading us to

$$Y = \frac{cb^{\beta-1}}{\beta - 1}\left(\frac{S^{1-\theta+\beta\theta}}{\ln\left(\frac{b}{a}S^{\theta-\delta}\right)}\right)\,. \tag{S48}$$

By comparing the previous result with Eq. (S6) and considering that $\gamma = 1 - \theta + \beta\theta$, we find

$$\beta = 1 + \frac{\gamma - 1}{\theta} \qquad \text{for } \{\alpha = 1 \;\&\; \beta > 1 \;\&\; \delta < \theta\}\,. \tag{S49}$$

This result is the same as obtained for case A.4 and ensures the continuity of the behavior of β as a function of α at the point $\alpha = 1$.

Case B.2: $\beta < 1$ and $\delta < \theta$

For this case, Ω_2 dominates the behavior of Eq. (S45) for large S, leading to

$$Y = \frac{ca^{\beta-1}}{1-\beta}\left(\frac{S^{1-\delta+\beta\delta}}{\ln\left(\frac{b}{a}S^{\theta-\delta}\right)}\right). \tag{S50}$$

By comparing the previous result with Eq. (S6) and considering that $\gamma = 1-\delta+\beta\delta$, we find

$$\beta = 1 + \frac{\gamma-1}{\delta} \qquad \text{for } \{\alpha = 1 \;\&\; \beta < 1 \;\&\; \delta < \theta\}. \tag{S51}$$

This result is the same as obtained for case A.1 and ensures the continuity of the behavior of β as a function of α at the point $\alpha = 1$.

Case B.3: $\beta > 1$ and $\delta > \theta$

For this case, Ω_2 dominates the behavior of Eq. (S45) for large S, leading us to

$$Y = \frac{-ca^{\beta-1}}{\beta-1}\left(\frac{S^{1-\delta+\beta\delta}}{\ln\left(\frac{b}{a}S^{\theta-\delta}\right)}\right). \tag{S52}$$

It is worth noticing that Eq. (S52) yields positive values for Y since the logarithmic function is negative when $\delta > \theta$ and for large S. By comparing the previous result with Eq. (S6) and considering that $\gamma = 1-\delta+\beta\delta$, we find

$$\beta = 1 + \frac{\gamma-1}{\delta} \qquad \text{for } \{\alpha = 1 \;\&\; \beta > 1 \;\&\; \delta > \theta\}. \tag{S53}$$

This result is the same as obtained for case A.8 and ensures the continuity of the behavior of β as a function of α at the point $\alpha = 1$.

Case B.4: $\beta < 1$ and $\delta > \theta$

For this case, Ω_1 dominates the behavior of Eq. (S45) for large S, leading us to

$$Y = \frac{-cb^{\beta-1}}{1-\beta}\left(\frac{S^{1-\theta+\beta\theta}}{\ln\left(\frac{b}{a}S^{\theta-\delta}\right)}\right). \tag{S54}$$

It is worth noticing that Eq. (S54) yields positive values for Y since the logarithmic function is negative when $\delta > \theta$ and for large S. By comparing the previous result with Eq. (S6) and considering that $\gamma = 1 - \theta + \beta\theta$, we find

$$\beta = 1 + \frac{\gamma - 1}{\theta} \qquad \text{for } \{\alpha = 1 \ \& \ \beta < 1 \ \& \ \delta > \theta\} \,. \tag{S55}$$

This result is the same as obtained for case A.5 and ensures the continuity of the behavior of β as a function of α at the point $\alpha = 1$.

2. FUNCTIONAL DEPENDENCE BETWEEN URBAN SCALING AND ZIPF EXPONENTS

At this point, we can combine the results of cases A1-A8 and write the functional dependence of β on α for each of the following conditions.

For $\gamma > 1$ and $\delta < \theta$: By combining the cases A.1, A.3 and A.4, we find

$$\beta = \begin{cases} 1 + \frac{\gamma-1}{\theta} & 0 < \alpha \leq 1 \\ \frac{\gamma+\delta-1}{\theta} + \left(1 - \frac{\delta}{\theta}\right)\alpha & 1 < \alpha < 1 + \frac{\gamma-1}{\delta} \\ 1 + \frac{\gamma-1}{\delta} & \alpha \geq 1 + \frac{\gamma-1}{\delta} \end{cases} . \tag{S56}$$

Equation (S56) represents three continuous line segments: an initial horizontal plateau ($\alpha \leq 1$) followed by an increasing linear function ($1 < \alpha < 1 + \frac{\gamma-1}{\delta}$) followed by another horizontal plateau ($\alpha \geq 1 + \frac{\gamma-1}{\delta}$) higher than the first one. Figure 15 illustrates the typical behavior of Eq. (S56).

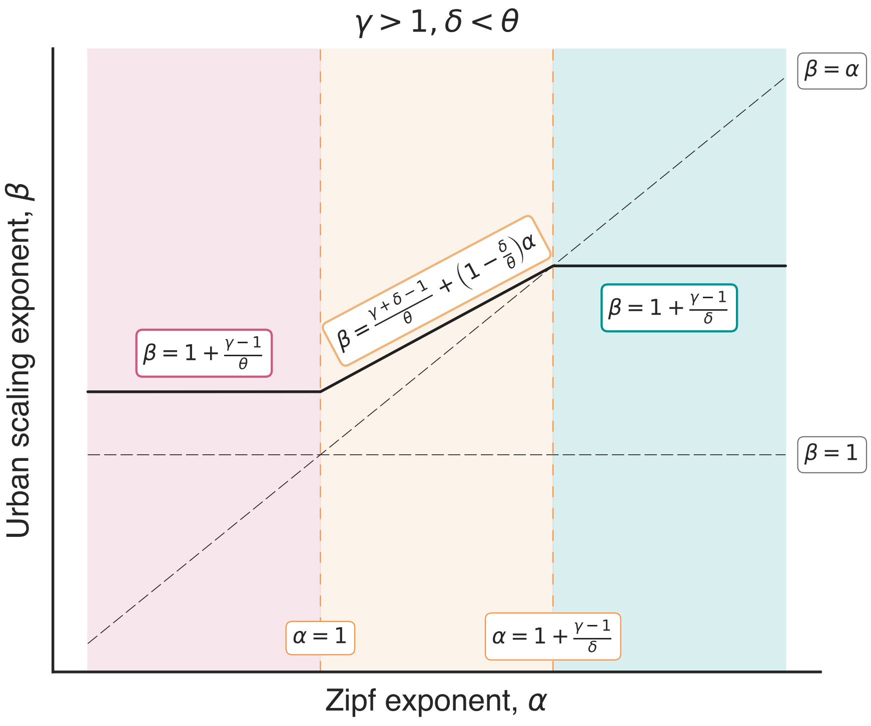

FIG. 15. Illustration of the relation between β and α when $\gamma > 1$ and $\delta < \theta$ predicted by Eq. (S56).

For $\gamma > 1$ and $\delta > \theta$: By combining the cases A.5, A.7 and A.8, we find

$$\beta = \begin{cases} 1 + \frac{\gamma-1}{\delta} & 0 < \alpha \le 1 \\ \frac{\gamma+\theta-1}{\delta} + \left(1 - \frac{\theta}{\delta}\right)\alpha & 1 < \alpha < 1 + \frac{\gamma-1}{\theta} \\ 1 + \frac{\gamma-1}{\theta} & \alpha \ge 1 + \frac{\gamma-1}{\theta} \end{cases} . \tag{S57}$$

Equation (S57) represents three continuous line segments: an initial horizontal plateau ($\alpha \le 1$) followed by an increasing linear function ($1 < \alpha < 1 + \frac{\gamma-1}{\theta}$) followed by another horizontal plateau ($\alpha \ge 1 + \frac{\gamma-1}{\theta}$) higher than the first one. It is worth noticing that we can pass back and forth from Eq. (S56) to Eq. (S57) by replacing δ by θ.

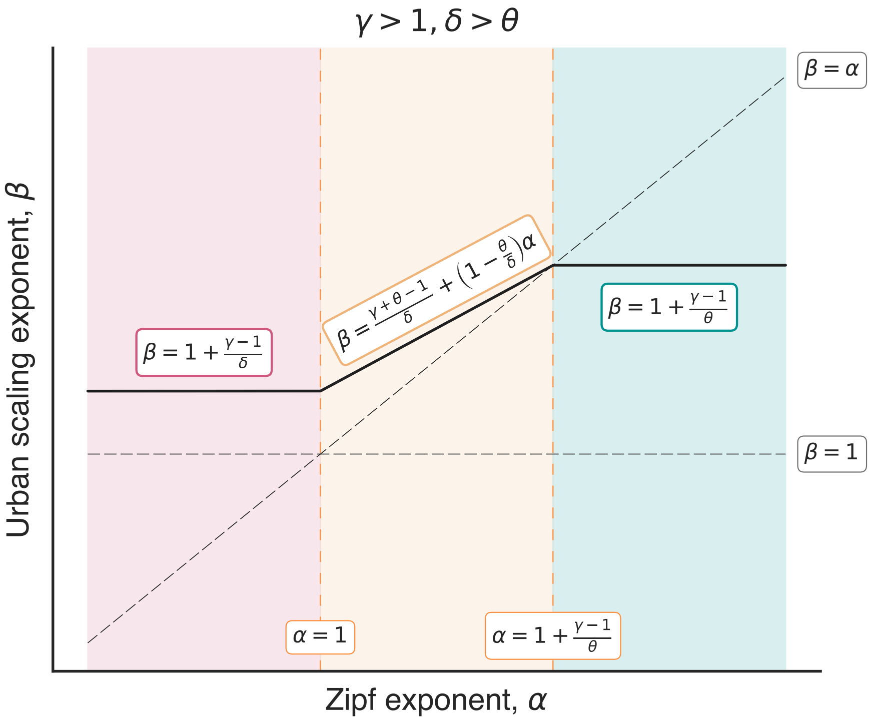

FIG. 16. Illustration of the relation between β and α when $\gamma > 1$ and $\delta > \theta$ predicted by Eq. (S57).

For $\gamma < 1$ and $\delta < \theta$: By combining the cases A.1, A.2 and A.4, we find

$$\beta = \begin{cases} 1 + \frac{\gamma-1}{\theta} & 0 < \alpha \leq 1 + \frac{\gamma-1}{\theta} \\ \frac{\gamma+\theta-1}{\delta} + \left(1 - \frac{\theta}{\delta}\right)\alpha & 1 + \frac{\gamma-1}{\theta} < \alpha < 1 \\ 1 + \frac{\gamma-1}{\delta} & \alpha \geq 1 \end{cases} . \tag{S58}$$

Equation (S58) represents three continuous line segments: an initial horizontal plateau ($\alpha \leq 1 + \frac{\gamma-1}{\theta}$) followed by a decreasing linear function ($1 + \frac{\gamma-1}{\theta} < \alpha < 1$) followed by another horizontal plateau ($\alpha \geq 1$) lower than the first one.

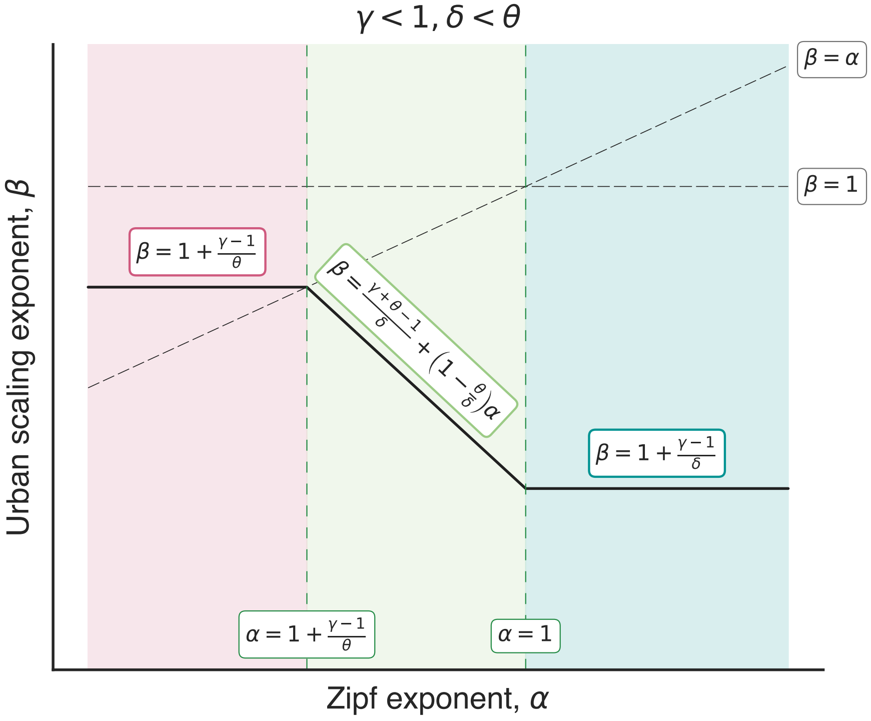

FIG. 17. Illustration of the relation between β and α when $\gamma < 1$ and $\delta < \theta$ predicted by Eq. (S58).

For $\gamma < 1$ and $\delta > \theta$: By combining the cases A.5, A.6 and A.8, we find

$$\beta = \begin{cases} 1 + \frac{\gamma-1}{\delta} & 0 < \alpha \leq 1 + \frac{\gamma-1}{\delta} \\ \frac{\gamma+\delta-1}{\theta} + \left(1 - \frac{\delta}{\theta}\right)\alpha & 1 + \frac{\gamma-1}{\delta} < \alpha < 1 \\ 1 + \frac{\gamma-1}{\theta} & \alpha \geq 1 \end{cases} . \tag{S59}$$

Equation (S59) represents three continuous line segments: an initial horizontal plateau ($\alpha \leq 1 + \frac{\gamma-1}{\delta}$) followed by a decreasing linear function ($1 + \frac{\gamma-1}{\delta} < \alpha < 1$) followed by another horizontal plateau ($\alpha \geq 1$) lower than the first one. It is worth noticing that we can pass back and forth from Eq. (S58) to Eq. (S59) by replacing δ by θ.

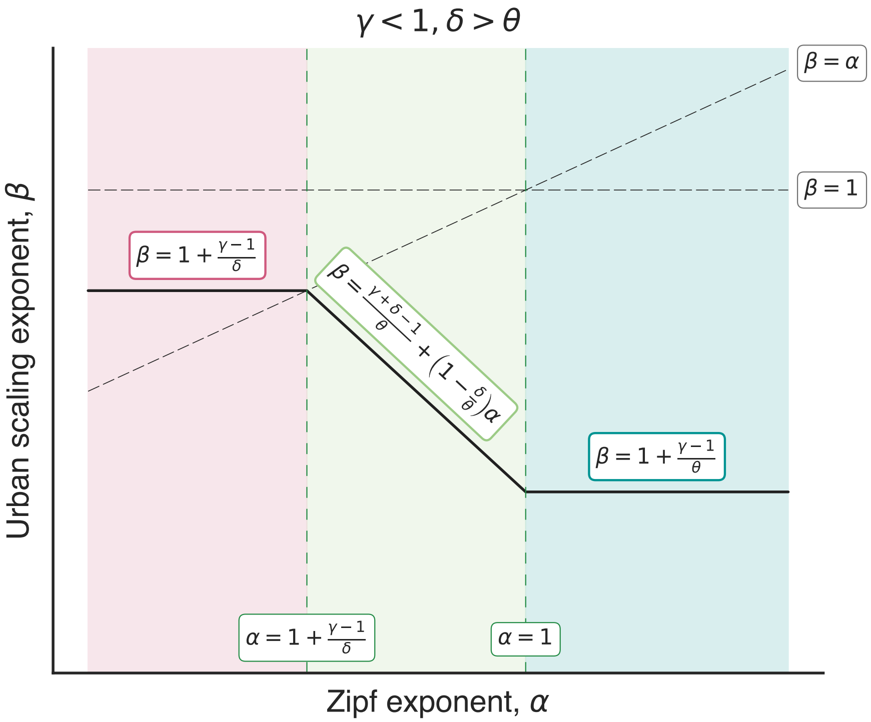

FIG. 18. Illustration of the relation between β and α when $\gamma < 1$ and $\delta > \theta$ predicted by Eq. (S59).

For $\delta = \theta = \varphi$: By combining the results of Eqs. (S56), (S57), (S58), and (S59), we find

$$\beta = 1 + \frac{\gamma - 1}{\varphi} \qquad \text{for } \alpha > 0\,, \tag{S60}$$

regardless of $\gamma > 1$ or $\gamma < 1$. Thus, we have that β is constant for all values of α when $\delta = \theta$. It is also interesting to note that $\beta = 1$ when $\gamma = 1$, that is, an isometric country scaling implies in isometric urban scaling relationships.

For $\gamma = 1$: By combining the results of Eqs. (S56), (S57), (S58), and (S59), we find

$$\beta = 1 \tag{S61}$$

regardless of $\gamma > 1$ or $\gamma < 1$. Thus, constant returns to scale at the country level imply in constant returns to scale at the city level for all values of α.

3. NUMERICAL SIMULATIONS OF THE CONNECTION BETWEEN URBAN SCALING AND ZIPF EXPONENTS

In order to understand how variations in the country scaling and urban scaling relationships affect the exact expressions of Sec. 2, we have designed a numerical experiment to simulate the empirical relation between β and α. We begin by generating data at the city level, that is, given the total population S, total indicator Y, and Zipf exponent α for a country, we generate a list of m population values

$$\mathcal{S} = \{s_1, s_2, \dots, s_i, \dots, s_m\}\,, \tag{S62}$$

where each s_i is a random number drawn from a truncated power-law distribution within the interval $(s_{\max}, s_{\min})$ with exponent $\alpha + 1$ (Eq. S2), and a list of corresponding urban indicators

$$\mathcal{Y} = \{y_1, y_2, \dots, y_i, \dots, y_m\}\,, \tag{S63}$$

where each indicator y_i follows the urban scaling law (Eq. S5) with randon variations

$$\begin{aligned} y_i &= c\, s_i^{\beta(\alpha,\gamma,\delta,\theta)}\; 10^{\mathcal{N}(0,\sigma_y)} \\ \log y_i &= \log c + \beta(\alpha,\gamma,\delta,\theta) \log s_i + \mathcal{N}(0,\sigma_y), \end{aligned} \tag{S64}$$

where c is a constant, $\beta(\alpha,\gamma,\delta,\theta)$ represents the functional relationship between the urban scaling exponent and the Zipf exponent (defined by Eqs. S56-S59) for given values of γ, δ and θ), and $\mathcal{N}(0,\sigma_y)$ is Gaussian random variable with zero mean and variance σ_y^2. The term $\mathcal{N}(0,\sigma_y)$ introduces random variations into the urban scaling relationship, but has a small impact on the country scaling relationships. Thus, in order to account for random variations in the country scaling relationships, we have included analogous terms into Eqs. (S7) and (S8), that is,

$$\begin{aligned} s_{\min} &= a\, S^{\delta}\; 10^{\mathcal{N}(0,\sigma_\delta)} \\ \log s_{\min} &= \log a + \delta \log S + \mathcal{N}(0,\sigma_\delta) \end{aligned} \tag{S65}$$

and

$$\begin{aligned} s_{\max} &= a\, S^{\theta}\; 10^{\mathcal{N}(0,\sigma_\theta)} \\ \log s_{\max} &= \log a + \theta \log S + \mathcal{N}(0,\sigma_\theta) \end{aligned}, \tag{S66}$$

where $\mathcal{N}(0,\sigma_\delta)$ and $\mathcal{N}(0,\sigma_\theta)$ are Gaussian random variables with zero mean, and variances σ_γ^2 and σ_δ^2, respectively. Finally, we have further considered random variations in the country scaling relationship between Y and S by adding a random term to the total country population, that is,

$$S \to S\, 10^{\mathcal{N}(0,\sigma_\gamma)}\,, \tag{S67}$$

where $\mathcal{N}(0,\sigma_\gamma)$ is a Gaussian random variable with zero mean and variance σ_γ^2.

In our simulations the values s_i and y_i should satisfy the constraints $\sum_{i=1}^m s_i \approx S$ and $\sum_{i=1}^m y_i \approx Y$. We satisfy the first constraint by iteratively generating values of s_i and appending to $\mathcal{S}$ until $\sum_{i=1}^m s_i \le S$. To fulfill the second constraint, we define the values of c by comparing Eq. (S6) with results of Case A (Eqs. S18, S21, S24, S27, S30, S33, S36, and S39), that is,

$$c(\alpha,\beta,\delta,\theta,a,b,Y_0) = Y_0 \times \begin{cases} \frac{\alpha-\beta}{(\alpha-1)a^{\beta-1}} & \text{for } \{\alpha>\beta \;\&\; \alpha>1 \;\&\; \delta<\theta\} \\ \frac{\alpha-\beta}{(1-\alpha)a^{\beta-\alpha}b^{\alpha-1}} & \text{for } \{\alpha>\beta \;\&\; \alpha<1 \;\&\; \delta<\theta\} \\ \frac{\beta-\alpha}{(\alpha-1)a^{\alpha-1}b^{\beta-\alpha}} & \text{for } \{\alpha<\beta \;\&\; \alpha>1 \;\&\; \delta<\theta\} \\ \frac{\beta-\alpha}{(1-\alpha)b^{\beta-1}} & \text{for } \{\alpha<\beta \;\&\; \alpha<1 \;\&\; \delta<\theta\} \\ \frac{\alpha-\beta}{(\alpha-1)b^{\beta-1}} & \text{for } \{\alpha>\beta \;\&\; \alpha>1 \;\&\; \delta>\theta\} \\ \frac{\alpha-\beta}{(1-\alpha)a^{\alpha-1}b^{\beta-\alpha}} & \text{for } \{\alpha>\beta \;\&\; \alpha<1 \;\&\; \delta>\theta\} \\ \frac{\beta-\alpha}{(\alpha-1)a^{\beta-\alpha}b^{\alpha-1}} & \text{for } \{\alpha<\beta \;\&\; \alpha>1 \;\&\; \delta>\theta\} \\ \frac{\beta-\alpha}{(1-\alpha)a^{\beta-1}} & \text{for } \{\alpha<\beta \;\&\; \alpha<1 \;\&\; \delta>\theta\} \end{cases}, \tag{S68}$$

where the value of Y_0 is obtained from the fit of Eq. (S6) to the empirical relationship between Y and S (Fig. 2 of main text).

Algorithm 1 shows the numerical procedures used in our simulations. This algorithm defines the function `generate_country_data` that takes the country population (S), the power-law exponents (α, γ, δ, and θ), some other constants (Y_0, a, and b), and the parameters related to the intensity of the random variations ($\sigma_y, \sigma_\gamma, \sigma_\delta$, and σ_θ) as arguments and returns the simulated lists of populations $\mathcal{S}$ and urban indicators $\mathcal{Y}$. Having these lists, we estimate the simulated exponent $\tilde{\beta}$ in the same way we have proceeded with the empirical data, that is, via robust linear regression of the relationship

Algorithm 1 Algorithm for generating a list of city populations ($\mathcal{S}$) and a list of urban indicators ($\mathcal{Y}$) for a country with total population S.

```
(𝒮, 𝒴) = generate_country_data(S, α, γ, δ, θ, Y_0, a, b, σ_y, σ_γ, σ_δ, σ_θ)
    S = S 10^{𝒩(0,σ_γ)}            ▷ 𝒩(μ, σ) is Gaussian random variate with mean μ and variance σ^2
    s_min = a S^δ 10^{𝒩(0,σ_δ)}
    s_max = b S^θ 10^{𝒩(0,σ_θ)}
    S̃ = 0
    𝒮 = []
    𝒴 = []
    β = β(α, γ, δ, θ)                ▷ Defined from Eqs. (S56), (S57), (S58), and (S59)
    c = c(α, β, δ, θ, a, b, Y_0)      ▷ Defined from Eq. (S68)
    while S̃ < S do
        s_i = 𝒫(α + 1, s_min, s_max)  ▷ 𝒫(ν, x_min, x_max) is power-law random variate
                                      ▷ with exponent ν in the interval (x_min, x_max)
        y_i = c s_i^β 10^{𝒩(0,σ_y)}
        S̃ = S̃ + s_i
        𝒮 ⟵ s_i                      ▷ Appends s_i to the list of city populations 𝒮
        𝒴 ⟵ y_i                      ▷ Appends y_i to the list of city indicators 𝒴
    end while
```

$\log y_i$ versus $\log s_i$. We also obtain the simulated values for total population $\tilde{S}$ and total indicator $\tilde{Y}$ by summing up the values of s_i and y_i. Similarly, we estimate the simulated values of $\tilde{s}_{\text{min}}$ and $\tilde{s}_{\text{max}}$ by taking the maximum and minimum values within the list $\mathcal{S}$.

For our simulations, we use the total population (of all cities within the power-law regime) of each country in our data set as values of S. For each of these values of S, we also use the corresponding estimated Zipf exponents α. In addition, we set up the values γ, δ, and θ equal to the best fitting values of the empirical relationship between β and α. The constants Y_0, a, and b do not affect the relationship between $\tilde{\alpha}$ and $\tilde{\beta}$, and have been chosen to match the fitted values obtained from the country scaling relationships (with fixed power-laws exponents). We also set the parameters σ_γ, σ_δ and σ_θ equal to the standard deviations of the bootstrap estimates of the empirical exponents γ, δ, and θ, respectively. Finally, we have varied the parameter σ_y and obtained the simulated relationship between $\tilde{\beta}$ and $\tilde{\alpha}$. The same approach is used when considering that s_i follows a power-law distribution with exponential

cutoff, that is,

$$p(s) \sim s^{-(\alpha+1)} \exp(-s/s_c) \tag{S69}$$

where $s_c > 0$ is an additional parameter. The only change necessary to implement this case is to replace the truncated power-law random number $\mathcal{P}(\alpha+1, s_{\min}, s_{\max})$ in Algorithm 1 by an a random number generator associated with Eq. (S69), that is, a $\mathcal{P}(\alpha+1, s_{\min}, s_c)$.

4. ZIPF AND URBAN SCALING PLOTS FOR EACH COUNTRY

In this section, we show Zipf's law and urban scaling law adjusted for every country in our data set. In what follows, each row of panels shows the results for a given country (name is indicated within the plots). The first column shows the complementary cumulative distribution $F(s)$ of city population s (gray continuous line) and the adjusted power-law distribution (dashed line). The second column shows the rank plot, where each city is named within the plots. The dashed lines show the adjusted Zipf's law. The power-law exponent α ($\pm$ standard error) and the lower cutoff population size s_{smin} ($\pm$ standard error) are estimated from the approach of Clauset-Shalizi-Newman [SIAM Review 51, 661 (2009)]. The third column shows urban scaling between urban GDP and city population, where each city is also named within the plots. The dashed line represents the adjusted scaling law. The urban scaling exponent β ($\pm$ standard error) is estimated via robust linear regression on the log-transformed quantities.

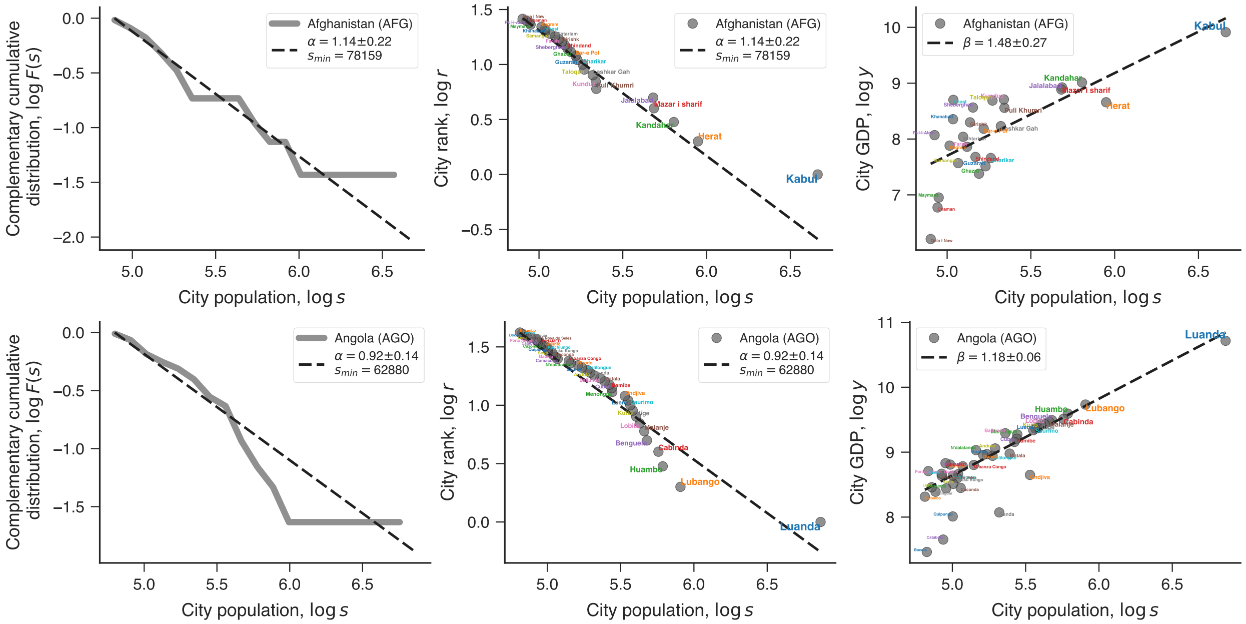

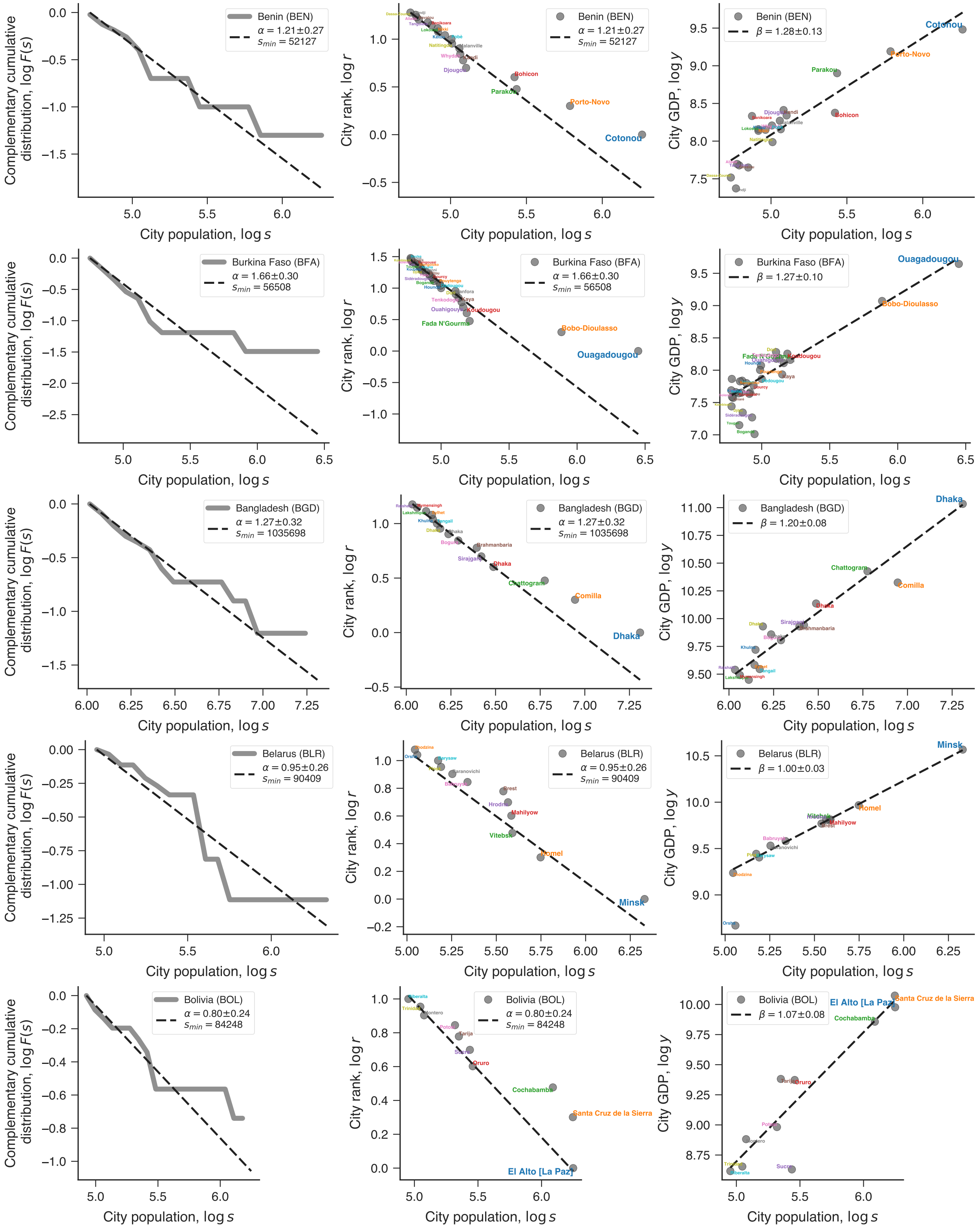

Complementary cumulative distribution, log F(s)
City rank, log r
City GDP, log y
City population, log s
Benin (BEN)
α = 1.21±0.27
s_min = 52127
β = 1.28±0.13
Cotonou
Porto-Novo
Parakou
Bohicon
Djougou
Burkina Faso (BFA)
α = 1.66±0.30
s_min = 56508
β = 1.27±0.10
Ouagadougou
Bobo-Dioulasso
Koudougou
Fada N'Gourma
Bangladesh (BGD)
α = 1.27±0.32
s_min = 1035698
β = 1.20±0.08
Dhaka
Comilla
Chattogram
Sirajganj
Brahmanbaria
Belarus (BLR)
α = 0.95±0.26
s_min = 90409
β = 1.00±0.03
Minsk
Homel
Vitebsk
Mahilyow
Hrodna
Brest
Bolivia (BOL)
α = 0.80±0.24
s_min = 84248
β = 1.07±0.08
El Alto [La Paz]
Santa Cruz de la Sierra
Cochabamba
Oruro
Sucre
Tarija
Potosi

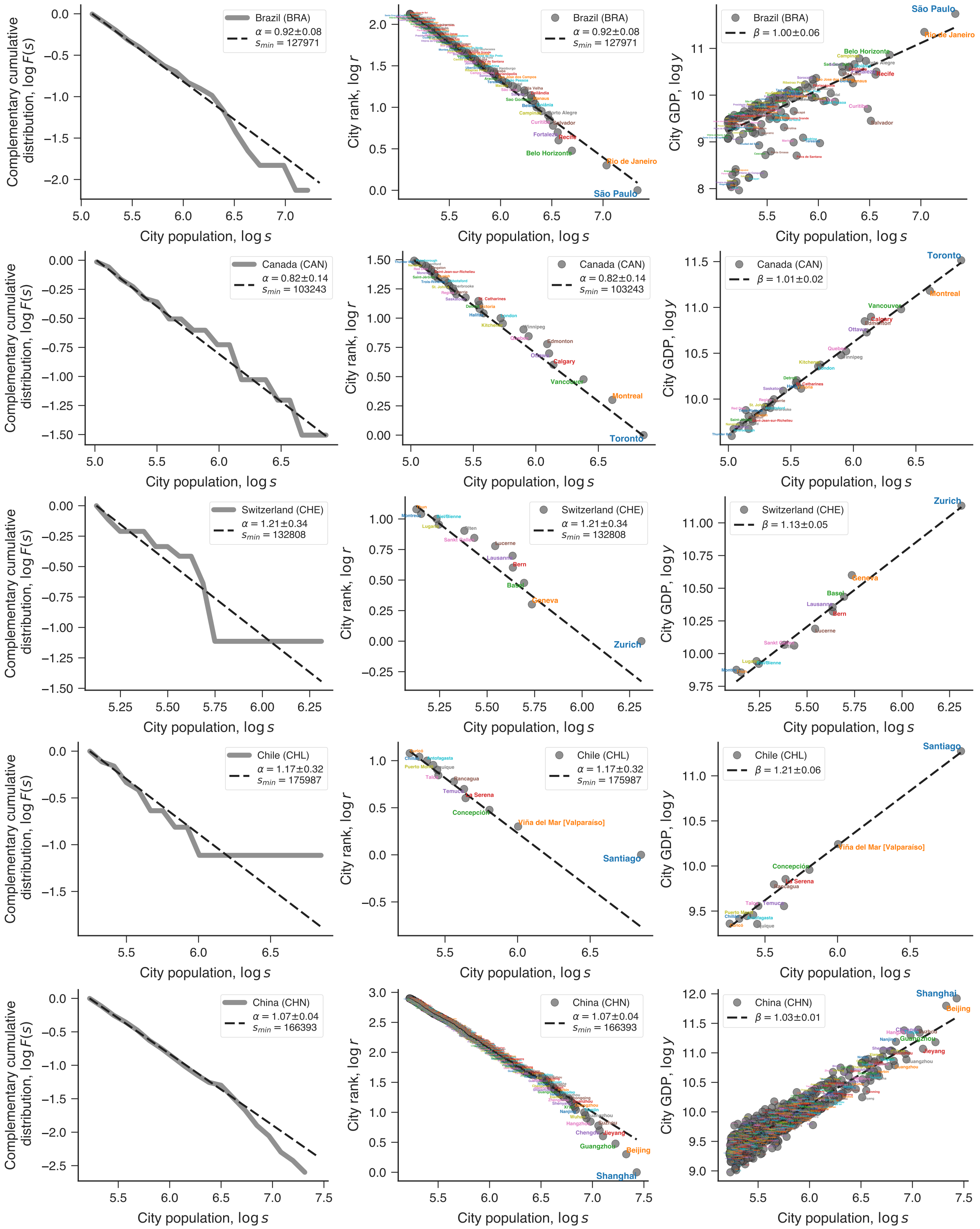
Complementary cumulative distribution, log $F(s)$
City rank, log r
City GDP, log y
City population, log s
Brazil (BRA)
$\alpha = 0.92 \pm 0.08$
s_{min} = 127971
$\beta = 1.00 \pm 0.06$
São Paulo
Rio de Janeiro
Belo Horizonte
Recife
Fortaleza
Salvador
Curitiba
Porto Alegre
Canada (CAN)
$\alpha = 0.82 \pm 0.14$
s_{min} = 103243
$\beta = 1.01 \pm 0.02$
Toronto
Montreal
Vancouver
Calgary
Edmonton
Ottawa
Winnipeg
Quebec
London
Kitchener
Halifax
Victoria
St. Catharines
Switzerland (CHE)
$\alpha = 1.21 \pm 0.34$
s_{min} = 132808
$\beta = 1.13 \pm 0.05$
Zurich
Geneva
Basel
Bern
Lausanne
Lucerne
Sankt Gallen
Lugano
Biel/Bienne
Montreux
Winterthur
Chile (CHL)
$\alpha = 1.17 \pm 0.32$
s_{min} = 175987
$\beta = 1.21 \pm 0.06$
Santiago
Viña del Mar [Valparaíso]
Concepción
La Serena
Temuco
Rancagua
Talca
Puerto Montt
Antofagasta
Iquique
Chillán
China (CHN)
$\alpha = 1.07 \pm 0.04$
s_{min} = 166393
$\beta = 1.03 \pm 0.01$
Shanghai
Beijing
Guangzhou
Jieyang
Chengdu
Hangzhou
Nanjing
Wuhan
Suzhou

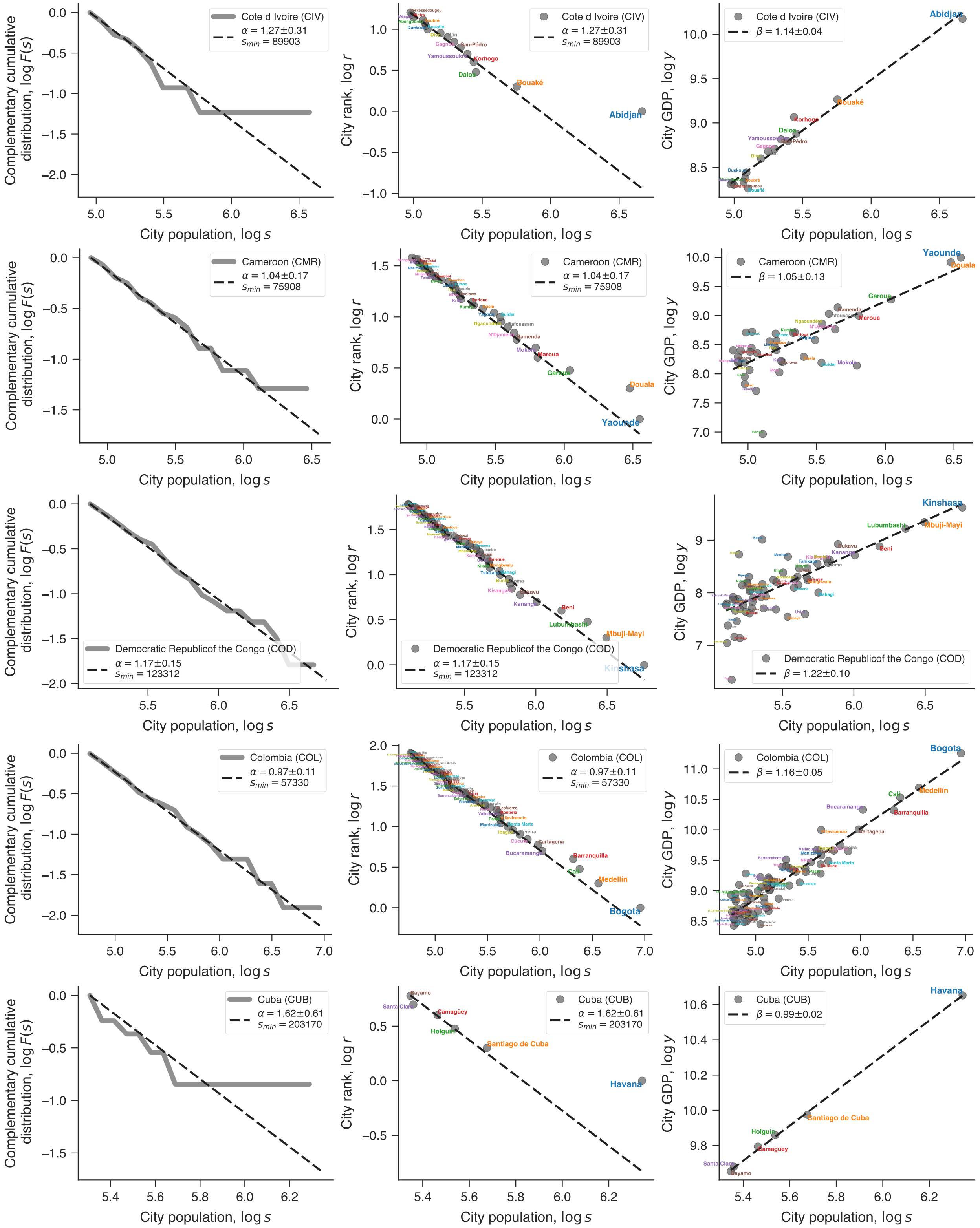
Complementary cumulative distribution, log F(s)
City rank, log r
City GDP, log y
City population, log s
Cote d Ivoire (CIV)
α = 1.27±0.31
s_min = 89903
β = 1.14±0.04
Abidjan
Bouaké
Korhogo
Daloa
Yamoussoukro
San-Pédro
Cameroon (CMR)
α = 1.04±0.17
s_min = 75908
β = 1.05±0.13
Yaounde
Douala
Garoua
Maroua
Bamenda
Democratic Republicof the Congo (COD)
α = 1.17±0.15
s_min = 123312
β = 1.22±0.10
Kinshasa
Mbuji-Mayi
Lubumbashi
Beni
Kananga
Bukavu
Colombia (COL)
α = 0.97±0.11
s_min = 57330
β = 1.16±0.05
Bogota
Medellin
Cali
Barranquilla
Cartagena
Bucaramanga
Cuba (CUB)
α = 1.62±0.61
s_min = 203170
β = 0.99±0.02
Havana
Santiago de Cuba
Holguin
Camagüey
Santa Clara
Bayamo

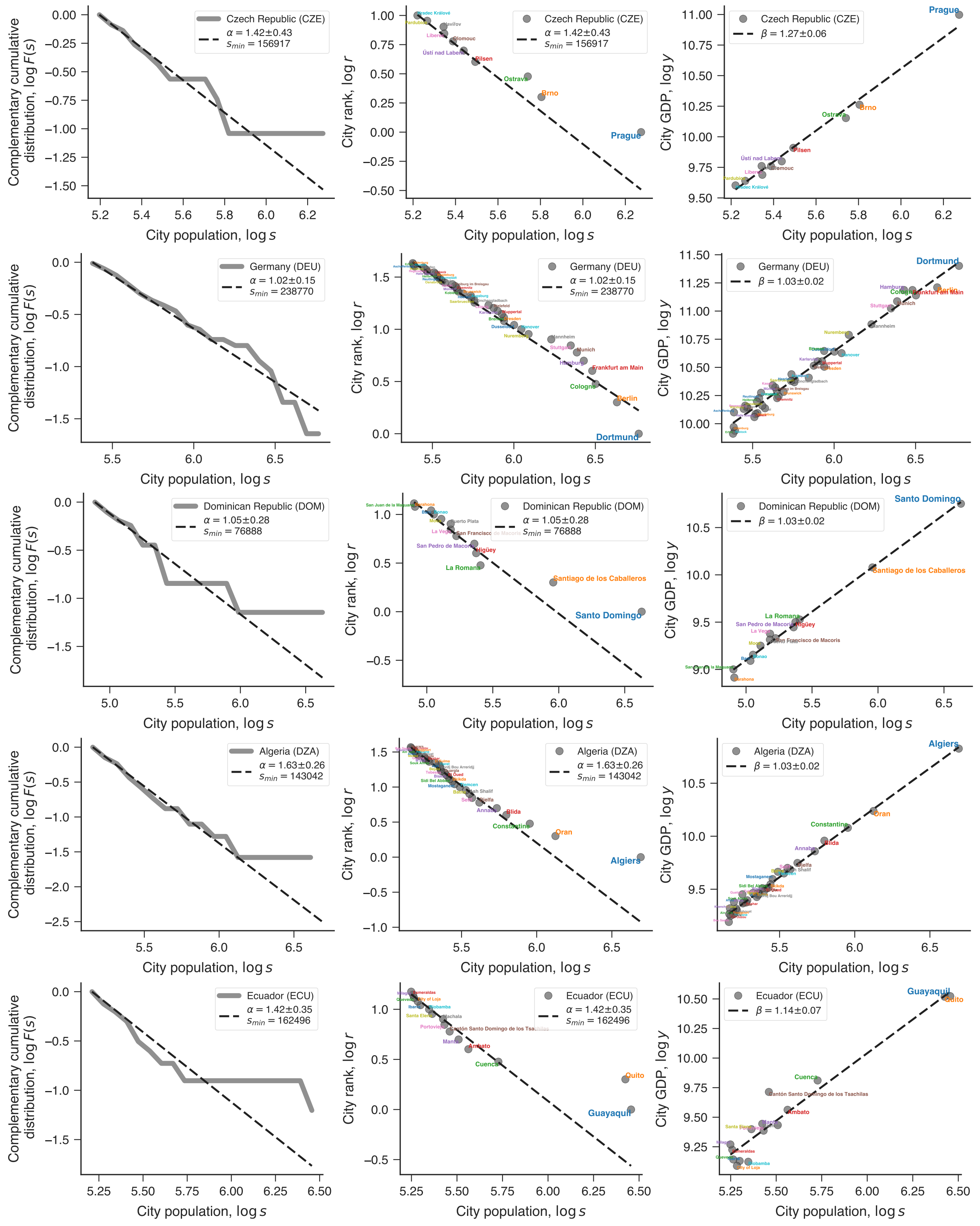
Complementary cumulative distribution, log F(s)
City rank, log r
City GDP, log y
City population, log s
Czech Republic (CZE)
α = 1.42±0.43
s_min = 156917
β = 1.27±0.06
Prague
Brno
Ostrava
Plzeň
Olomouc
Liberec
Ústí nad Labem
Germany (DEU)
α = 1.02±0.15
s_min = 238770
β = 1.03±0.02
Dortmund
Berlin
Cologne
Frankfurt am Main
Hamburg
Munich
Stuttgart
Mannheim
Nuremberg
Hanover
Dominican Republic (DOM)
α = 1.05±0.28
s_min = 76888
β = 1.03±0.02
Santo Domingo
Santiago de los Caballeros
La Romana
San Pedro de Macorís
Higüey
La Vega
San Francisco de Macorís
Puerto Plata
Algeria (DZA)
α = 1.63±0.26
s_min = 143042
β = 1.03±0.02
Algiers
Oran
Constantine
Blida
Annaba
Chlef
Tlemcen
Mostaganem
Ecuador (ECU)
α = 1.42±0.35
s_min = 162496
β = 1.14±0.07
Guayaquil
Quito
Cuenca
Ambato
Manta
Portoviejo
Santa Elena
Cantón Santo Domingo de los Tsachilas

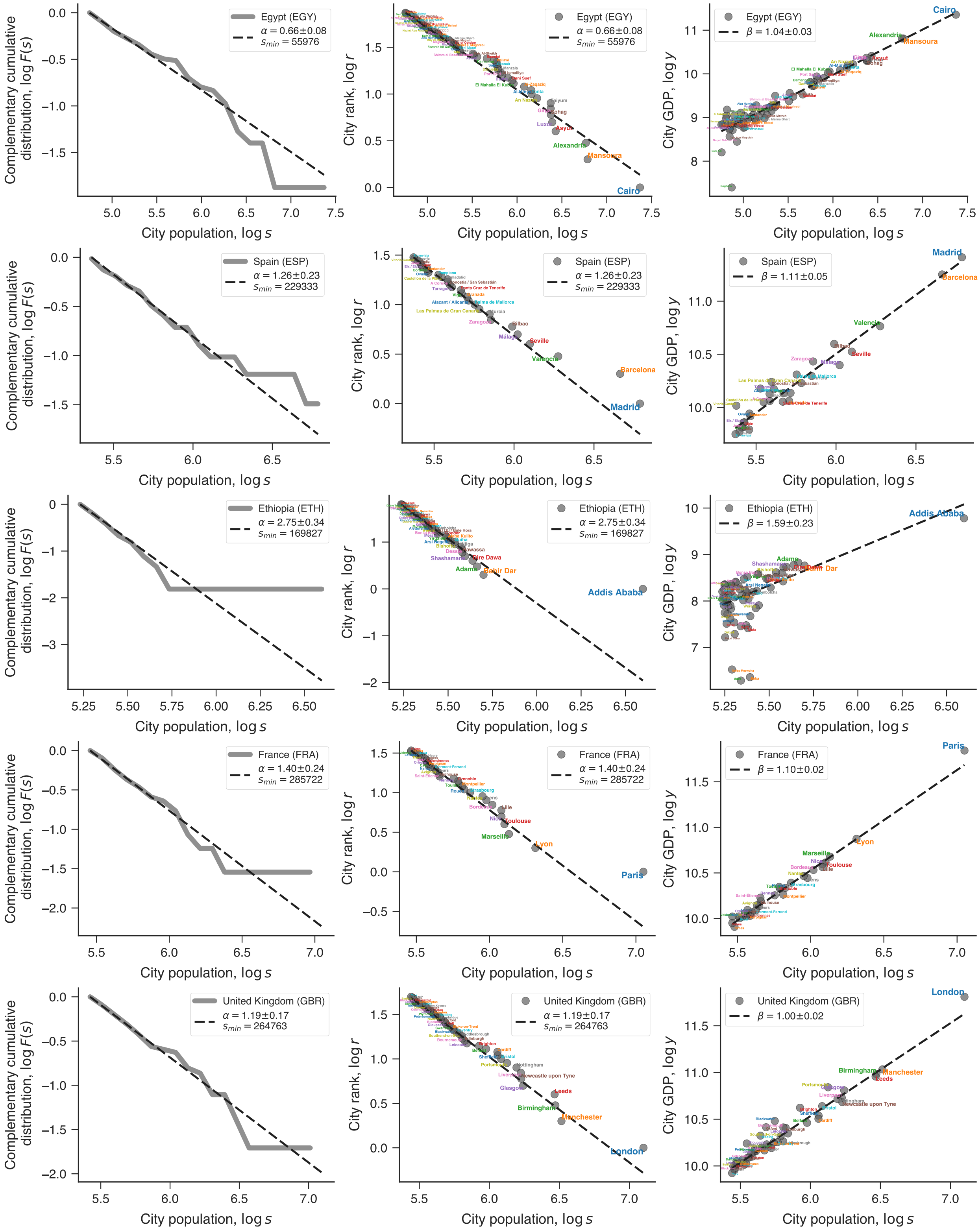
Complementary cumulative distribution, log F(s)
City rank, log r
City GDP, log y
City population, log s
Egypt (EGY)
α = 0.66±0.08
s_min = 55976
β = 1.04±0.03
Cairo
Alexandria
Mansoura
Spain (ESP)
α = 1.26±0.23
s_min = 229333
β = 1.11±0.05
Madrid
Barcelona
Valencia
Seville
Bilbao
Zaragoza
Málaga
Ethiopia (ETH)
α = 2.75±0.34
s_min = 169827
β = 1.59±0.23
Addis Ababa
Bahir Dar
Adama
Dire Dawa
Shashamane
France (FRA)
α = 1.40±0.24
s_min = 285722
β = 1.10±0.02
Paris
Lyon
Marseille
Toulouse
Lille
Nice
United Kingdom (GBR)
α = 1.19±0.17
s_min = 264763
β = 1.00±0.02
London
Manchester
Birmingham
Leeds
Glasgow
Newcastle upon Tyne
Nottingham

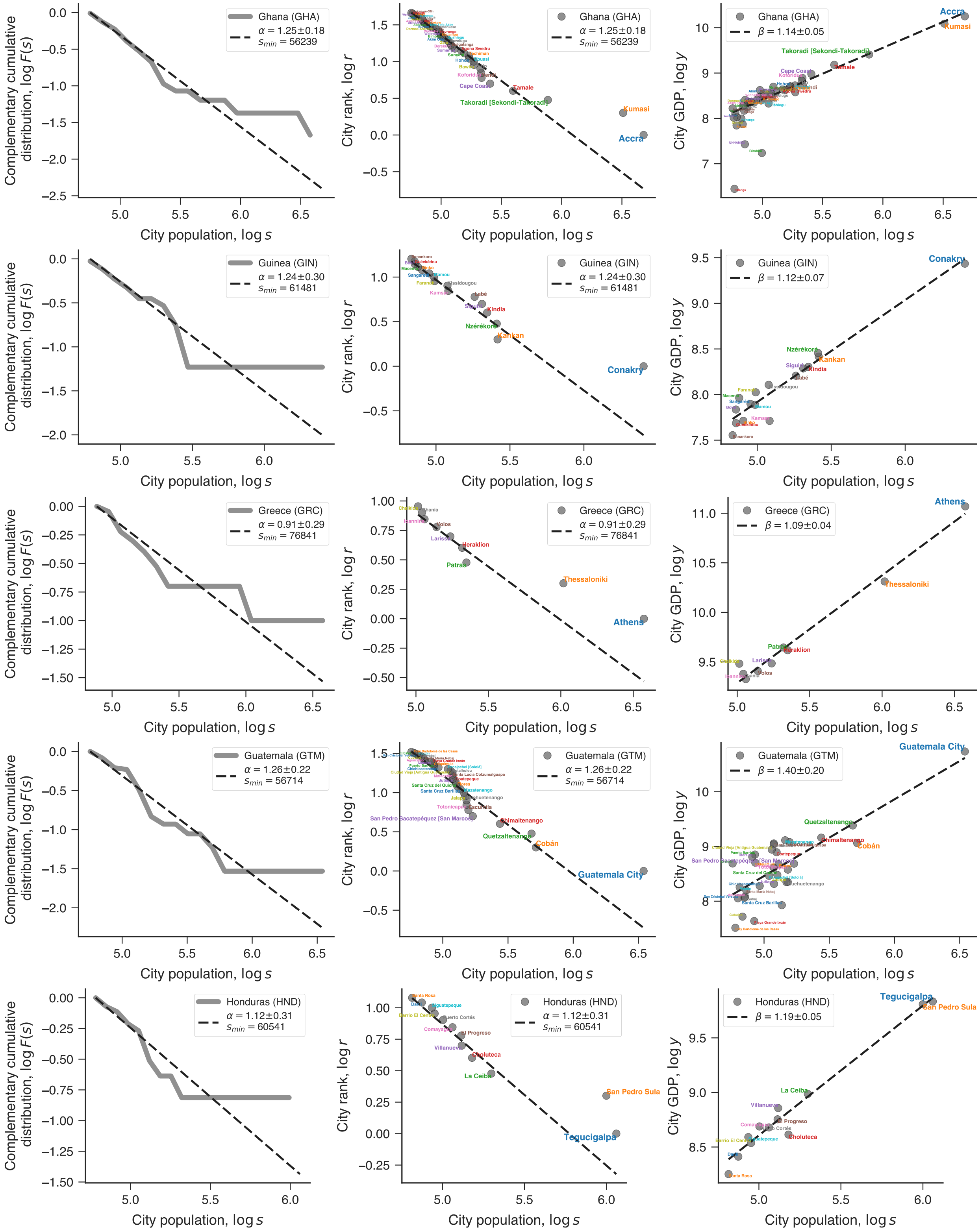
Complementary cumulative distribution, log $F(s)$
City rank, log r
City GDP, log y
City population, log s
Ghana (GHA)
$\alpha = 1.25 \pm 0.18$
$s_{min} = 56239$
$\beta = 1.14 \pm 0.05$
Accra
Kumasi
Tamale
Takoradi [Sekondi-Takoradi]
Cape Coast
Guinea (GIN)
$\alpha = 1.24 \pm 0.30$
$s_{min} = 61481$
$\beta = 1.12 \pm 0.07$
Conakry
Kankan
Nzérékoré
Kindia
Labé
Siguiri
Kamsar
Greece (GRC)
$\alpha = 0.91 \pm 0.29$
$s_{min} = 76841$
$\beta = 1.09 \pm 0.04$
Athens
Thessaloniki
Heraklion
Patras
Larisa
Volos
Chania
Ioannina
Guatemala (GTM)
$\alpha = 1.26 \pm 0.22$
$s_{min} = 56714$
$\beta = 1.40 \pm 0.20$
Guatemala City
Cobán
Quetzaltenango
Chimaltenango
San Pedro Sacatepéquez [San Marcos]
Totonicapán
Escuintla
Mazatenango
Santa Cruz Barillas
Honduras (HND)
$\alpha = 1.12 \pm 0.31$
$s_{min} = 60541$
$\beta = 1.19 \pm 0.05$
Tegucigalpa
San Pedro Sula
La Ceiba
Choluteca
Villanueva
El Progreso
Comayagua
Puerto Cortés
Siguatepeque
Danlí
Santa Rosa

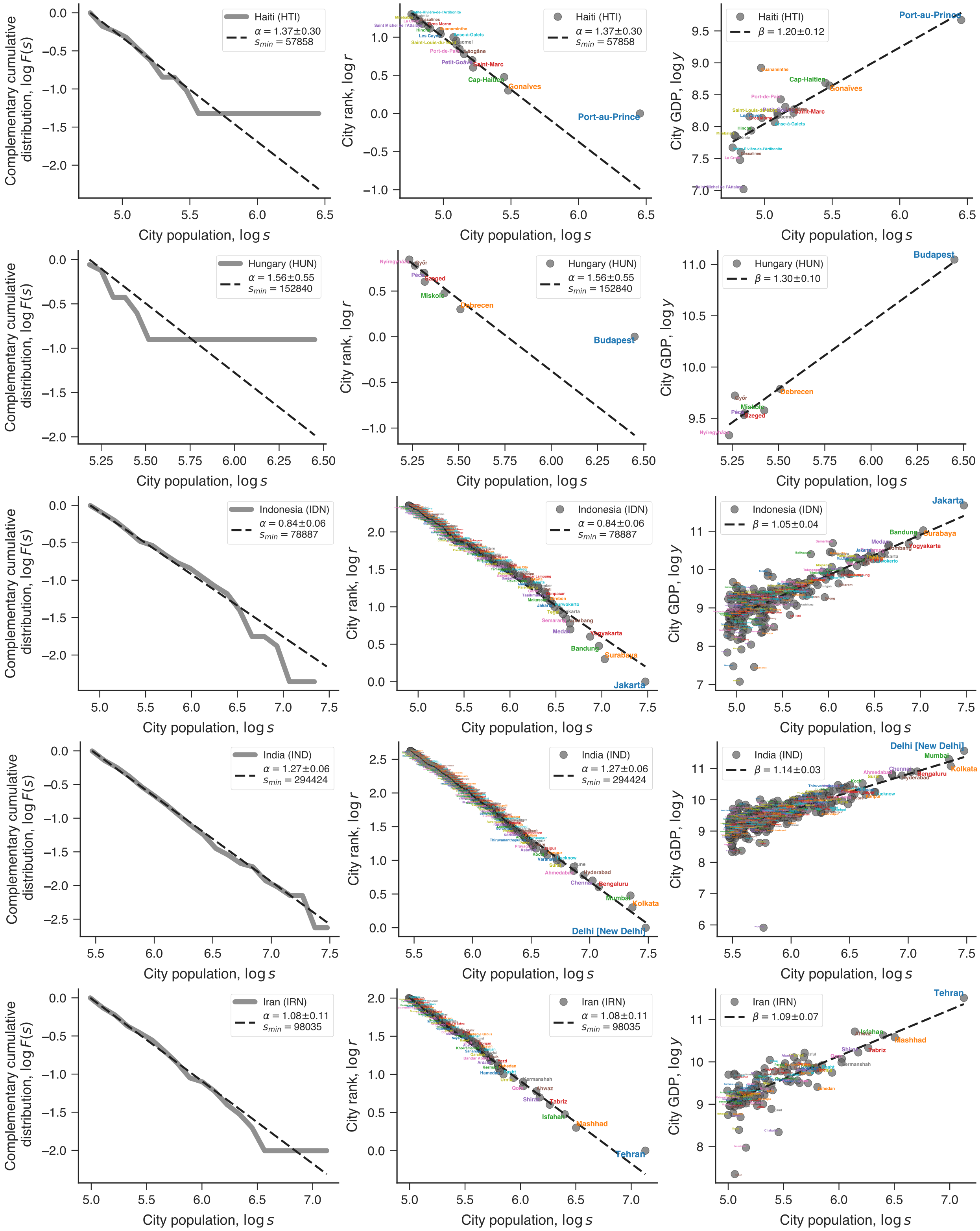
Complementary cumulative distribution, log F(s)
City rank, log r
City GDP, log y
City population, log s
Haiti (HTI)
α = 1.37±0.30
s_min = 57858
β = 1.20±0.12
Port-au-Prince
Gonaïves
Cap-Haïtien
Saint-Marc
Hungary (HUN)
α = 1.56±0.55
s_min = 152840
β = 1.30±0.10
Budapest
Debrecen
Miskolc
Szeged
Győr
Indonesia (IDN)
α = 0.84±0.06
s_min = 78887
β = 1.05±0.04
Jakarta
Surabaya
Bandung
Medan
Yogyakarta
India (IND)
α = 1.27±0.06
s_min = 294424
β = 1.14±0.03
Delhi [New Delhi]
Mumbai
Kolkata
Bengaluru
Chennai
Hyderabad
Iran (IRN)
α = 1.08±0.11
s_min = 98035
β = 1.09±0.07
Tehran
Mashhad
Isfahan
Tabriz
Shiraz

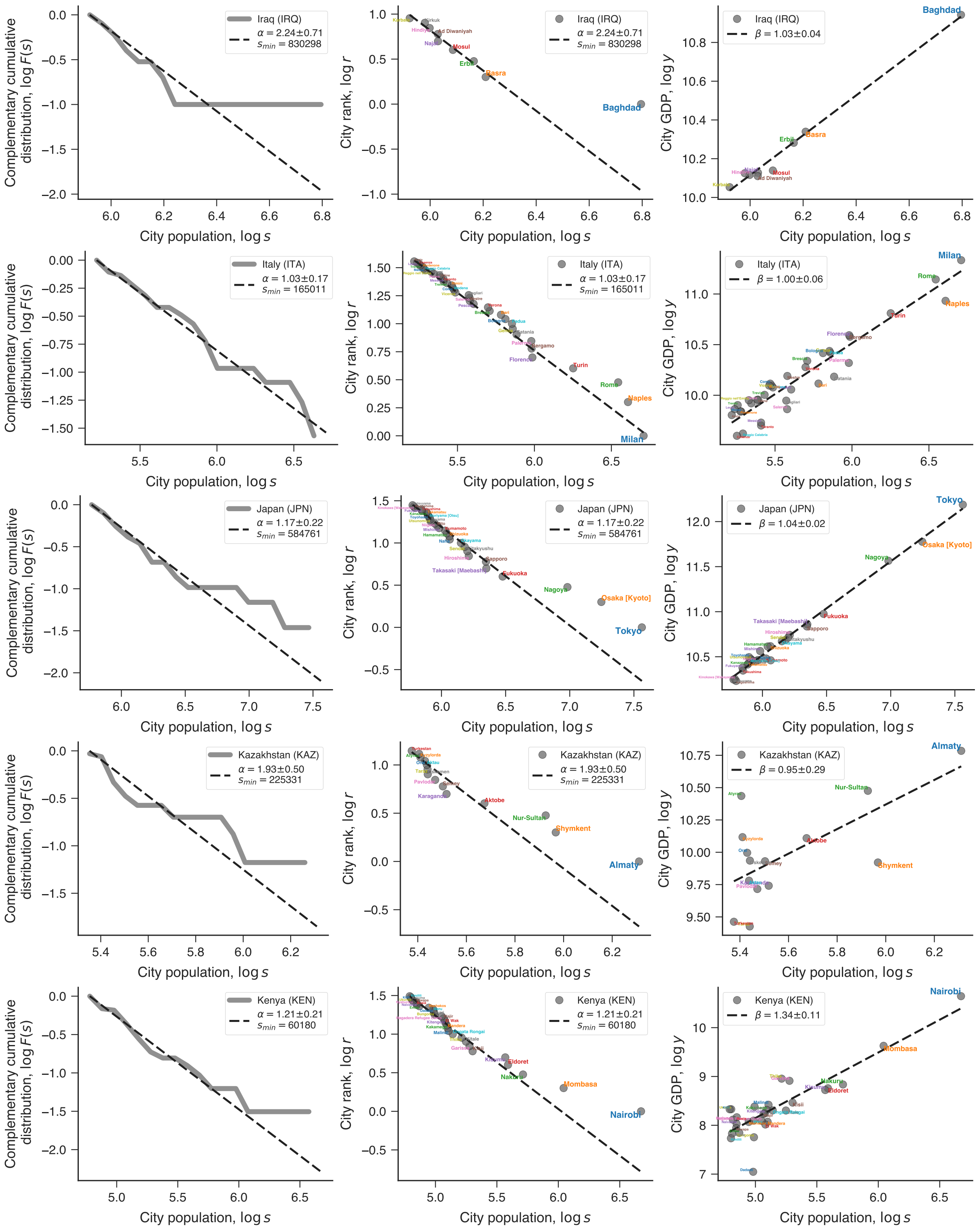

Complementary cumulative distribution, log F(s)
City rank, log r
City GDP, log y
City population, log s
Iraq (IRQ)
α = 2.24±0.71
s_min = 830298
β = 1.03±0.04
Baghdad
Basra
Erbil
Mosul
Najaf
Kirkuk
Hindiya
Ad Diwaniyah
Italy (ITA)
α = 1.03±0.17
s_min = 165011
β = 1.00±0.06
Milan
Rome
Naples
Turin
Florence
Bergamo
Verona
Bari
Padua
Catania
Palermo
Brescia
Bologna
Japan (JPN)
α = 1.17±0.22
s_min = 584761
β = 1.04±0.02
Tokyo
Osaka [Kyoto]
Nagoya
Fukuoka
Sapporo
Takasaki [Maebashi]
Hiroshima
Kitakyushu
Sendai
Okayama
Hamamatsu
Kumamoto
Kazakhstan (KAZ)
α = 1.93±0.50
s_min = 225331
β = 0.95±0.29
Almaty
Shymkent
Nur-Sultan
Aktobe
Karaganda
Pavlodar
Atyrau
Kyzylorda
Semey
Kenya (KEN)
α = 1.21±0.21
s_min = 60180
β = 1.34±0.11
Nairobi
Mombasa
Nakuru
Eldoret
Kisumu
Garissa
Dadaab

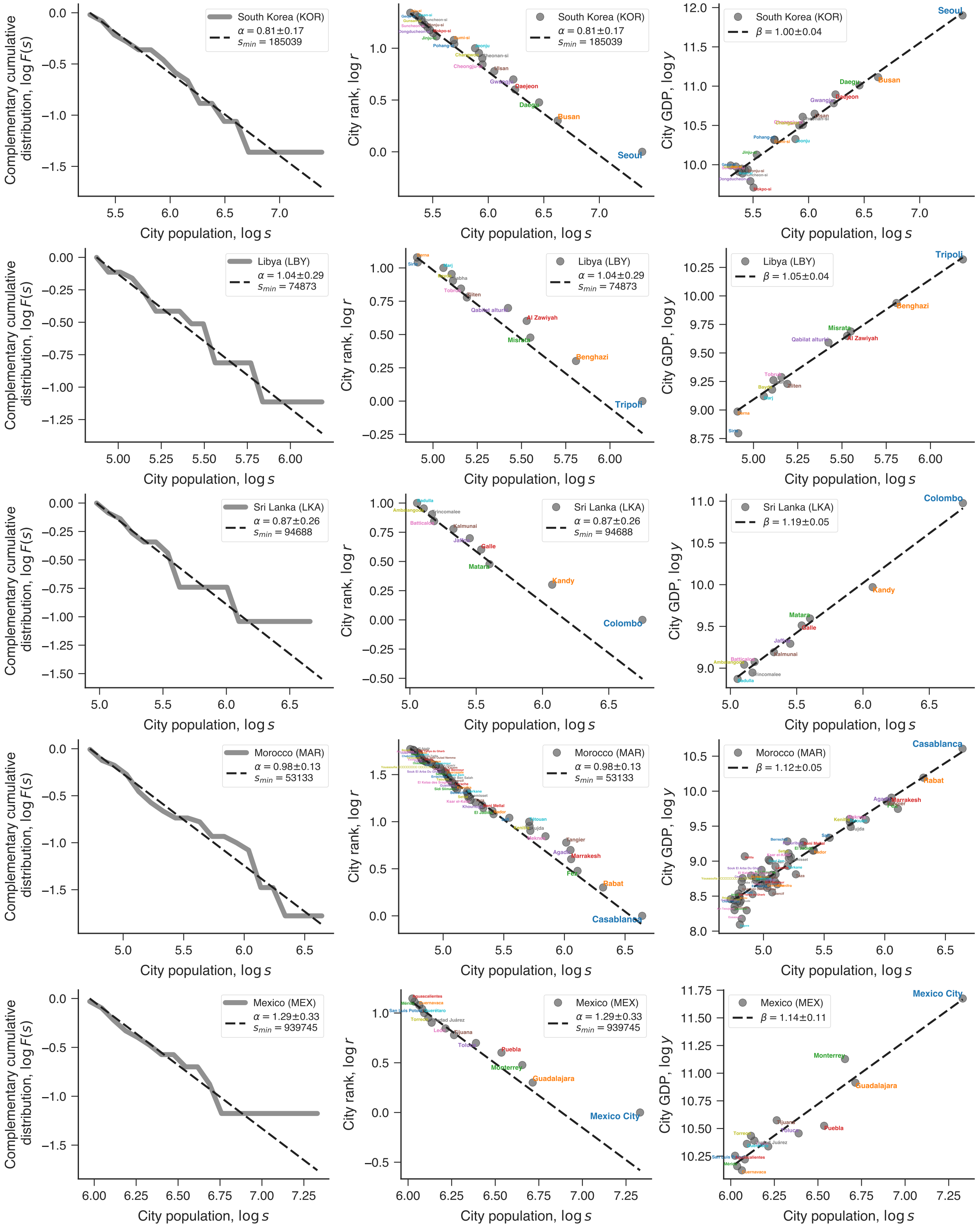
Complementary cumulative distribution, log F(s)
City rank, log r
City GDP, log y
City population, log s
South Korea (KOR)
α = 0.81±0.17
s_min = 185039
β = 1.00±0.04
Seoul
Busan
Daegu
Daejeon
Libya (LBY)
α = 1.04±0.29
s_min = 74873
β = 1.05±0.04
Tripoli
Benghazi
Misrata
Al Zawiyah
Sri Lanka (LKA)
α = 0.87±0.26
s_min = 94688
β = 1.19±0.05
Colombo
Kandy
Galle
Matara
Morocco (MAR)
α = 0.98±0.13
s_min = 53133
β = 1.12±0.05
Casablanca
Rabat
Fes
Marrakesh
Tangier
Mexico (MEX)
α = 1.29±0.33
s_min = 939745
β = 1.14±0.11
Mexico City
Guadalajara
Monterrey
Puebla
Tijuana
Toluca

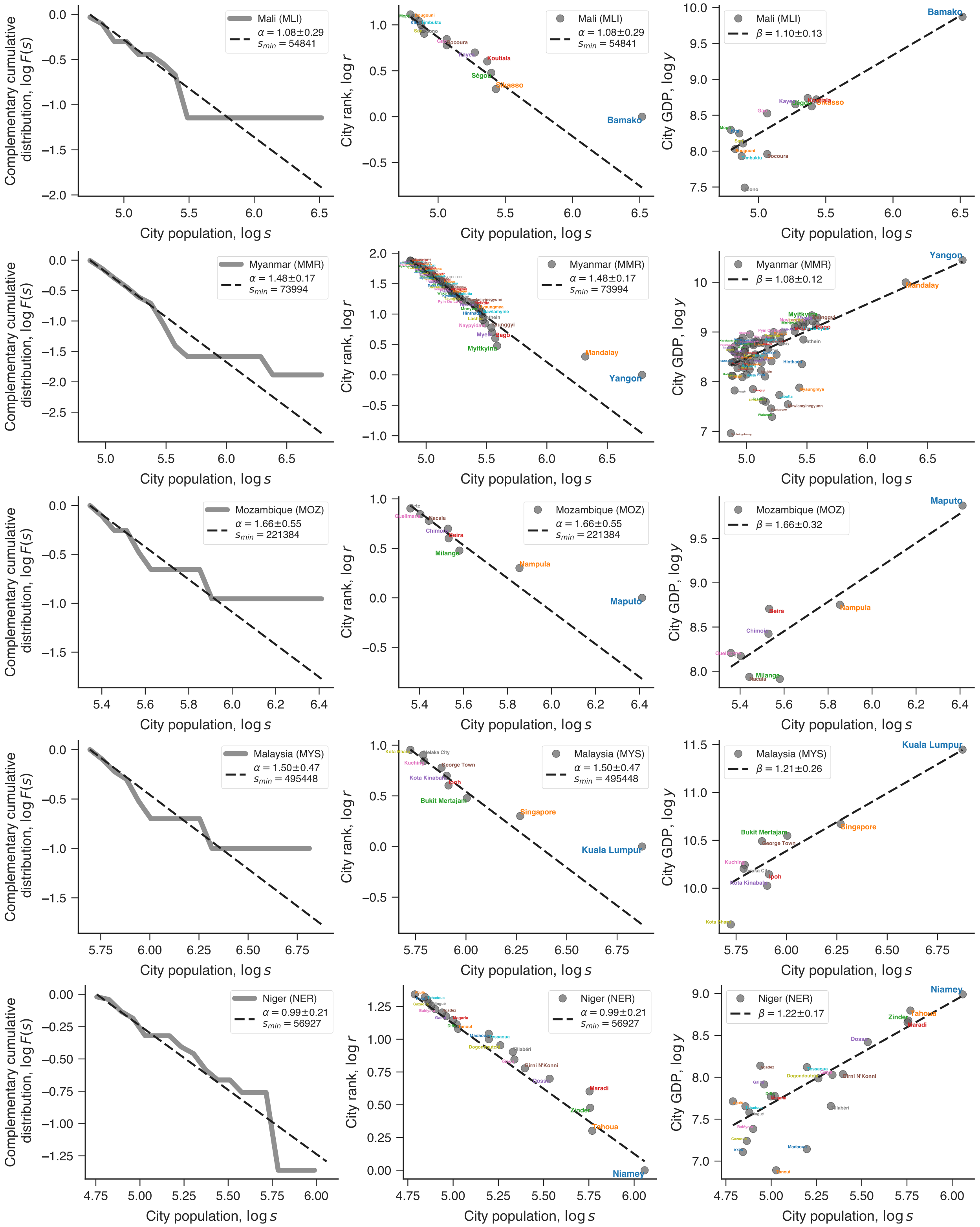
Complementary cumulative distribution, log F(s)
City population, log s
City rank, log r
City GDP, log y
Mali (MLI)
α = 1.08±0.29
s_min = 54841
β = 1.10±0.13
Bamako
Koutiala
Ségou
Sikasso
Kayes
Gao
Koutiala
Myanmar (MMR)
α = 1.48±0.17
s_min = 73994
β = 1.08±0.12
Yangon
Mandalay
Myitkyina
Hinthada
Mozambique (MOZ)
α = 1.66±0.55
s_min = 221384
β = 1.66±0.32
Maputo
Nampula
Beira
Milange
Chimoio
Nacala
Malaysia (MYS)
α = 1.50±0.47
s_min = 495448
β = 1.21±0.26
Kuala Lumpur
Singapore
Bukit Mertajam
George Town
Kota Kinabalu
Ipoh
Kuching
Malaka City
Niger (NER)
α = 0.99±0.21
s_min = 56927
β = 1.22±0.17
Niamey
Zinder
Maradi
Tahoua
Dosso
Birni N'Konni
Madaoua
Tillabéri
Dogondoutchi

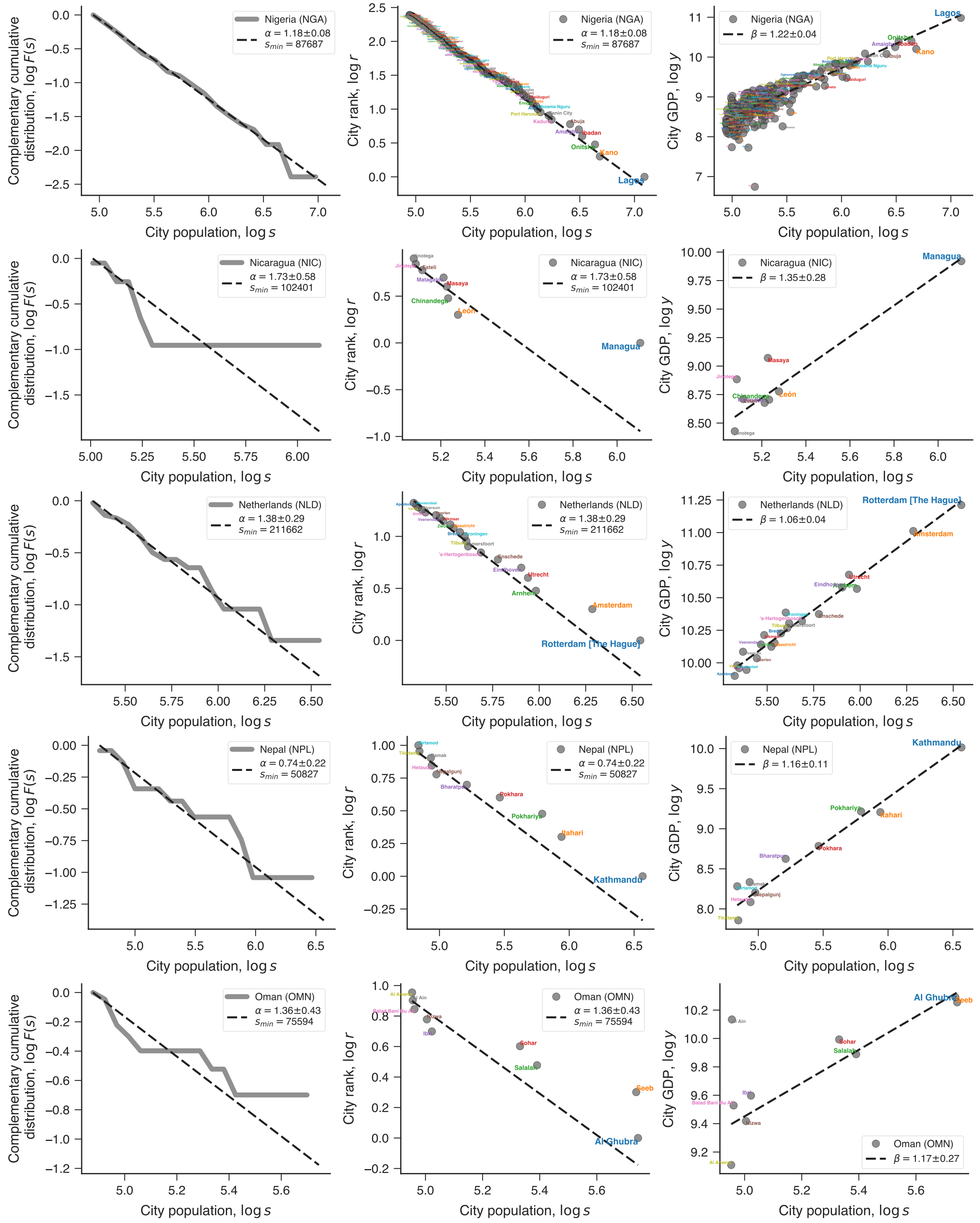
Complementary cumulative distribution, log F(s)
City rank, log r
City GDP, log y
City population, log s
Nigeria (NGA)
α = 1.18±0.08
s_min = 87687
β = 1.22±0.04
Lagos
Kano
Ibadan
Abuja
Onitsha
Nicaragua (NIC)
α = 1.73±0.58
s_min = 102401
β = 1.35±0.28
Managua
Masaya
León
Chinandega
Netherlands (NLD)
α = 1.38±0.29
s_min = 211662
β = 1.06±0.04
Rotterdam [The Hague]
Amsterdam
Utrecht
Eindhoven
Arnhem
Enschede
Nepal (NPL)
α = 0.74±0.22
s_min = 50827
β = 1.16±0.11
Kathmandu
Pokhara
Pokhariya
Itahari
Bharatpur
Oman (OMN)
α = 1.36±0.43
s_min = 75594
β = 1.17±0.27
Al Ghubra
Seeb
Sohar
Salalah
Ibri

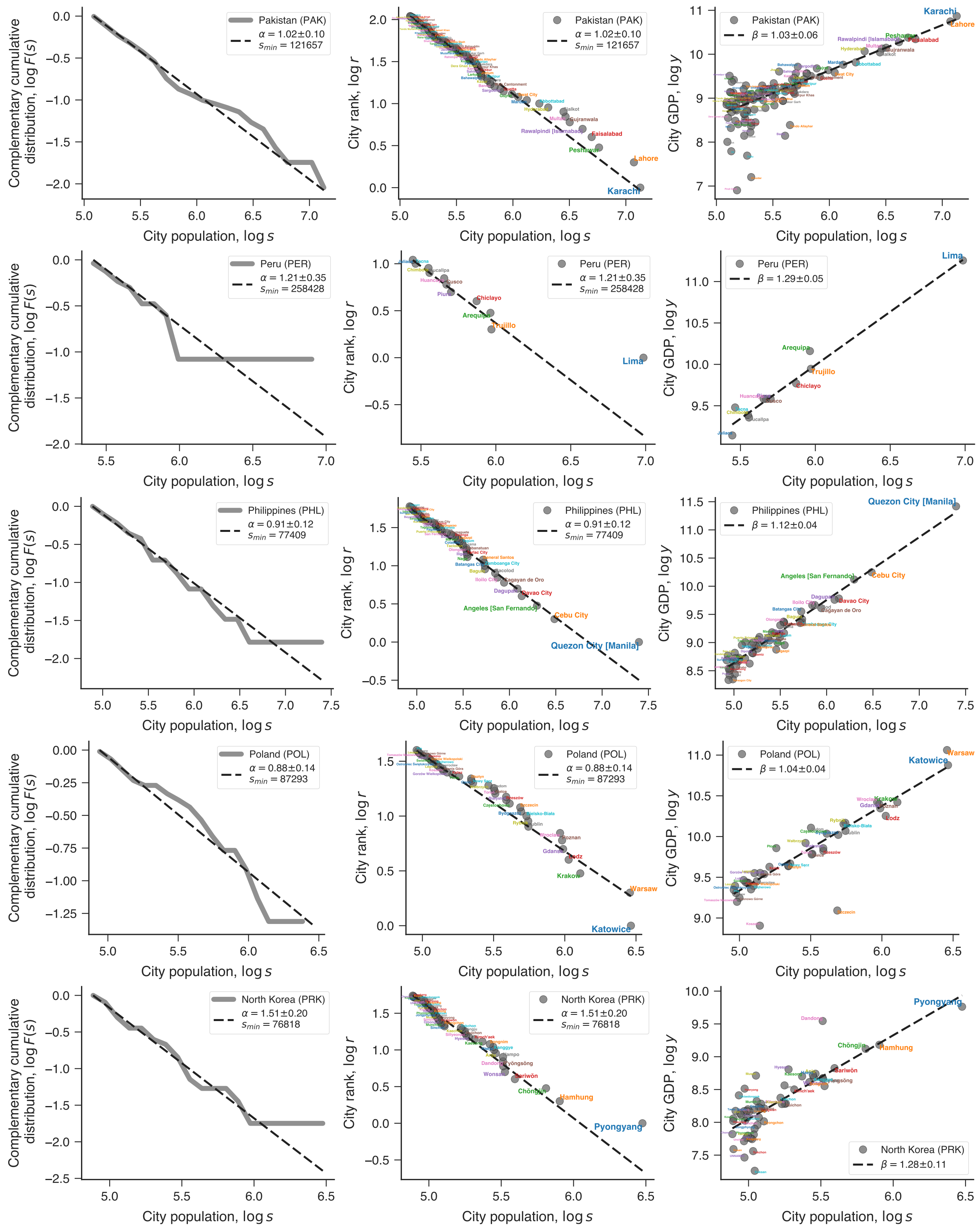
Complementary cumulative distribution, log F(s)
City rank, log r
City GDP, log y
City population, log s
Pakistan (PAK)
α = 1.02±0.10
s_min = 121657
β = 1.03±0.06
Karachi
Lahore
Faisalabad
Rawalpindi [Islamabad]
Peshawar
Gujranwala
Peru (PER)
α = 1.21±0.35
s_min = 258428
β = 1.29±0.05
Lima
Arequipa
Trujillo
Chiclayo
Philippines (PHL)
α = 0.91±0.12
s_min = 77409
β = 1.12±0.04
Quezon City [Manila]
Cebu City
Angeles [San Fernando]
Davao City
Cagayan de Oro
Poland (POL)
α = 0.88±0.14
s_min = 87293
β = 1.04±0.04
Warsaw
Katowice
Krakow
Lodz
Gdansk
Poznan
North Korea (PRK)
α = 1.51±0.20
s_min = 76818
β = 1.28±0.11
Pyongyang
Hamhung
Chŏngjin
Sariwŏn
Dandong

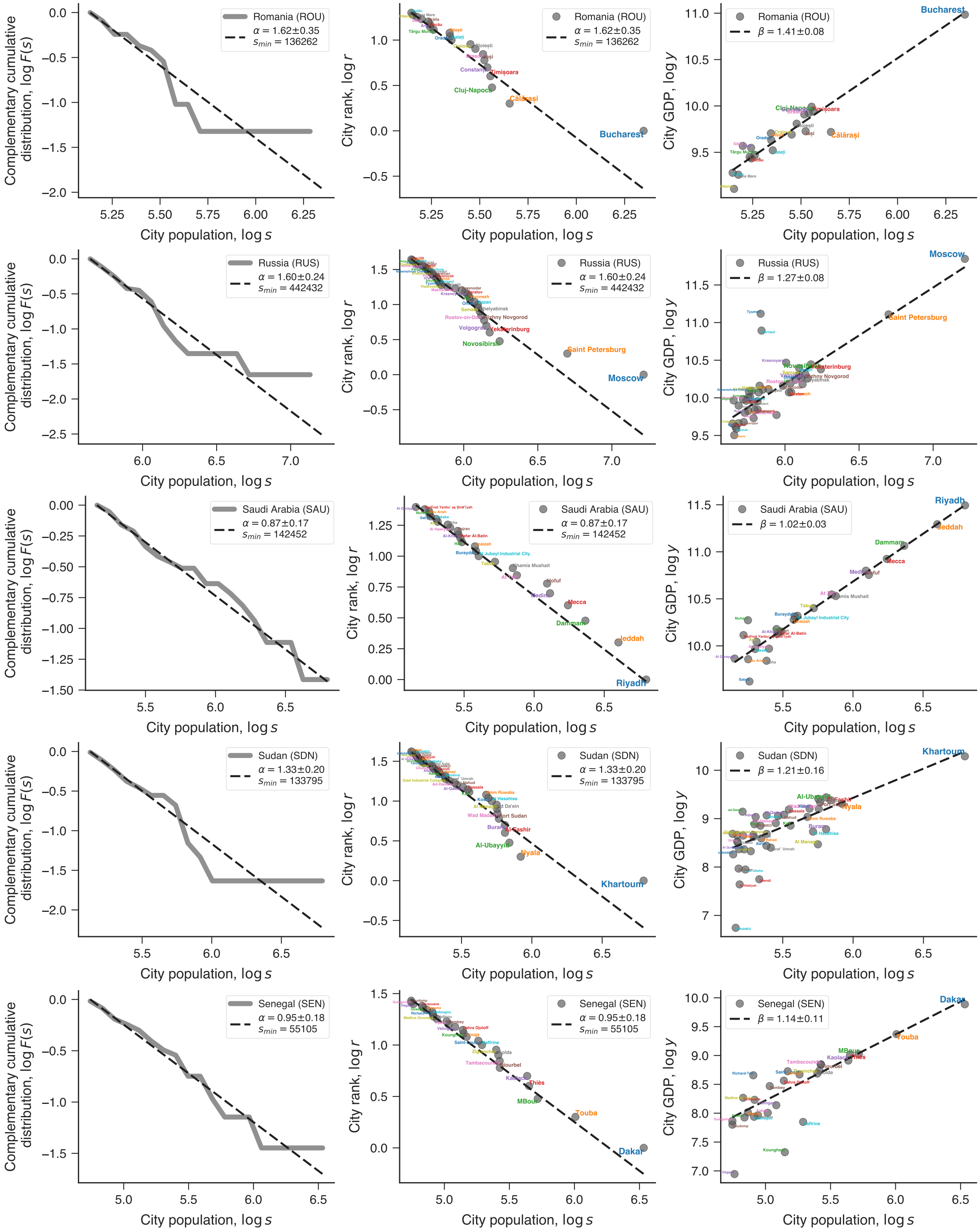

Complementary cumulative distribution, log F(s)
City rank, log r
City GDP, log y
City population, log s
Romania (ROU)
α = 1.62±0.35
s_min = 136262
β = 1.41±0.08
Bucharest
Cluj-Napoca
Timișoara
Călărași
Constanța
Russia (RUS)
α = 1.60±0.24
s_min = 442432
β = 1.27±0.08
Moscow
Saint Petersburg
Novosibirsk
Yekaterinburg
Nizhny Novgorod
Volgograd
Saudi Arabia (SAU)
α = 0.87±0.17
s_min = 142452
β = 1.02±0.03
Riyadh
Jeddah
Mecca
Dammam
Medina
Hofuf
Sudan (SDN)
α = 1.33±0.20
s_min = 133795
β = 1.21±0.16
Khartoum
Nyala
Al-Ubayyid
Port Sudan
Senegal (SEN)
α = 0.95±0.18
s_min = 55105
β = 1.14±0.11
Dakar
Touba
MBour
Thiès
Kaolack

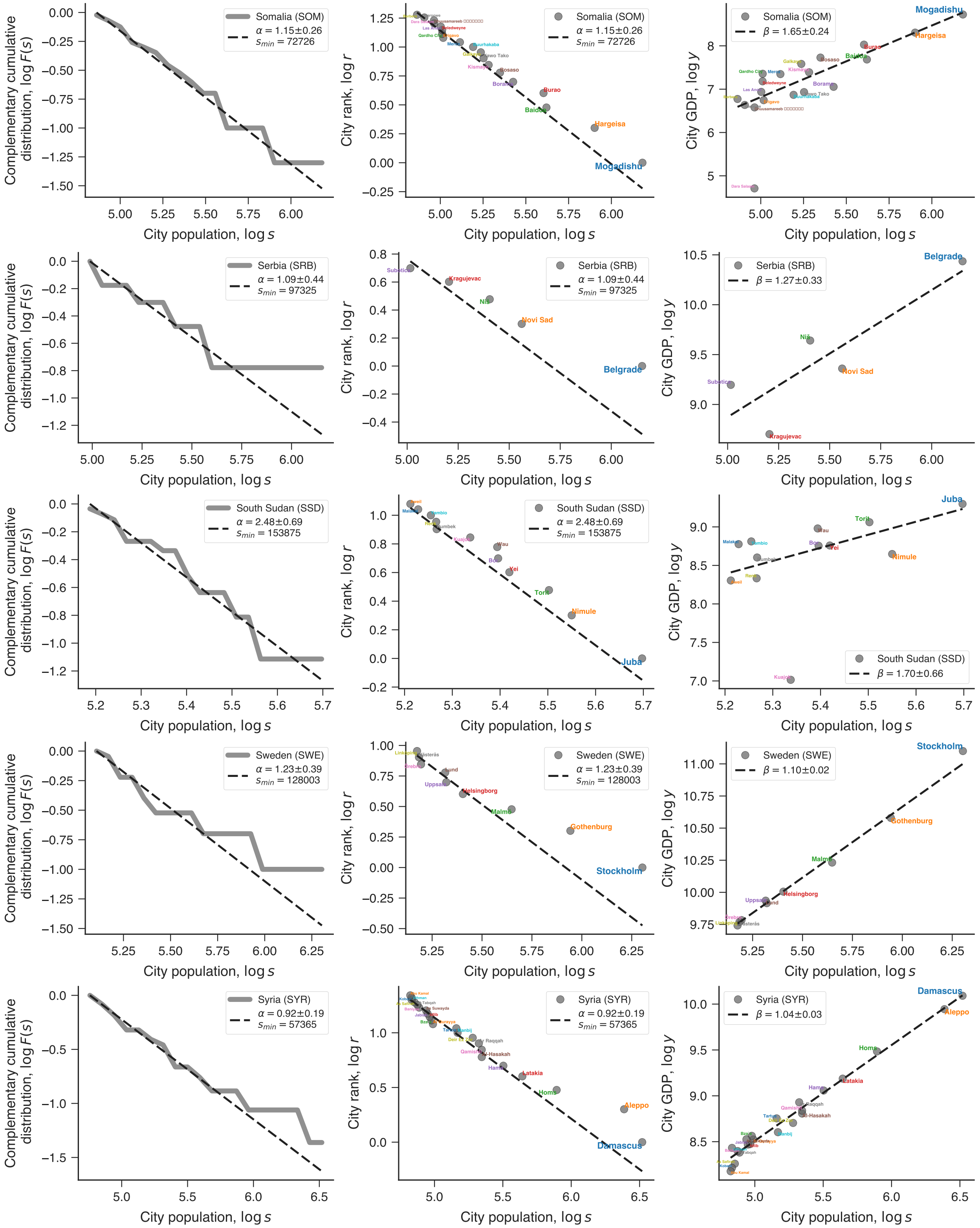
Complementary cumulative distribution, log F(s)
City rank, log r
City GDP, log y
City population, log s
Somalia (SOM)
α = 1.15±0.26
s_min = 72726
β = 1.65±0.24
Mogadishu
Hargeisa
Burao
Baidoa
Bosaso
Borama
Serbia (SRB)
α = 1.09±0.44
s_min = 97325
β = 1.27±0.33
Belgrade
Novi Sad
Niš
Kragujevac
Subotica
South Sudan (SSD)
α = 2.48±0.69
s_min = 153875
β = 1.70±0.66
Juba
Nimule
Torit
Yei
Wau
Sweden (SWE)
α = 1.23±0.39
s_min = 128003
β = 1.10±0.02
Stockholm
Gothenburg
Malmö
Helsingborg
Uppsala
Lund
Syria (SYR)
α = 0.92±0.19
s_min = 57365
β = 1.04±0.03
Damascus
Aleppo
Homs
Latakia
Hama
Al-Hasakah
Raqqah

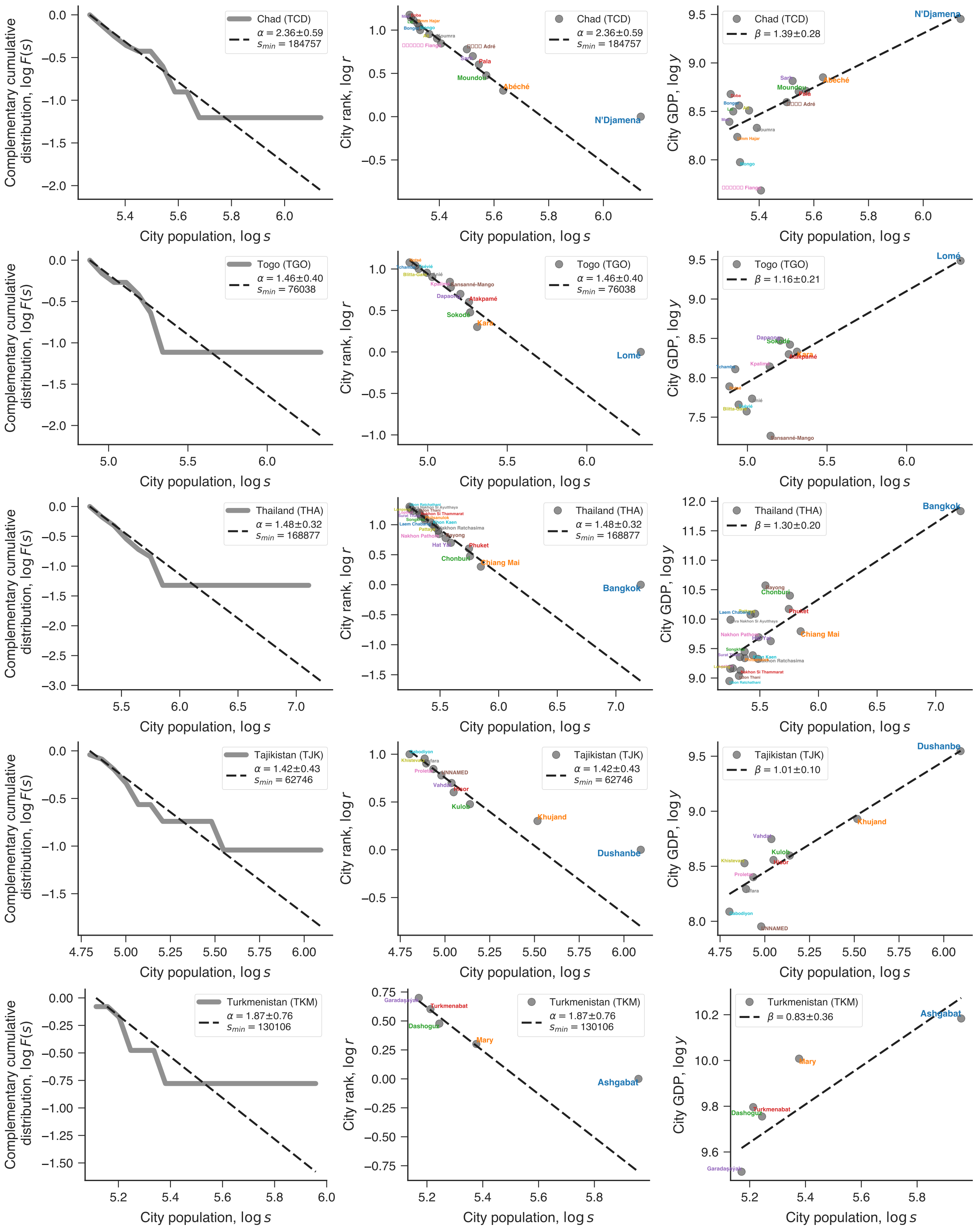
Complementary cumulative distribution, log $F(s)$
City rank, log r
City GDP, log y
City population, log s
Chad (TCD)
$\alpha = 2.36 \pm 0.59$
$s_{min} = 184757$
$\beta = 1.39 \pm 0.28$
N'Djamena
Abéché
Moundou
Sarh
Togo (TGO)
$\alpha = 1.46 \pm 0.40$
$s_{min} = 76038$
$\beta = 1.16 \pm 0.21$
Lomé
Kara
Sokodé
Atakpamé
Dapaong
Kpalimé
Thailand (THA)
$\alpha = 1.48 \pm 0.32$
$s_{min} = 168877$
$\beta = 1.30 \pm 0.20$
Bangkok
Chiang Mai
Chonburi
Phuket
Rayong
Tajikistan (TJK)
$\alpha = 1.42 \pm 0.43$
$s_{min} = 62746$
$\beta = 1.01 \pm 0.10$
Dushanbe
Khujand
Kulob
Vahdat
Turkmenistan (TKM)
$\alpha = 1.87 \pm 0.76$
$s_{min} = 130106$
$\beta = 0.83 \pm 0.36$
Ashgabat
Mary
Dashoguz
Turkmenabat
Garadaşaýak

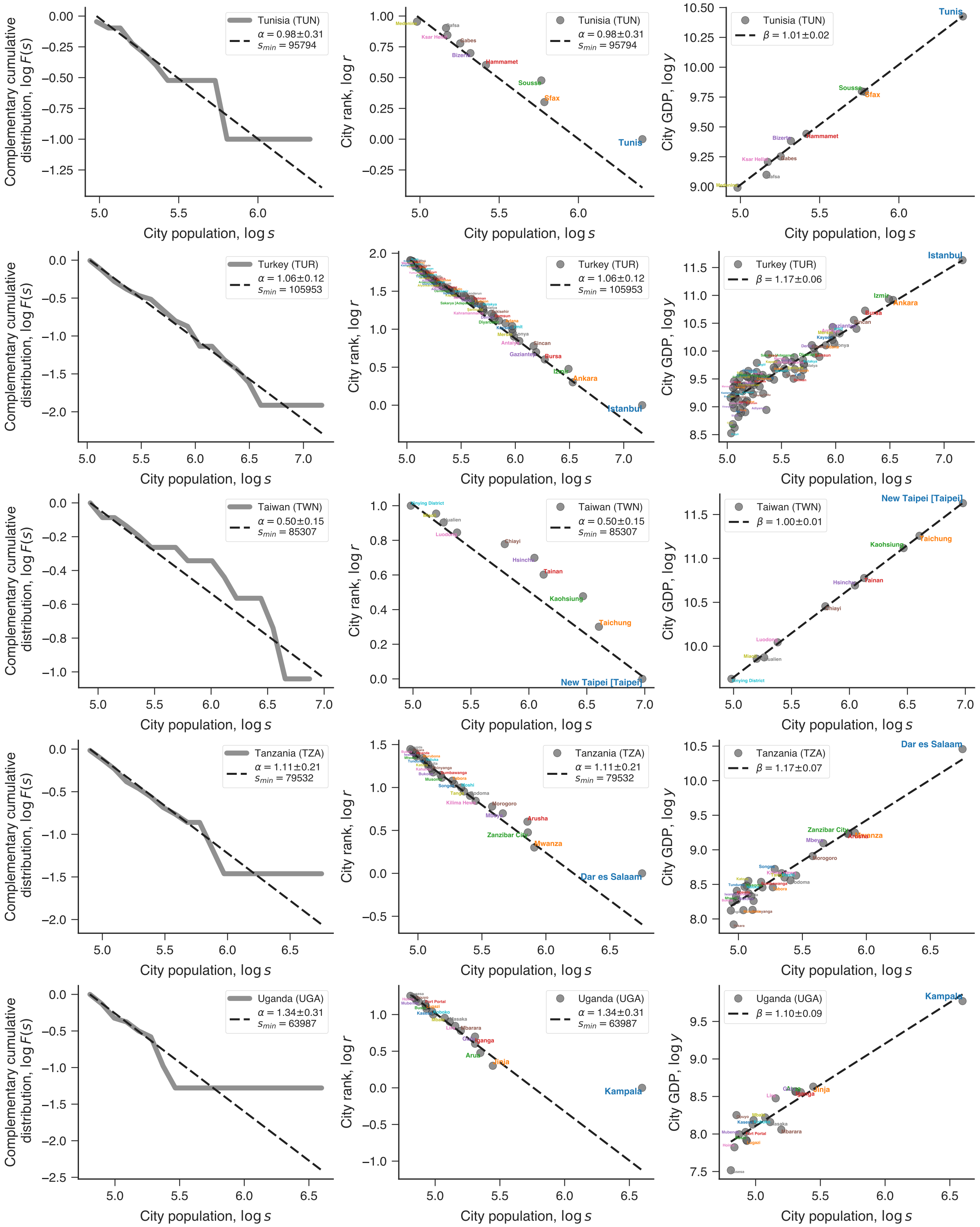
Complementary cumulative distribution, log F(s)
City rank, log r
City GDP, log y
City population, log s
Tunisia (TUN)
α = 0.98±0.31
s_min = 95794
β = 1.01±0.02
Tunis
Sfax
Sousse
Hammamet
Bizerte
Gabes
Gafsa
Turkey (TUR)
α = 1.06±0.12
s_min = 105953
β = 1.17±0.06
Istanbul
Ankara
Izmir
Bursa
Sincan
Gaziantep
Konya
Antalya
Taiwan (TWN)
α = 0.50±0.15
s_min = 85307
β = 1.00±0.01
New Taipei [Taipei]
Taichung
Kaohsiung
Tainan
Hsinchu
Chiayi
Luodong
Hualien
Yingying District
Tanzania (TZA)
α = 1.11±0.21
s_min = 79532
β = 1.17±0.07
Dar es Salaam
Mwanza
Arusha
Zanzibar City
Mbeya
Morogoro
Dodoma
Uganda (UGA)
α = 1.34±0.31
s_min = 63987
β = 1.10±0.09
Kampala
Jinja
Gulu
Mbarara
Masaka
Arua
Lira

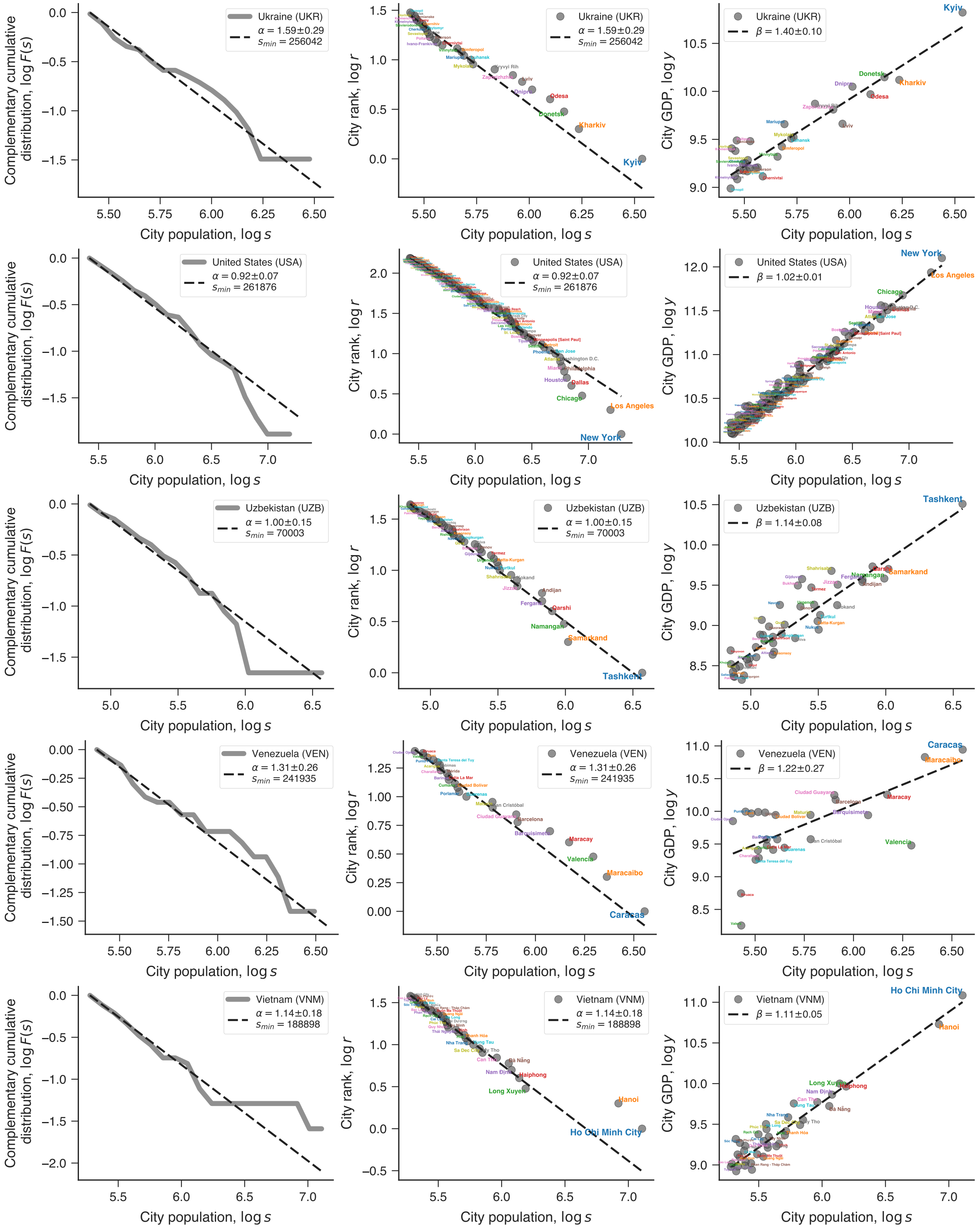

Complementary cumulative distribution, log $F(s)$
City rank, log r
City GDP, log y
City population, log s
Ukraine (UKR)
$\alpha = 1.59 \pm 0.29$
$s_{min} = 256042$
$\beta = 1.40 \pm 0.10$
United States (USA)
$\alpha = 0.92 \pm 0.07$
$s_{min} = 261876$
$\beta = 1.02 \pm 0.01$
Uzbekistan (UZB)
$\alpha = 1.00 \pm 0.15$
$s_{min} = 70003$
$\beta = 1.14 \pm 0.08$
Venezuela (VEN)
$\alpha = 1.31 \pm 0.26$
$s_{min} = 241935$
$\beta = 1.22 \pm 0.27$
Vietnam (VNM)
$\alpha = 1.14 \pm 0.18$
$s_{min} = 188898$
$\beta = 1.11 \pm 0.05$
Kyiv
Kharkiv
Odesa
Donetsk
Dnipro
Lviv
New York
Los Angeles
Chicago
Tashkent
Samarkand
Namangan
Caracas
Maracaibo
Valencia
Ho Chi Minh City
Hanoi
Haiphong

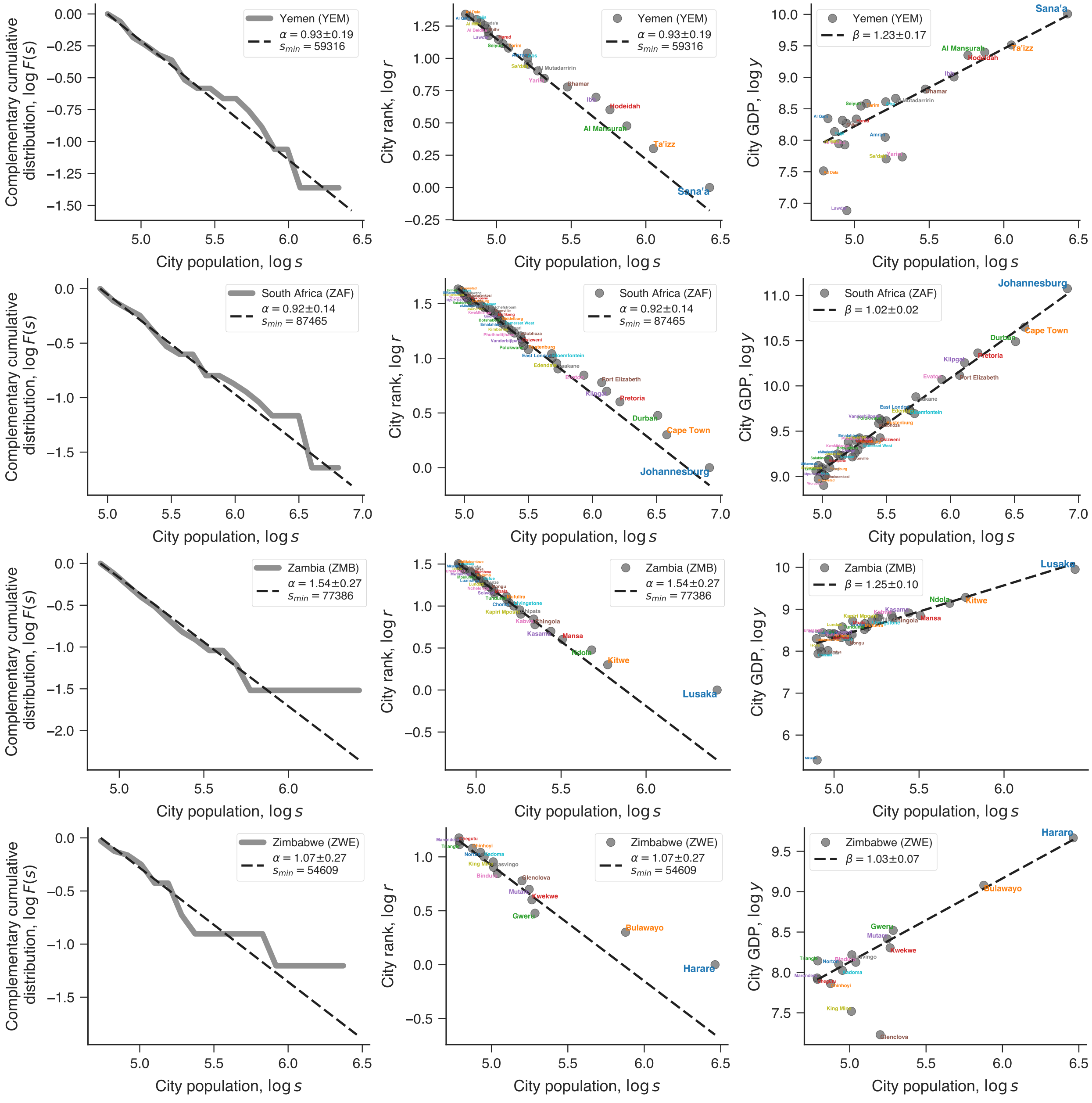
Complementary cumulative distribution, log $F(s)$
City rank, log r
City GDP, log y
City population, log s
Yemen (YEM)
$\alpha = 0.93 \pm 0.19$
$s_{min} = 59316$
$\beta = 1.23 \pm 0.17$
Sana'a
Ta'izz
Hodeidah
Al Mansurah
Dhamar
Ibb
Yarim
South Africa (ZAF)
$\alpha = 0.92 \pm 0.14$
$s_{min} = 87465$
$\beta = 1.02 \pm 0.02$
Johannesburg
Cape Town
Durban
Pretoria
Port Elizabeth
Klipgat
Zambia (ZMB)
$\alpha = 1.54 \pm 0.27$
$s_{min} = 77386$
$\beta = 1.25 \pm 0.10$
Lusaka
Kitwe
Ndola
Mansa
Kasama
Chingola
Zimbabwe (ZWE)
$\alpha = 1.07 \pm 0.27$
$s_{min} = 54609$
$\beta = 1.03 \pm 0.07$
Harare
Bulawayo
Gweru
Kwekwe
Mutare
Glenclova
King Mine
